# Effects of life-long hyperlipidaemia on age-dependent development of endothelial dysfunction in humanised dyslipidaemic mice

**DOI:** 10.1007/s11357-025-01578-w

**Published:** 2025-04-17

**Authors:** Anna Bar, Piotr Berkowicz, Anna Kurpinska, Tasnim Mohaissen, Agnieszka Karaś, Patrycja Kaczara, Joanna Suraj-Prażmowska, Magdalena Sternak, Brygida Marczyk, Agata Malinowska, Agnieszka Kij, Agnieszka Jasztal, Izabela Czyzynska-Cichon, Elsbet J. Pieterman, Hans M. G. Princen, Jacek R. Wiśniewski, Stefan Chlopicki

**Affiliations:** 1 Jagiellonian University, Jagiellonian Centre for Experimental Therapeutics (JCET), Bobrzynskiego 14, 30-348 Krakow, Poland; 2 University of Copenhagen, Department of Biomedical Sciences, Faculty of Health and Medical Sciences, Blegdamsvej 3B, 2200 København Copenhagen, Denmark; 3 Jagiellonian University Medical College, Faculty of Medicine, Grzegorzecka 16, 31-531 Krakow, Poland; 4 Polish Academy of Sciences, Mass Spectrometry Laboratory, Institute of Biochemistry and Biophysics, Pawińskiego St 5a, 02-106 Warsaw, Poland; 5 The Netherlands Organisation of Applied Scientific Research (TNO), Metabolic Health Research, Gaubius Laboratory, 2333 CK Leiden, The Netherlands; 6 Max Planck Institute of Biochemistry, Department of Proteomics and Signal Transduction, Am Klopferspitz 18, 82152 Planegg Martinsried, Germany

**Keywords:** Endothelial dysfunction, Ageing, APOE^∗^3-Leiden.huCETP mice, Magnetic resonance imaging, Hyperlipidaemia

## Abstract

Little is known, how life-long hyperlipidaemia affects vascular ageing, before atherosclerosis. Here, we characterise effects of mild, life-long hyperlipidaemia on age-dependent endothelial dysfunction (ED) in humanised dyslipidaemia model of E3L.CETP mice. Vascular function was characterised using magnetic resonance imaging in vivo and wire myograph ex vivo. Plasma endothelial biomarkers and non-targeted proteomics in plasma and aorta were analysed. Early atherosclerosis lesions were occasionally present only in 40-week-old or older E3L.CETP mice. However, age-dependent ED developed earlier, in 14-week-old male and 22-week-old female E3L.CETP mice as compared with 40-week-old female and male C57BL/6J mice. Acetylcholine-induced vasodilation in 8-week-old E3L.CETP, especially female mice, was blocked by catalase and attributed to H_2_O_2_. In 8-week-old female E3L.CETP mice, changes in plasma proteome in response to hyperlipidaemia were modest, while in male mice a number of differentially expressed proteins were identified that were involved in oxidative stress response, inflammation and regulation of metabolic pathways. In contrast, in older E3L.CETP and C57BL/6J mice, either plasma or aortic proteome displayed similar pattern of vascular ageing, dominating over hyperlipidaemia-induced changes. Interestingly, in 48-week-old male but not female E3L.CETP mice, vascular mitochondrial functional response was impaired. Early resilience of hyperlipidaemia-induced detrimental effects in young female E3L.CETP mice on a functional level was associated with a switch in vasodilation mechanism, blunted systemic proteomic response in plasma and slower ED development as compared to male E3L.CETP mice. The results indicate that profile of early vascular response to risk factors in young age may determine level of ED in older age before atherosclerosis development.

**Supplementary Information:**

The online version contains supplementary material available at 10.1007/s11357-025-01578-w.

## Introduction

Endothelial dysfunction induced by hyperlipidaemia or other risk factors represents a hallmark of many cardiovascular diseases [1–3] and has pathophysiological, prognostic and therapeutic significance [4–7]. Ageing represents the major driver of deterioration of endothelial function, vascular stiffness and of cardiovascular diseases in humans [8, 9] and may contribute to cardiovascular risk before atherosclerosis [10, 11]. Therefore, studies on the mechanism of endothelial dysfunction induced by the risk factors of cardiovascular disease should not be experimentally separated from ageing. Yet, little is known, about how life-long hyperlipidaemia affects vascular ageing prior to atherosclerosis.

In a recently published clinical study [11], using coronary computed tomography angiography, it was demonstrated that in a random sample of over 25,000 individuals of the middle-aged general population with borderline lipid values on average, silent coronary atherosclerosis was very common (42.1%). These results underscored the importance of vascular ageing as a predisposing factor for subclinical atherosclerosis development, the latter was recently reported to be independently associated with all-cause mortality in asymptomatic individuals [12].

In a clinical scenario, age-dependent progression of vascular dysfunction often coexists with hyperlipidaemia or other risk factors and there is a lack of reliable murine models that recapitulate the long-term development of age-dependent endothelial dysfunction in the setting of humanised dyslipidaemia without atherosclerosis. Moreover, the majority of preclinical studies exploring the mechanisms of cardiovascular ageing have been performed in healthy animals [13, 14] with a limited number of studies with comparative longitudinal analysis of the time course of cardiovascular ageing in both male and female mice [15]. On the other hand, in most of previous studies, the effect of hyperlipidaemia was studied in young animals mostly with the aim to dissect the mechanisms of atherosclerotic plaque development in young animals [16]. Importantly, vascular ageing may alter the endothelial response to hypercholesterolaemia [17] but again effects of hypercholesterolaemia are in general investigated in young mice.

Taken together, it is clear that longitudinal studies using models with combined influence of ageing and other cardiovascular risk factors on the vascular wall in vivo may provide unprecedented pathophysiological insight in the mechanism of accelerated vascular ageing, in particular if the model is relevant to the middle-aged human population before significant atherosclerosis development is apparent.

Most murine models in which impairment of the endothelial function in response to hyperlipidaemia has been demonstrated have a different lipid profile compared to humans, suggesting that the translational value of the longitudinal studies using classical murine models of hyperlipidaemia might be limited.

Rodent lipoprotein metabolism, unlike humans, is characterised by the fast clearance of apoB-containing lipoproteins and the absence of the cholesteryl ester transfer protein (CETP) resulting in a higher proportion of high-density lipoprotein cholesterol (HDL-C) relative to low-density lipoprotein cholesterol (LDL-C) [18]. The CETP is responsible for transferring cholesterol ester from HDL-C to the apolipoprotein B (apoB)-containing lipoproteins in exchange for triglycerides (TG) [18–21]. In contrast to rodents, human and non-human primates have a more atherogenic lipid profile, with a higher proportion of LDL-C relative to HDL-C due to the presence of CETP [18, 21].

There are various murine models of hyperlipidaemia and atherosclerosis including apolipoprotein E-deficient (ApoE^−/−^) and low-density lipoprotein receptor-deficient (LDLR^−/−^) or double knockout (ApoE/LDLR^−/−^) mice with rodent-like lipid profile and only a few models with humanised profile of lipid profile. Among them, the E3L.CETP mouse model (the APOE*3-Leiden.huCETP double transgenic mice) is of particular interest [18, 22, 23] because E3L.CETP mice display a human-like lipoprotein metabolism, with a delayed clearance of apoB-containing lipoproteins and the presence of CETP [24]. Therefore, in E3L.CETP mice, the major part of the plasma cholesterol is contained in very-low-density lipoprotein cholesterol (VLDL-C) and VLDL-remnant particles, including LDL-C and to a lesser extent in HDL-C. Importantly, the E3L.CETP mice display a functional ApoE-LDLR-mediated clearance pathway for non-HDL-C lipoproteins in contrast to ApoE^−/−^ and LDLR^−/−^ mice [24], similar to humans, this pathway is delayed as in patients with familial dysbetalipoproteinaemia carrying the mutant ApoE*3-Leiden gene [20–22, 25].

The E3L.CETP mouse model is also much more predictive for humans than other models in terms of pharmacotherapy, since these mice respond to all hypolipidemic treatments including statins, fibrates, ezetimibe, anti-proprotein convertase subtilisin-kexin type 9 serine protease (PCSK9) and anti-angiopoietin-like 3 (ANGPTL3), monoclonal antibodies (mAbs) and niacin, in a similar way to humans [18, 22, 23, 26, 27].

Therefore, in the present work, taking advantage of the unique model of humanised dyslipidaemic E3L.CETP mice, we characterised longitudinally the progression of the age-dependent impairment of the endothelial function in E3L.CETP mice, fed a chow diet with the aim to define the functional and proteomic signatures of mild dyslipidaemia-induced accelerated vascular ageing in this model as compared to their background strain of C57BL/6J mice. Both males and females were examined to characterise sex-specific responses. The vascular phenotype was analysed by functional in vivo magnetic resonance imaging (MRI)-based assessment of nitric oxide (NO)-dependent vasodilatation [28, 29], by the analysis of endothelial-specific protein plasma biomarkers and global alterations in the aortic and plasma proteome with targeted and non-targeted proteomics, respectively. To confirm that the vascular phenotype in the E3L.CETP mice occurred prior to atherosclerosis, we also scrupulously analysed the presence of atherosclerotic plaques in the aortic roots and the brachiocephalic artery in the E3L.CETP mice used here for vascular studies as well as in older E3L.CETP mice to fully confirm that our results refer to pre-atherosclerotic phase of vascular ageing in humanised dyslipidaemic E3L.CETP mice.

## Methods

### *Animals*

Studies were performed with male and female 8-, 14-, 22-, 28-, 40- and 48-week-old APOE ∗ 3-Leiden.huCETP mice (E3L.CETP, obtained from The Netherlands Organisation of Applied Scientific Research (TNO), Metabolic Health Research, Leiden, Netherlands) and sex-matched C57BL/6J controls (from Janvier Labs- Rodent research models and associated services, Le Genest-Saint-Isle, France). Moreover, 16- and 18-month-old E3L.CETP male and female mice were used for the histological assessment for the presence of atherosclerotic plaques. The mice were weighed at the beginning and the end of the experiment. The size of the experimental groups are reported in the legends of the corresponding graphs. The mice were housed in collective cages, in a room with constant environmental conditions (22–25 °C, 65–75% humidity, and a 12-h light/dark cycle). Animals had ad libitum access to daily provided chow diet and water. All experiments were approved by the Local Ethics Committee of Jagiellonian University (Krakow, Poland, identification code 19/2018 date of approval: 11th January 2018) and were in accordance with the Guide for the Care and Use of Laboratory Animals of the National Academy of Sciences (NIH publication No. 85–23, revised 1996), as well as the Guidelines for Animal Care and Treatment of the European Community.

### *Assessment of insulin resistance based on glucose tolerance test*

Before the glucose tolerance test (GTT), the mice were fasted for 4 h with access to water. The blood glucose concentration was measured using a standard glucometer in a drop of blood from the tail, cut at the top, before and 15, 30, 45, 60 and 120 min after intraperitoneal glucose administration (2 g/kg, b.w.). The results are presented as the area under the curve (AUC).

### *Assessment of acetylcholine-induced vasodilationin vivoby magnetic resonance imaging*

The MRI experiments were performed using a 9.4 T scanner (BioSpec 94/20 USR, Bruker, Germany). During the MRI experiment, the mice were anaesthetized using isoflurane (Aerrane, Baxter Sp. z o. o., Poland, 1.5 vol%) in an oxygen and air (1:2) mixture and imaged in the supine position. The heart function (rhythm and ECG), respiration and body temperature (maintained at 37 °C using circulating warm water) were monitored using a monitoring and gating System (SA Inc., Stony Brook, NY, USA).

The endothelial function in vivo was assessed with two techniques described previously: an endothelium-dependent response to acetylcholine (Ach, Sigma-Aldrich, Poznan Poland: 50 μL, 16.6 mg/kg, b.w., i.p.) in the abdominal (AA) and thoracic aorta (TA) and flow-mediated dilatation (FMD) in response to reactive hyperaemia in the femoral artery (FA), after vessel occlusion, induced by a home-made vessel occluder [28–30]. In separate group of mice, measurements of vascular response were also performed in the presence of N-nitro-L-arginine methyl ester (L-NAME, 100 mg/kg, b.w., i.p.), to determine the involvement of NO in studied responses.

The vasomotor responses were examined by comparing two, time-resolved 3D images of the vessels prior to and 30 min after intraperitoneal Ach administration or after 5 min of vessel occlusion. Images were acquired using the cine IntraGate™ FLASH 3D sequence and reconstructed with the IntraGate 1.2.b.2 macro (Bruker). An analysis was performed using ImageJ software 1.46r (NIH Bethesda, MD, USA) and scripts written in Matlab (MathWorks, Natick, MA, USA).

The imaging parameters included the following: repetition time (TR) 6.4 ms, echo time (TE) 1.4 ms, field of view (FOV) 30 × 30 × 5 mm^3^ for the FA and 30 × 30 × 14 mm^3^ for the aorta, matrix size 256 × 256 × 30 for the FA and 256 × 256 × 35 for the aorta, flip angle 30° and number of accumulations 15, reconstructed to one (FA) or seven (aorta) cardiac frames. The total scan time was from 10 to 13 min.

### *Blood sampling for biochemical analysis, tissue collection*

The mice were euthanized (100 mg/kg b.w. ketamine + 10 mg/kg b.w. xylazine, i.p.) and blood was drawn from the heart and collected in tubes containing 10% solution of ethylenediaminetetraacetic acid dipotassium salt (K_2_EDTA, Aqua-Med, Łodz, Poland, 1 μL of K_2_EDTA/100 μL of blood). Next, part of blood samples were mixed with MS-SAFE Protease and Phosphatase Inhibitor Cocktail (PIC, Sigma-Aldrich, Poznan, Poland) in a ratio of 100:1 and centrifuged at 664 × g, at a temperature of 4 °C for 10 min to isolate the plasma.

The obtained plasma samples were deep-frozen at − 80 °C for a lipid profile analysis with a biochemical analyser (ABX Pentra 400—Horiba Medical, Kyoto, Japan), micro liquid chromatography/mass spectrometry–multiple reaction monitoring (microLC/MS-MRM)-based measurements of the biomarkers of the endothelial dysfunction and total protein approach (TPA)-based mouse plasma proteomics.

After blood collection, the brachiocephalic artery (BCA) and the aortic root were isolated and fixed in a 4% buffered formalin solution for the assessment of the atherosclerotic plaque. Next, from the same mice, the aorta was isolated and cleared from the surrounding tissue. The thoracic parts of the aorta were dedicated for analysis of vascular energy metabolism using the Seahorse XFe96 Analyzer. In addition, aortas from three other batches of mice were collected: (1) to assess endothelial function assessment in isolated rings from the TA ex vivo, (2) to measure hydrogen peroxide (H_2_O_2_) production in whole aorta with Amplex Red and (3) for TPA-based aortic proteomics.

### *Assessment of acetylcholine-induced vasodilation inisolated aorta* *ex vivo*

The TA was quickly removed, and after cleaning, it was cut into four rings that were each approximately 3 mm in length. To assess the involvement of H_2_O_2_ in vasodilator responses, before measurements, the rings were incubated for 60 min in 200 µL of catalase-polyethylene glycol (PEG-catalase, 2400 U/mL; 2 rings) or in a Krebs buffer (in mM: NaCl 118.0, CaCl_2_ 2.52, MgSO_4_ 1.16, NaHCO_3_ 24.88, KH_2_PO_4_ 1.18, KCl 4.7, glucose 10.0, pyruvic acid 2.0; 2 rings). The vascular rings were transferred to organ-bath chambers filled with 5 mL of Krebs buffer. The rings were mounted between two pins of a wire myograph system (620 M, Danish Myo Technology, Denmark) that was connected to a recorder (LabChart software) in order to continuously record the tension [31, 32]. Following that, the tension of the rings was increased stepwise to reach 10 mN and the rings were incubated in Krebs buffer for another 30 min. The viability of the vessels was documented by a contractile response to potassium chloride (KCl, 30 mM, 60 mM) and phenylephrine (Phe, 3 µM) to obtain the maximal possible constriction of the rings. The aortic rings were then precontracted with Phe to obtain 80–90% of the maximal contraction. The endothelium-dependent vasorelaxation response was assessed using Ach (10 µM). Additionally, the endothelium-independent vasorelaxation was evoked by sodium nitroprusside (SNP, 1 µM). All the solutions were prepared before the experiment. The Ach, Phe, and SNP were purchased from Sigma-Aldrich (Poznan, Poland) and diluted in distilled water. The results are presented as an area above the curve (AAC) calculated from the curves of the aortic rings relaxation.

### *Measurement of hydrogen peroxide production in the aorta*

The H_2_O_2_ levels were measured in the aorta with Amplex Red (Amplex™ Red Hydrogen Peroxide/Peroxidase Assay Kit, Invitrogen, Waltham, MA, USA). In brief, to remove the red blood cells, the aorta was perfused through the left ventricle with phosphate-buffered saline (PBS) prior to tissue isolation and rinsed with ice-cold hypertonic saline (3% sodium chloride). Then, the aorta was cleaned from the surrounding tissue, dry-weighed and homogenised in 100 μL of lysis buffer (tissue protein extraction reagent, T-PER, Thermo Scientific, Waltham, MA, USA) with the addition of phosphatase (phosphatase inhibitor cocktail, 1:1000, Sigma-Aldrich, Poznan, Poland) and protease inhibitors (cOmplete™, EDTA-free Protease Inhibitor Cocktail, 1 tabl./25 mL, Sigma-Aldrich, Poznan, Poland). The homogenates were centrifuged (at 1000 × g for 5 min at 4 °C) and the supernatants were collected. Confirmation that H_2_O_2_ was the oxidant responsible for the fluorescence signal was accomplished with the inhibition of the fluorescence by exposure to catalase (preincubation for 10 min at room temperature in the assay buffer in the presence or absence of catalase from a bovine liver, Sigma-Aldrich, Poznan, Poland; 500 U/mL). The fluorescence measurements of H_2_O_2_ were performed in black 96-well plates, using a Biotek Synergy 4 Hybrid Multi-Mode Microplate Reader (fluorescence excitation 560 nm and fluorescence emission 590 nm), for 30 min starting immediately after the addition of the working solution (assay buffer containing Amplex Red; 0.1 mM and horseradish peroxidase; 0.032U/mL). The results were presented as AUC and normalised to the tissue weight.

### *Assessment of vascular bioenergy functional profile*

The vascular bioenergetic parameters were assessed using the Seahorse XFe96 Analyzer and Seahorse Spheroid Microplates (Agilent, USA) in isolated aortic rings as described previously [33]. Briefly, the TA was isolated, cleaned and incubated for 24 h with interleukin-1β (IL-β, 1 ng/mL) in minimum essential medium (MEM, Sigma) supplemented with 0.1% One Shot fetal bovine serum (FBS, Gibco), MEM non-essential amino acid solution (Sigma), MEM vitamin solution (Sigma) and 1% antibiotic–antimycotic (Gibco). Prior to measurement, aortic fragments were cut into approximately 1-mm-long rings and each ring was placed in a well of Seahorse XFe96 Spheroid Microplate in mitochondrial stress test (MST) assay medium. Oxygen consumption rate (OCR) and the extracellular acidification rate (ECAR) were measured using the MST protocol, after sequential additions of oligomycin (10 μg/mL; Sigma-Aldrich, USA), FCCP (carbonyl cyanide 4 [trifluoromethoxy] phenylhydrazone; 1 μM; Sigma-Aldrich, USA) and rotenone (5 μM; Sigma-Aldrich, USA) with antimycin A (5 μM; Sigma-Aldrich, USA). Data were recorded and analysed using Wave software (Agilent, USA) and results were normalised for total protein content in each aortic ring. Calculations of bioenergetic parameters were performed using the Agilent MST User Guide.

### *Assessment of biomarkers of endothelial dysfunction in plasma*

The assessment of protein/peptide biomarkers of the endothelial dysfunction in plasma was performed using a microLC/MS-MRM-based method, as described previously [5, 30, 34–36]. The panel included biomarkers of various aspects of the endothelial dysfunction such as the glycocalyx disruption: syndecan-1 (SDC-1) and endocan (ESM-1); endothelial inflammation: soluble form of vascular cell adhesion molecule 1 (sVCAM-1), soluble form of E-selectin (sE-sel), soluble form of P-selectin (sP-sel) and soluble form of intercellular adhesion molecule 1 (sICAM-1); endothelial permeability: angiopoietin 1 (Angpt-1), angiopoietin 2 (Angpt-2), soluble form of fms-like tyrosine kinase 1 (sFLT-1) and soluble form of the receptor for angiopoietin 1 (sTie-2); hemostasis: von Willebrand factor (vWF), tissue plasminogen activator (t-PA) and plasminogen activator inhibitor 1 (PAI-1), thrombospondin 1 (THBS-1) and thrombin activatable fibrinolysis inhibitor (TAFI); and other biomarkers: adrenomedullin (ADM), adiponectin (ADN) and annexin A5 (ANXA5).

An UPLC Nexera system (Shimadzu, Kyoto, Japan) connected to a highly sensitive mass spectrometer QTrap 5500 (Sciex, Framingham, MA, USA) was used. During sample preparation, the murine plasma was subjected to proteolytic digestion using porcine trypsin to achieve unique and reproducible peptide sequences that were applied as the surrogates of the proteins suitable for LC–MS analyses. Detailed descriptions of the targeted analysis of a selected panel of proteins have been presented elsewhere [34–36].

### *Histological assessment of atherosclerotic plaques*

For the determination of the atherosclerotic plaque area, the isolated BCA and aortic root were dissected, fixed in 4% buffered formalin and embedded in paraffin. Five-micrometer-thick serial sections of BCA and all sections from the entire aortic root, showing the valve sinuses (from the annulus to the place, where the fully-formed aortic wall was visible around the whole circuit), were collected. Our originally developed staining with Unna’s orcein combined with Martius, Scarlet and Blue trichrome (OMSB), as described recently [37], was applied to all sections. Ten cross-sections of the BCA and six cross-sections of the aortic root were placed on each slide for the visualisation of the atherosclerotic plaque, foam cells and adhesion of leukocytes to the endothelial surface collagen, elastin, fibrin, red blood cells and vascular smooth muscle cells within the atherosclerotic plaque. The detection of atherosclerotic plaque and other histological changes was performed under a microscope (Olympus) with 400 × magnification.

### *Globalproteomic analysis in plasma*

Plasma samples were diluted ten fold with a buffer containing 0.1 M Tris–HCl, pH 7.8, 50 mM DL-dithiotreitol (DTT, BioShop, Burlington, Ontario, Canada) and 1% sodium dodecyl sulphate (SDS, Sigma Aldrich, Saint Louis, MO, USA) and incubated in a boiling-water-bath for 5 min. The lysates were processed using the multienzyme digestion (MED) filter-aided sample preparation (FASP) procedure in a buffer containing 1.0 mM DTT [38]. The proteins were consecutively digested using LysC, trypsin and chymotrypsin and collected in separate fractions. Before sample desalting, the isolated peptides were incubated with an additional portion of DTT at a concentration of 10.0 mM for 20 min. Total protein concentration in lysates and the peptide contents in the digests were assayed using the tryptophan fluorescence-based WF-assay using tryptophan as a standard [39]. Peptides were analysed by LC–MS/MS, as described previously [38]. The obtained spectra were searched using the MaxQuant software. Proteins were quantified using the TPA approach [40]. A statistical analysis of the results was performed using the Perseus software program. The mass spectrometry data were deposited to the ProteomeXchange Consortium via the PRIDE partner repository with the dataset identifier PXD029562. The data were analysed in the STRING program [41, 42]. Statistical analysis was based on *t*-test with correction for multiple comparisons. All data analysis included only these proteins that were significantly different between compared groups.

### *Globalproteomic analysis in the aorta*

Label-free non-targeted differential proteomic analysis was performed to assess protein profile changes. The proteins were subjected to reduction, alkylation and two-stage enzymatic digestion (LysC, trypsin) using the modified FASP method [43]. Samples were analysed using LC–MS system. LC–MS measurements were performed in the Mass Spectrometry Laboratory at the Institute of Biochemistry and Biophysics, Polish Academy of Sciences, Warsaw, Poland.

In details, aorta homogenization was performed in a buffer containing 2% SDS, 0.05 M DTT in 0.1 M Tris–HCl (pH 7.6, BioShop, Burlington, Ontario, Canada) followed by sonication on ice, boiling for 5 min in 99 °C, centrifugation (16,000 × g, 15 min, 4 °C), and collection of the supernatants. Protein concentration was assessed using Pierce 660 protein assay reagent (Thermo Scientific, USA) following the manufacturer’s protocol. Samples were prepared according to modified FASP protocol [43]. Microcon Ultracel 30 kDa (Merck, Darmstadt) filters were prepared by double spinning using 14,000 × g, 15 min 20 °C with 8 M urea in 100 mM Tris–HCl (pH 8,UA, uronic acid). Aliquots of 50 µg of protein were mixed with UA and transferred into individual filters. After centrifugation, an additional 200 µL of UA was added to the filters and centrifuged again. Disulphide bonds were reduced with 50 µL of 50 mM DTT in UA (56 °C, 20 min) and alkylated with 50 µL of 50 mM iodoacetamide (Sigma-Aldrich, Saint Louis, MO, USA) in UA (protected from light, 20 °C, 20 min), each time centrifuged as mentioned above. Double washing step with 100 µL of UA and double washing step with 100 µL of 40 mM ammonium bicarbonate (ABC, Sigma-Aldrich, Saint Louis, MO, USA) were applied. Proteins were first digested with Lys-C (18 h, 37 °C, 1:100; New England BioLabs, Ipswich, MA). Digested peptides and eluate after rinsing with 40 mM ABC were combined and collected (10,000 × g, 10 min, RT). Digestion was quenched with 0.5% trifluoroacetic acid (Sigma Aldrich, Germany). The remaining proteins on the filter were subsequently digested with trypsin (3 h, 37 °C, 1:100; Promega, Madison, WI) as specified above for LysC. Eluates were further processed after combining. Peptides were purified using Pierce Peptide Desalting Spin Columns according to manufacturer’s instruction (Thermo Scientific, USA). Peptide concentration was assessed using modified BCA assay kit (Thermo Scientific, Germany).

Samples were analysed using the LC–MS system composed of Evosep One (Evosep Biosystems, Odense, Denmark) directly coupled to a Orbitrap Exploris 480 mass spectrometer (Thermo Scientific, USA). Peptides were loaded onto disposable Evotips C18 trap columns (Evosep Biosystems, Odense, Denmark) according to the manufacturer’s protocol with some modifications aimed to prevent sample drying and emitter contamination. Briefly, Evotips were activated with 25 µL of Evosep solvent B (0.1% formic acid in acetonitrile, Thermo Scientific, USA) by 1 min centrifugation at 600 × g followed by 2 min incubation in 2-propanol (Thermo Scientific, USA). After equilibration with 25 µL of solvent A (0.1% formic acid in H_2_O, Thermo Fisher Scientific, Waltham, MA, USA), 2 µg of each peptide sample was loaded onto the solid phase. Precolumns were washed 3 times with 100 µL 2% acetonitrile (Thermo Scientific, USA) in solvent A and covered with 300 µL of solvent A. Chromatography was carried out at a flow rate 220 nL/min using the 88 min (15 samples per day) preformed gradient on EV1106 analytical column (Dr Maisch C18 AQ, 1.9 µm beads, 150 µm ID, 15 cm long, Evosep Biosystems, Odense, Denmark). Data was acquired in positive mode with a data-dependent method using the following parameters. MS1 resolution was set at 60,000 with a normalised AGC target of 300%, auto maximum inject time and a scan range of 300 to 1600 m/z. For MS2, resolution was set at 15,000 with a standard normalised AGC target, auto maximum inject time and top 40 precursors within an isolation window of 1.6 m/z considered for MS/MS analysis. Dynamic exclusion was set at 20 s with allowed mass tolerance of ± 10 ppm and the precursor intensity threshold at 5e3. Precursors were fragmented in HCD mode with a normalised collision energy of 30%.

Raw data were processed with MaxQuant suite (version 2.1.1.0) (Max Planck Institute of Biochemistry, Martinsried, Germany) [44, 45] using Mus musculus database from Swissprot (version 03_2022), with ‘Match between runs’ option enabled. Fixed modification was carbamidomethyl (C), variable—oxidation (M) and acetyl (Protein N-term). Further analysis was performed in Perseus (version 1.6.15) software (Max Planck Institute of Biochemistry, Martinsried, Germany) [46]. Proteins from reversed database, identified by site, and contaminations were removed. Data were log transformed, missing values were imputed from normal distribution and normalisation on median was performed. Statistical analysis was based on *t*-test with correction for multiple comparisons. All data analysis included only these proteins that were significantly different between compared groups. Proteomic datasets were deposited to the RODBUK Cracow Open Research Data Repository (10.57903/UJ/PGQTUF).

Further biostatistical analysis and visualisation of the obtained results were performed, among others, by using String DB, ShinyGO, Reactome, KEGG database, GraphPad Prism (GraphPad Software, Inc., USA) and InteractiVenn [41, 47–49].

### *Statistical analysis*

The obtained data are presented as the mean and standard deviation, or in the case of the lack of normal distribution, as the median and interquartile range. Normal distribution of data was assessed by Shapiro–Wilk test. Unless otherwise stated, statistical tests were performed using GraphPad Prism (GraphPad Software, Inc., USA). Non-parametric (Kruskal–Wallis test) or parametric (two-way analysis of variance (ANOVA) with honest significant difference (HSD) Tukey’s test for unequal sample sizes or Student’s *t*-test) tests were performed. A value of *p* < 0.05 was considered to be statistically significant.

## Results

### *General characteristics of 8–40-week-old E3L.CETP mice*

The E3L.CETP mice did not become obese (Fig. S1A), as evidenced by the lack of differences in body weight between E3L.CETP mice vs sex- and age-matched control C57BL/6J mice. The difference in the body weight of the mice was only observed between male and female mice, and this difference pertained to both the E3L.CETP and C57BL/6J mice strain. The E3L.CETP mice did not display insulin resistance (Fig. S1B), as evidenced by the lack of changes in the level of blood glucose concentration in the GTT. The hyperlipidaemia in the E3L.CETP mice was manifested by an increased plasma level of TG (2.20 vs 1.00 mmol/L, Fig. S1D) and non-HDL cholesterol (0.8 vs 0.17 mmol/L, Fig. S1E), as well as a decreased level of HDL cholesterol (0.15 vs 0.80 mmol/L, Fig. S1F) resulting in a slightly increased level of total cholesterol (2.50 vs 2.00 mmol/L, Fig. S1C) in comparison to the C57BL/6J mice. The lipid profile for the E3L.CETP mice mimicked the human lipid profile and differed with a lipid profile of control mice as shown in Fig. S1G.

Importantly, advanced atherosclerotic plaques were not observed in either the aortic root (Fig. S2A–E, G–H) or in the BCA (Fig. S2F). This was confirmed in the 40-week-old E3L.CETP as well as 16–18-month-old E3L.CETP male and female mice fed a chow diet. Of note, characteristic early lesions of the atherosclerosis with fibrin deposition were occasionally detected in the ascending aorta within the aortic root but not in the BCA (Fig. S2C–E). Moreover, in 16–18-month-old E3L.CETP male mice, thus, older mice then used for characterisation of vascular responses, atherosclerotic plaques were also not present; only slight disorganisation of the elastic lamina was observed (Fig. S2H) in the wall of the ascending aorta within aortic root, without the presence of typical atherosclerotic plaques. In turn, even in 16–18-month-old E3L.CETP female mice, neointima formation and foam cells were detected, in the wall of the ascending aorta within aortic root, suggesting the presence of early atherosclerotic lesions in 16–18-month-old female E3L.CETP mice.

### *Accelerated, age-dependent impairment of acetylcholine- and flow-induced endothelial responsein 8–40-week-old E3L.CETP mice, compared toC57BL/6J mice, studied* *in vivo* *with MRI*

**Fig. 1:**
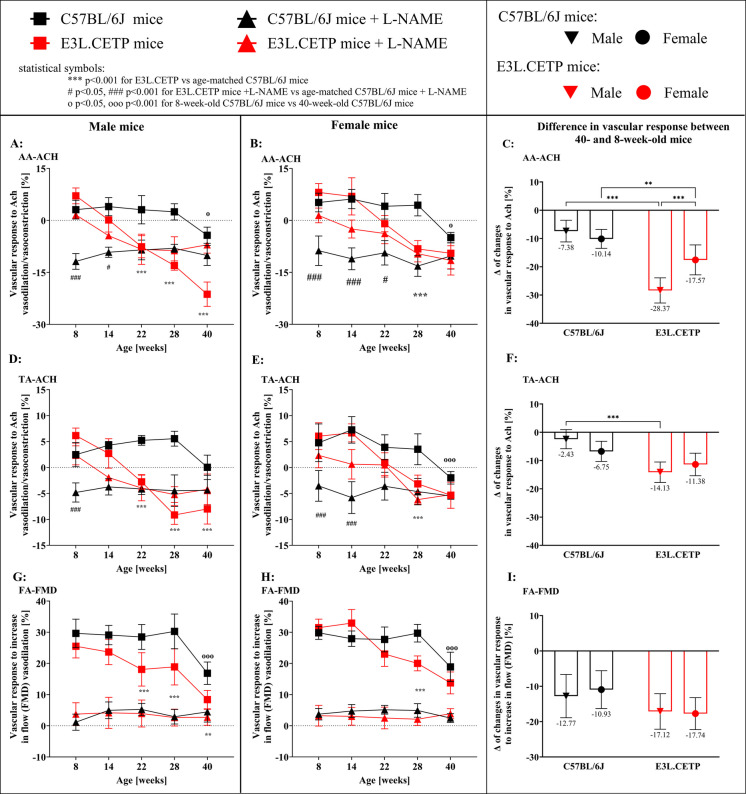
Progression of endothelial dysfunction in E3L.CETP mice. Changes in the end-diastolic volume of the abdominal aorta (AA-ACH, **A**, **B**, **C**) and the thoracic aorta (TA-ACH, **D**,** E**,** F**) 30 min after acetylcholine (Ach) administration as well as changes in the volume of the femoral artery after 5-min vessel occlusion (FA-FMD, **G**,** H**, **I**) in 8-, 14-, 22-, 28- and 40-week-old E3L.CETP male (**A**,** C**,** E**) and female (**B**,** D**,** F**) mice, in comparison to age- and gender-matched control mice (C57BL/6J). Vascular responses were studied in the presence and absence of N-nitro-L-arginine methyl ester (L-NAME), and are expressed as a percent of changes (A, B, D, E, G, H) or as delta of changes between readouts in 40-week-old and 8-week-old mice (C, F, I). Size of groups: *n* = 4–6. Data are presented as the mean and standard deviation. Statistics: two-way ANOVA followed by Tukey’s post hoc test. Statistical symbols for panels A–B, D–E, G–H: ***p < 0.001 for E3L.CETP vs age-matched C57BL/6J mice; #*p* < 0.05, ###*p* < 0.001 for E3L.CETP mice + L-NAME vs age-matched C57BL/6J mice + L-NAME; o*p* < 0.05, ooo*p* < 0.001 for 8-week-old C57BL/6J mice vs 40-week-old C57BL/6J mice

In the C57BL/6J male and female mice, endothelial dysfunction was present not earlier than in 40-week-old mice. This was evidenced by the preservation of the Ach-induced response in the TA and the AA and the FMD in the FA, in C57BL/6J mice younger than 40 weeks of age (Fig. 1).

In turn, in the E3L.CETP mice, impaired Ach-induced vasodilation was already detected in the AA in 14-week-old male and 22-week-old female mice, indicating accelerated, age-dependent endothelial dysfunction development in E3L.CETP mice vs control C57BL/6J mice. Furthermore, in the 22–40-week age range E3L.CETP male mice and in the 28–40 week age range E3L.CETP female mice, Ach-induced vasodilation was switched to paradoxical vasoconstriction in the AA (Fig. 1A–B). Importantly, the level of age-dependent endothelial dysfunction development, expressed as a delta of changes between 40-week-old and 8-week-old mice, was higher in E3L.CETP male mice than E3L.CETP female mice (− 28.37 vs − 17.57%, respectively; Fig. 1C), which eventually led to more severe endothelial dysfunction development in 40-week-old male than 40-week-old female E3L.CETP mice (− 21.3% in E3L.CETP male mice vs − 9.5% in E3L.CETP female mice, Fig. 1A–B).

In the TA, the progressive impairment of the Ach-induced vasodilation in the E3L.CETP mice had similar features to that in the AA, but was less pronounced in comparison to the impairment observed in the AA (Fig. 1D–F).

In the FA, based on FMD, the progressive impairment of the endothelial function was also observed earlier in male E3L.CETP mice than in female E3L.CETP mice and in both sexes occurred earlier then in C57BL/6J mice. FMD was already impaired in the 22-week-old E3L.CETP male mice (18.1 vs 28.5% in the age-matched C57BL/6J mice) and in 28-week-old E3L.CETP female mice (20.0 vs 29.7% in the age-matched C57BL/6J mice Fig. 1G–H). Finally, the FMD impairment was similar in the 40-week-old male and female E3L.CETP mice (8.4% for male and 13.8% for female mice vs 16–18% in the age-matched C57BL/6J mice, Fig. 1I).

In the presence of the L-NAME, the FMD response in vivo was almost completely inhibited in all age groups of the E3L.CETP male and female mice, similarly to the C57BL/6J male and female mice (Fig. 1G–H). In contrast, L-NAME displayed distinct effects on Ach-induced response in the young E3L.CETP mice vs the older E3L.CETP mice and C57BL/6J mice. In the C57BL/6J mice in either the AA or TA, the response to Ach in vivo, in the presence of L-NAME, was abrogated and turned to vasoconstriction, independently of the age of the animals. However, in the 8–22-week-old female and male E3L.CETP mice, L-NAME either had no effect or had a lesser effect on Ach-induced vascular response (Fig. 1A–B, D–E). In the 28–40-week-old, female and male E3L.CETP mice, the L-NAME effect on Ach-induced vasoconstrictor response was potentiated, similarly to that in the C57BL/6J mice. These results suggested that in the 8–22-week-old E3L.CETP mice, the Ach-induced vasodilation was independent of NO. Representative images depicting changes in the aorta cross-sections, before and after Ach administration in the presence and absence of L-NAME, are shown in Figure S3.

### *A switch fromNO-dependent toH*_*2*_*O*_*2*_*-dependentvasodilation in the aorta in 8-week-old E3L.CETP mice*

**Fig. 2:**
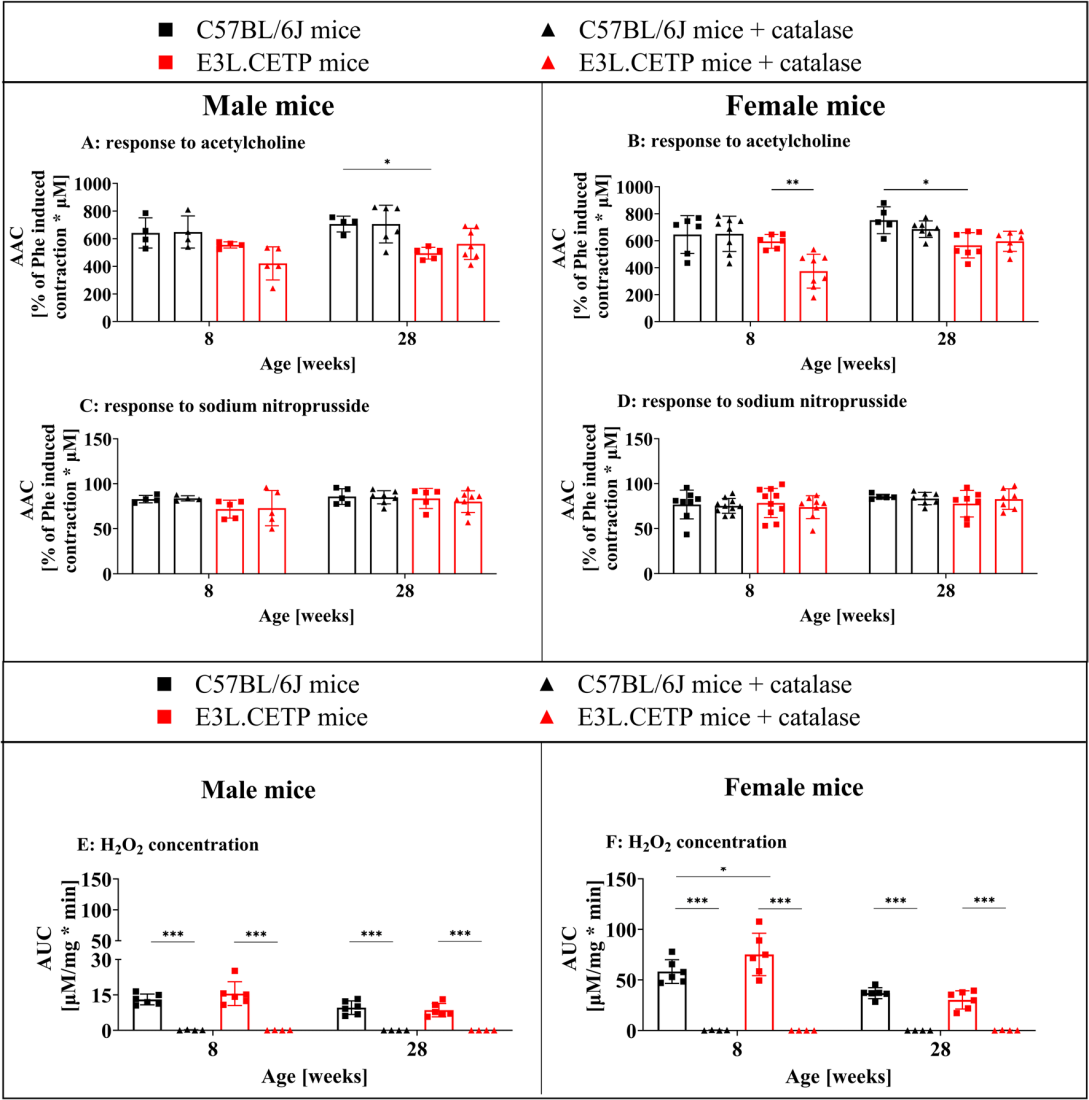
Role of H_2_O_2_ in endothelium-dependent vasodilatation in the aortic rings from E3L.CETP mice. Relaxation of aortic rings from the thoracic aorta (percent of phenylephrine (Phe, 3 µM)-induced contraction) in response to acetylcholine administration (Ach, 10 µM, **A**,** B**) and response to sodium nitroprusside administration (SNP, 1 µM, **C**,** D**) in the presence and absence of catalase–polyethylene glycol (PEG-catalase, 2400 U/mL), presented as the area above the curve (AAC) in 8- and 28-week-old E3L.CETP male (**A**,** C**,** E**) and female (**B**,** D**,** F**) mice, in comparison to age- and gender-matched control mice (C57BL/6J). Production of H_2_O_2_ in the abdominal aorta in the presence and absence of catalase (500 U/mL) is presented in **E** and **F**. Size of groups: *n* = 4–7. Data are presented as the mean and standard deviation. Statistics: two-way ANOVA followed by Tukey’s post hoc test: *p < 0.05, **p < 0.01,***p < 0.001

To further explore the mechanisms of the Ach-induced vasodilation in 8–22-week-old E3L.CETP mice, that was not inhibited by L-NAME in vivo, the isolated aorta preparation ex vivo was used (Fig. 2). Ach-induced vasodilation was studied in the isolated aorta in 8-week-old and 28-week-old male and female E3L.CETP and C57BL/6J mice. In the 28-week-old E3L.CETP mice, Ach-dependent vasodilation in the isolated aorta ex vivo was impaired in comparison to 28-week-old C57BL/6J mice, in both male and female mice (Fig. 2A–B). In turn, the endothelial function was fully preserved in the 8-week-old E3L.CETP male and female mice. Endothelium-independent response to SNP was not altered either in 8- or in 28-week-old E3L.CETP male and female mice (Fig. 2C–D).

Importantly, the addition of catalase inhibited the Ach-induced vasodilation in 8-week-old E3L.CETP mice, and this effect was mainly observed in female mice (Fig. 2B), while in male mice, only a tendency was observed (Fig. 2A). However, catalase was not effective in 28-week-old E3L.CETP mice nor in C57BL/6J mice. These results pointed to H_2_O_2_-dependent vasodilation in response to Ach, in the 8-week-old, but not in the 28-week-old E3L.CETP mice, nor in C57BL/6J mice.

Involvement of H_2_O_2_, as a possible mediator of endothelium-dependent vasodilation, in the Ach-induced vasodilation in 8-week-old E3L.CETP mice, in the isolated aorta ex vivo, was confirmed by measurements of vascular H_2_O_2_ production using Amplex Red assay. Basal level of the H_2_O_2_ in the aorta was 1.2-fold and 1.3-fold higher in 8-week-old male and female E3L.CETP mice, respectively, in comparison to C57BL/6J mice (Fig. 2E–F). On the other hand, the H_2_O_2_ level in the aorta was at the same level in 28-week-old E3L.CETP vs C57BL/6J mice. During measurements, the fluorescence intensity was fully reduced to zero by exposure to catalase, which confirmed that the H_2_O_2_ production in the aorta was responsible for the fluorescence signal.

### *Biomarkers of endothelial dysfunction in plasma in 8–28-week-old E3L.CETP mice with targeted proteomic analysis*

**Fig. 3:**
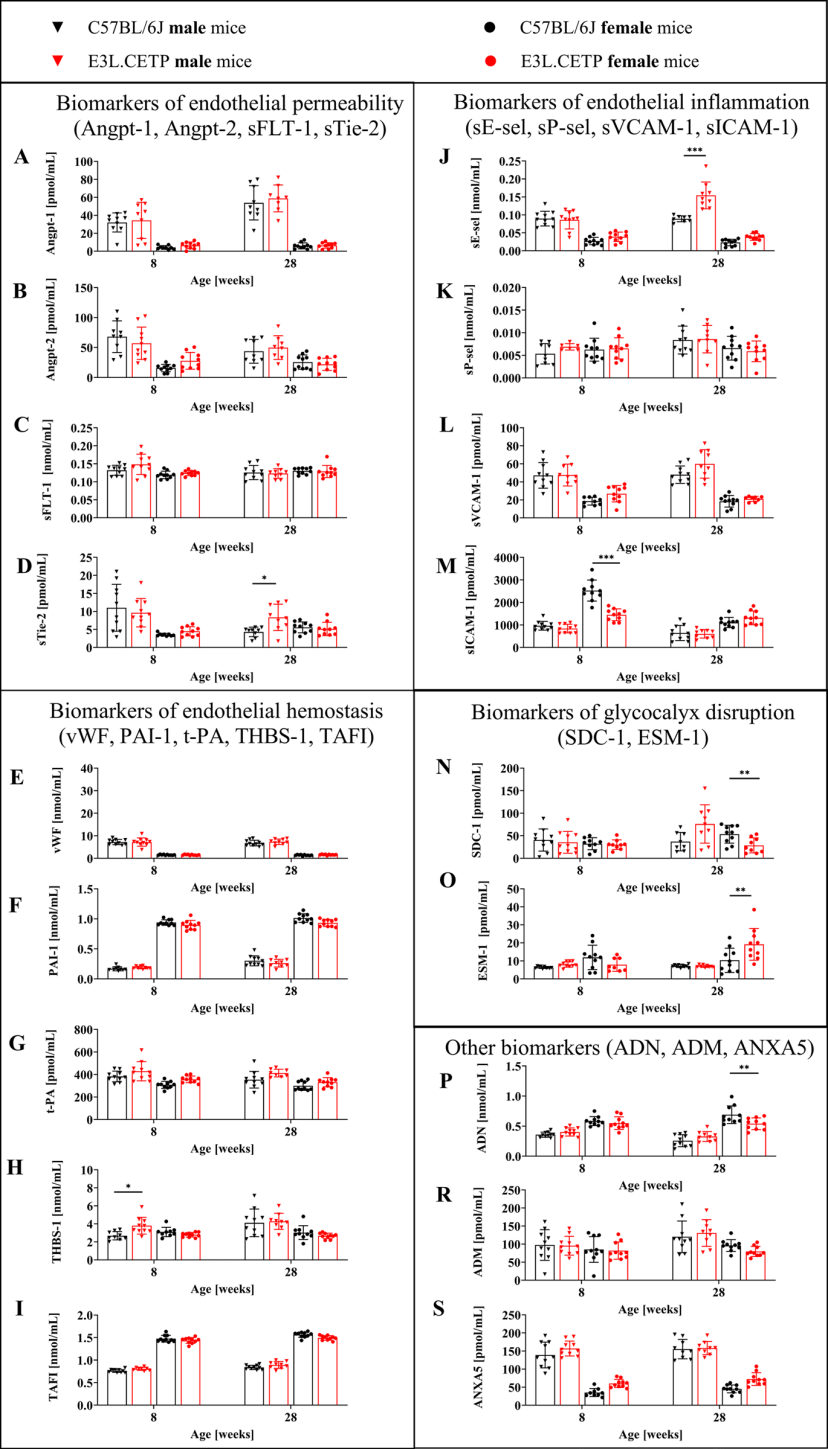
Plasma concentration of protein/peptide biomarkers of endothelial dysfunction assessed by targeted proteomic analysis. Concentration of angiopoietin 1 (Angpt-1, **A**), angiopoietin 2 (Angpt-2, **B**), soluble form of fms-like tyrosine kinase 1 (sFLT-1, **C**), soluble form of the receptor for angiopoietin 1 (sTie-2, **D**), von Willebrand factor (vWF, **E**), plasminogen activator inhibitor 1 (PAI-1, **F**), tissue plasminogen activator (t-PA,**G**), thrombospondin 1 (THBS-1, **H**), thrombin activatable fibrinolysis inhibitor (TAFI, **I**), soluble form of E-selectin (sE-sel, **J**), soluble form of P-selectin (sP-sel, **K**), soluble form of vascular cell adhesion molecule 1 (sVCAM-1, **L**), soluble form of intercellular adhesion molecule 1 (sICAM-1, **M**), syndecan-1 (SDC-1, **N**), endocan (ESM-1, **O**), adiponectin (ADN, **P**), adrenomedullin (ADM, **R**) and annexin A5 (ANXA5, **S**) in plasma, in 8- and 28-week-old E3L.CETP male and female mice, in comparison to age- and gender-matched control mice (C57BL/6J). Size of groups: *n* = 7–10. Data are presented as the mean and standard deviation (A–B, D–F, H–S) or as the median and interquartile range (C, G). Statistics: A–B, D–F, H–S: two-way ANOVA followed by Tukey’s post hoc test; C,G: Kruskal–Wallis test; *p < 0.05, **p < 0.01, ***p < 0.001

To better characterise the phenotype of accelerated, age-dependent endothelial dysfunction development in E3L.CETP mice, biomarkers of the endothelial permeability (Angpt-1, Angpt-2, sFLT-1, sTie-2; Fig. 3A–D), glycocalyx disruption (SDC-1, ESM-1; Fig. 3N–O), endothelial inflammation (sE-sel, sP-sel, sVCAM-1, sICAM-1; Fig. 3J–M), endothelial hemostasis (vWF, PAI-1, t-PA, THBS-1, TAFI; Fig. 3E–I) and other biomarkers (ADN, ADM, ANXA5; Fig. 3P–S) were analysed. With the exception of increased plasma level of sE-sel, s-Tie-2 and SDC-1 in 28-week-old male E3L.CETP mice, ESM-1 in 28-week-old female E3L.CETP mice and THBS-1 in 8-week-old male E3L.CETP mice, there were no consistent differences in plasma concentration of the biomarkers of the endothelial permeability, endothelial inflammation or hemostasis that would reflect accelerated age-dependent impairment of endothelial function detected in vivo by MRI in E3L.CETP mice, as compared with age-matched C57BL/6J mice.

### *Alterations in aortic proteome in 8-week-old and 40-week-oldE3L.CETP male and female mice ascompared to ageand sex-matched C57BL/6J mice by globalproteomic analysis*

To characterise the proteomic vascular phenotype in the aorta, associated with accelerated age-dependent endothelial dysfunction in E3L.CETP mice, multidirectional proteomic analysis was performed to characterise changes related to the ageing (8-week-old mice compared to 40-week-old mice within the same strain of mice) and to hyperlipidaemia at 8- and 40-week-old mice (comparison of E3L.CETP mice to age-matched control C57BL/6J mice). Analyses were performed separately in male and female mice (Fig. S4).

**Fig. 4:**
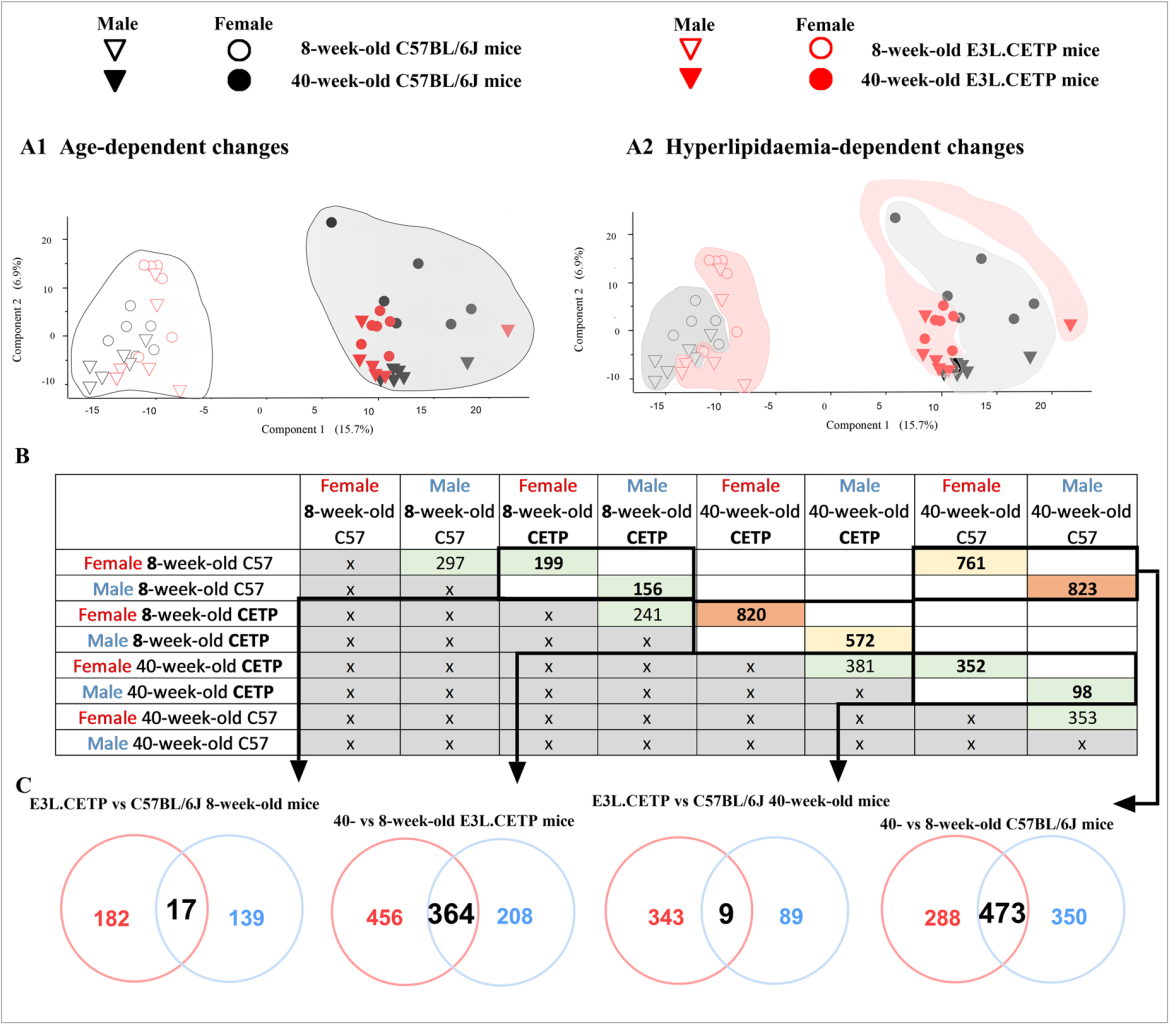
Overview of global proteomic changes in the aorta of E3L.CETP and C57BL/6J mice. Principal component analysis (PCA, **A1**–**A2**) of protein content variance in the aorta. Table (**B**) and Venn diagrams (**C**) presenting differentially expressed proteins (DEPs) in the aorta in 8- and 40-week-old female and male E3L.CETP (CETP) mice in comparison to age- and sex-matched control mice C57BL/6J (C57) as well as in ageing process in the female and male E3L.CETP and C57BL/6J mice. Size of groups: *n* = 6. Statistics: Student’s *t*-test. Legend: in Venn diagrams, red circles represent DEPs for female and blue circles for male mice. Presented PCA plots, table and Venn diagrams were prepared based only on proteins that were significantly different between compared groups

Principal component analysis (PCA) of protein content variance in the aorta (with 5189 statistically significant changes revealed in the concentrations of proteins at 5% FDR across groups of animals that were distinct in sex, lipidaemia status and age) demonstrated clear-cut separation of 8-week-old mice proteome from proteome of 40-week-old mice (Fig. 4A1). This separation was more pronounced than the hyperlipidaemia-dependent differences in proteome of E3L.CETP mice vs C57BL/6J mice, either in 8-week-old or in 40-week-old mice (Fig. 4A2). Moreover, greater impact of ageing in comparison to hyperlipidaemia on aortic proteome was reflected by number of differentially expressed proteins (DEPs): at least 572 for ageing-dependent DEPs and no higher than 352 for hyperlipidaemia-dependent DEPs (Fig. 4B–C). Similarly, in REACTOME- (Fig. S5) and STRING-based analysis (Fig. S6), effect of ageing was more pronounced than that of hyperlipidaemia, compatible with the changes detected based on PCA and analysis on number of DEPs determining ageing and hyperlipidaemia.

**Fig. 5:**
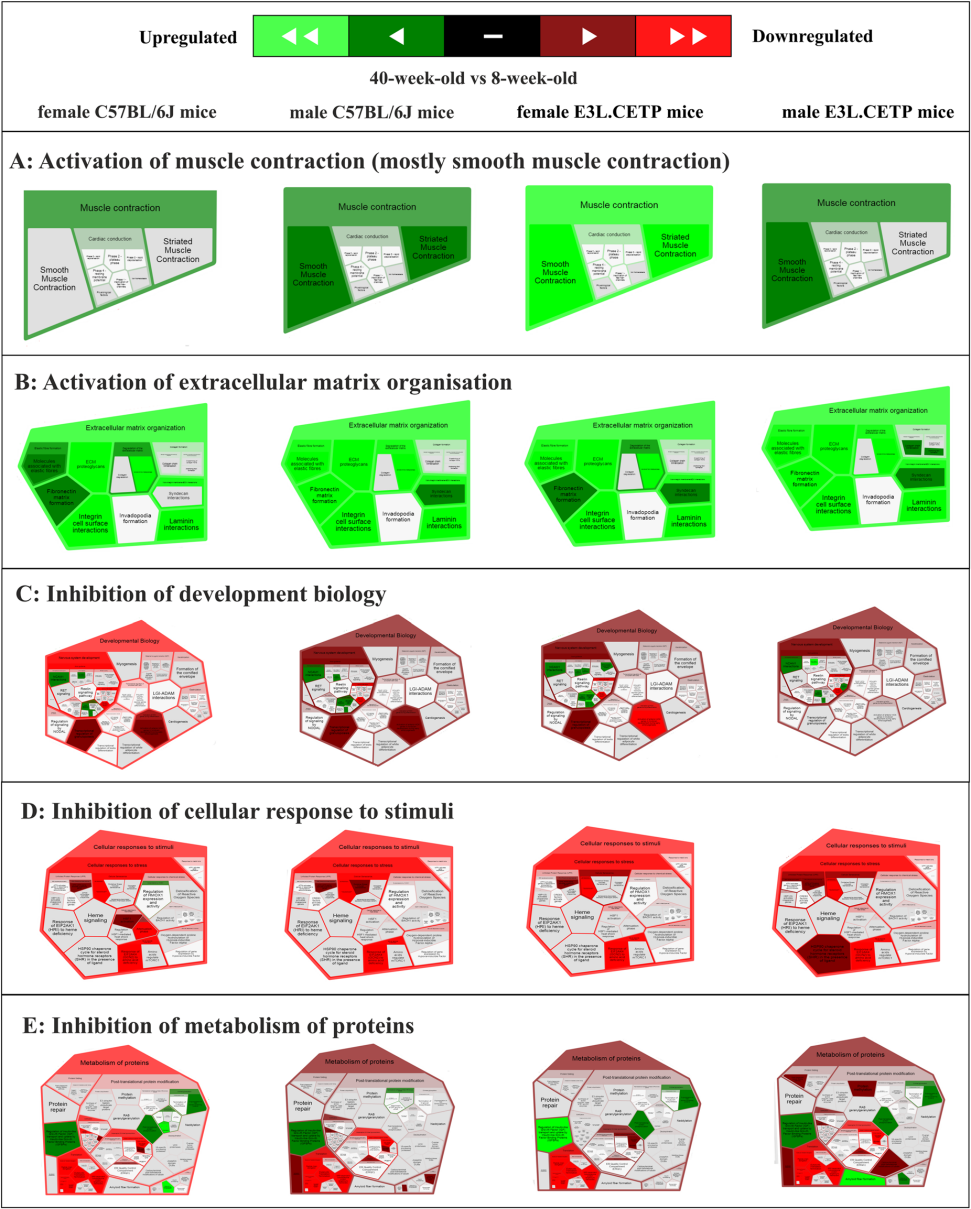
Changes in biological processes in the aorta induced by ageing independent of sex and strain of mice, based on proteomic analysis. REACTOME diagrams presenting activated (**A** muscle contraction, **B** extracellular matrix organisation) and inhibited (**C** development biology, **D** cellular response to stimuli, **E** metabolism of proteins) processes in the aorta in 40-week-old vs 8-week-old female and male C57BL/6J mice and in 40-week-old vs 8-week-old female and male E3L.CETP mice. Legend: the greener, the more activated; the redder, the more inhibited

Surprisingly, neither REACTOME-based analysis of biological processes in the aorta induced by ageing nor STRING-based analysis of DEP functional protein association networks involved in ageing did reveal any significant differences in ageing that would reflect the difference in endothelial function in E3L.CETP mice as compared to age and sex-matched C57BL/6J mice. Of note, activation of extracellular matrix organisation, activation of smooth muscle contraction, inhibition of development biology, cellular response to stimuli and inhibition of metabolism of proteins appeared in vascular ageing, independently of sex and strain, but these changes displayed similar level in all four comparisons (Fig. 5).

### *Identification of ageing markers in E3L.CETP and C57BL/6J mice as well as hyperlipidaemia markers in 8-week-old and 40-week-old E3L.CETP male and female mice, in aortic proteome by global* *proteomic* *analysis*

**Fig. 6:**
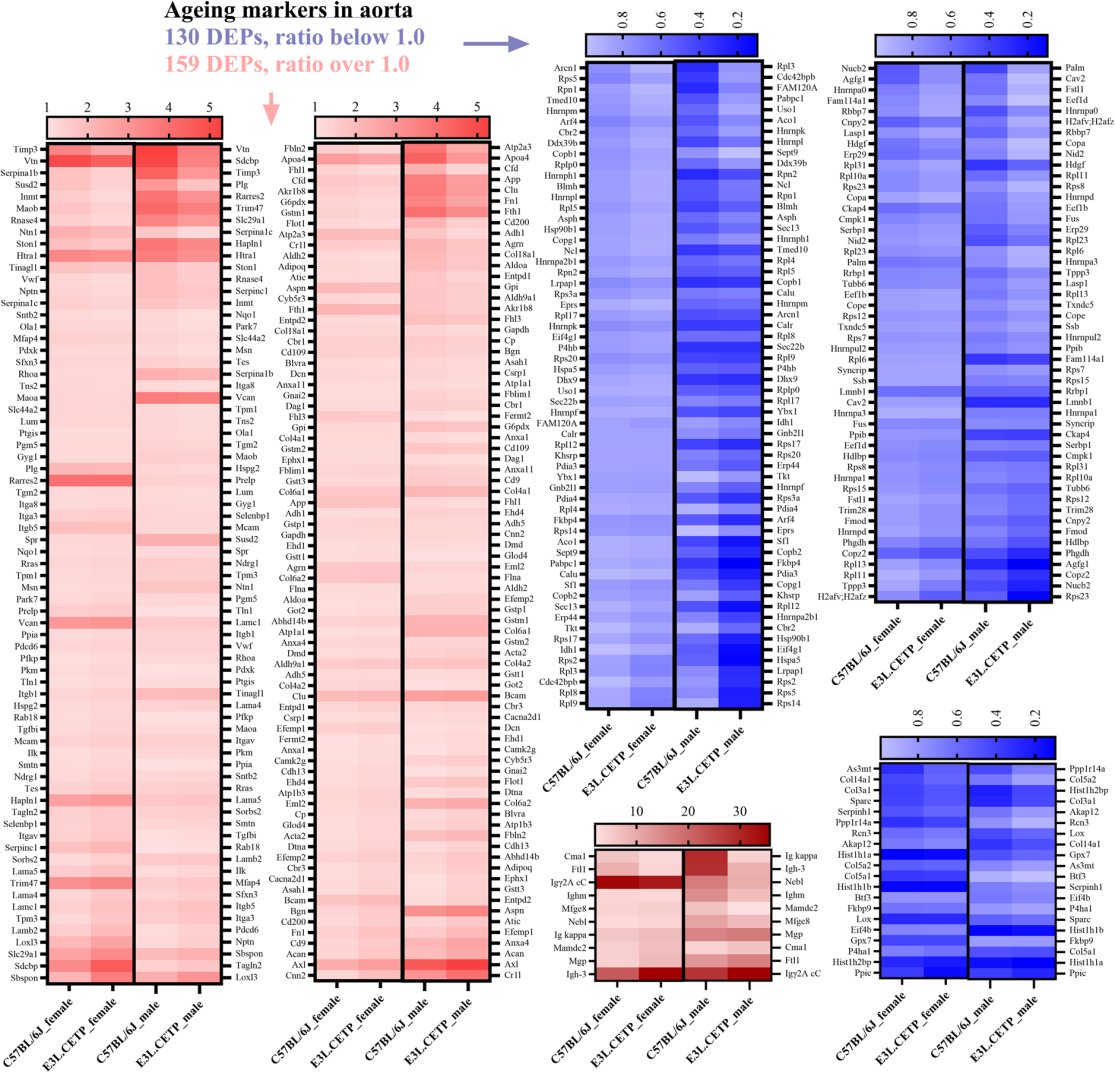
Ageing markers in murine aorta independent of strain and sex of mice. Heatmaps of ratio of ageing (40-week-old vs 8-week-old) markers expression in murine aorta of 159 upregulated (ratio ≥ 1.0) as well as 130 downregulated (ratio < 1.0) differentially expressed proteins (DEPs) in E3L.CETP and C57BL/6J male and female mice. Statistics: Student’s *t*-test. Presented heatmaps were prepared based on proteins that were significantly different between compared groups

To verify whether specific ageing markers in aortic proteome would indicate differences in 40-week-old E3L.CETP vs C57BL/6J mice, reflecting differences in endothelial function, more specific analysis was performed. Initially, within 40-week-old vs 8-week-old E3L.CETP or C57BL/6J mice, DEPs were divided based on the ratio (R): above 1.0 (upregulated, Fig. S7 A, C) and below 1.0 (downregulated, Fig. S7 B, D). Then, separately for female and male, unique DEPs for C57BL/6J and E3L.CETP mice were compared and common DEPs independent of strain but specific for female (Fig. S7A: 49 upregulated and Fig. S7B: 78 downregulated) and male (Fig. S7A: 43 upregulated and Fig. S7B: 33 downregulated) mice were identified and presented as heatmaps (Fig. S8). However, performed analysis, based on the identification of ageing markers independent of strain but specific for sex of mice, indicated similar pattern of changes in the aortic proteome of C57BL/6J and E3L.CETP mice. Therefore, common DEPs for female and male C57BL/6J mice as well as common DEPs for female and male E3L.CETP mice were compared in order to obtain common upregulated (Fig. S7C: 159) and downregulated (Fig. S7D: 130) DEPs independent of strain and sex of mice, which expression was also presented on heatmaps (Fig. 6). Surprisingly, almost all DEPs, independent of strain and sex of mice, had similar R of changes in E3L.CETP and C57BL/6J mice, including various DEPs ascribed to changes in extracellular cell matrix regulation (e.g. collagen type VI alpha 1 chain—Col6a1, von Willebrand factor—Vwf, Integrin alpha 8—Itga8, collagen type IV alpha 1 chain—Col4a1, collagen type IV alpha 2 chain—Col4a2, dystroglycan—Dag1, agrin—Agrn). However, some among the identified DEPs were more pronounced in E3L.CETP, in comparison to C57BL/6J mice. In particular, based on KEGG analysis (Fig. S9), the highest fold enrichment was found in upregulated DEPs associated with the extracellular cell matrix regulation (e.g. calponin 2 -Cnn2, AXL receptor tyrosine kinase—Axl, aggrecan—Acan, annexin A4—Anxa4, laminin, alpha 4—Lama4, collagen type VI alpha 2 chain—Col6a2, integrin alpha V—Itgav, Laminin, beta 2—Lamb2), while downregulated DEPs were annotated to ribosomes (e.g. ribosomal proteins: L8—Rpl8 S5—Rps5, S14—Rps14, S3A1—Rps3a1, L12—Rpl12, S2—Rps2, L9 -Rpl9), pointing out that decline in vascular function, in ageing process, in murine aorta of E3L.CETP mice, was related to changes in extracellular matrix and inhibition in protein synthesis.

In order to find hyperlipidaemia markers in aortic proteome, a similar approach as for the identification of ageing markers was adopted, however based on comparison of E3L.CETP to C57BL/6J mice. This approach enabled to identify hyperlipidaemia markers specific for sex but independent of age of mice (Fig. S10A–B) as well as markers independent of sex and age of mice (Fig. S10C–D). Importantly, the number of detected hyperlipidaemia markers specific for sex but independent of age was definitely lower than ageing markers and had similar expression value in 8- and 40-week-old mice (Fig. S11). In contrast to ageing markers, hyperlipidaemia markers independent of sex and age were not found (except one DEP: Apolipoprotein A1 – Apoa1). These results pointed out firstly to the fact that the effect of hyperlipidaemia on vascular wall was less pronounced than that of ageing and secondly indicated that the effect of hyperlipidaemia was sex-dependent, but had a similar influence on vascular proteome in young and old mice.

### *Alterations in plasma proteome in 8-week-old and 28-week-old E3L.CETP male and female mice as compared to age and sex-matched C57BL/6Jby globalproteomic analysis*

To correlate vascular phenotype of accelerated endothelial dysfunction development in E3L.CETP mice with plasma proteome, global proteomic analysis in plasma was performed in 8-week-old and 28-week-old male and female E3L.CETP mice as compared to age and sex-matched C57BL/6J mice. To compare the plasma proteome of E3L.CETP and C57BL/6J mice, the 28-week-old were used, being at the stage of clear difference in endothelial function in vivo. The ageing and hyperlipidaemia markers in plasma were identified using a similar approach as for ageing and hyperlipidaemia markers in the aorta (Fig. S4).

**Fig. 7:**
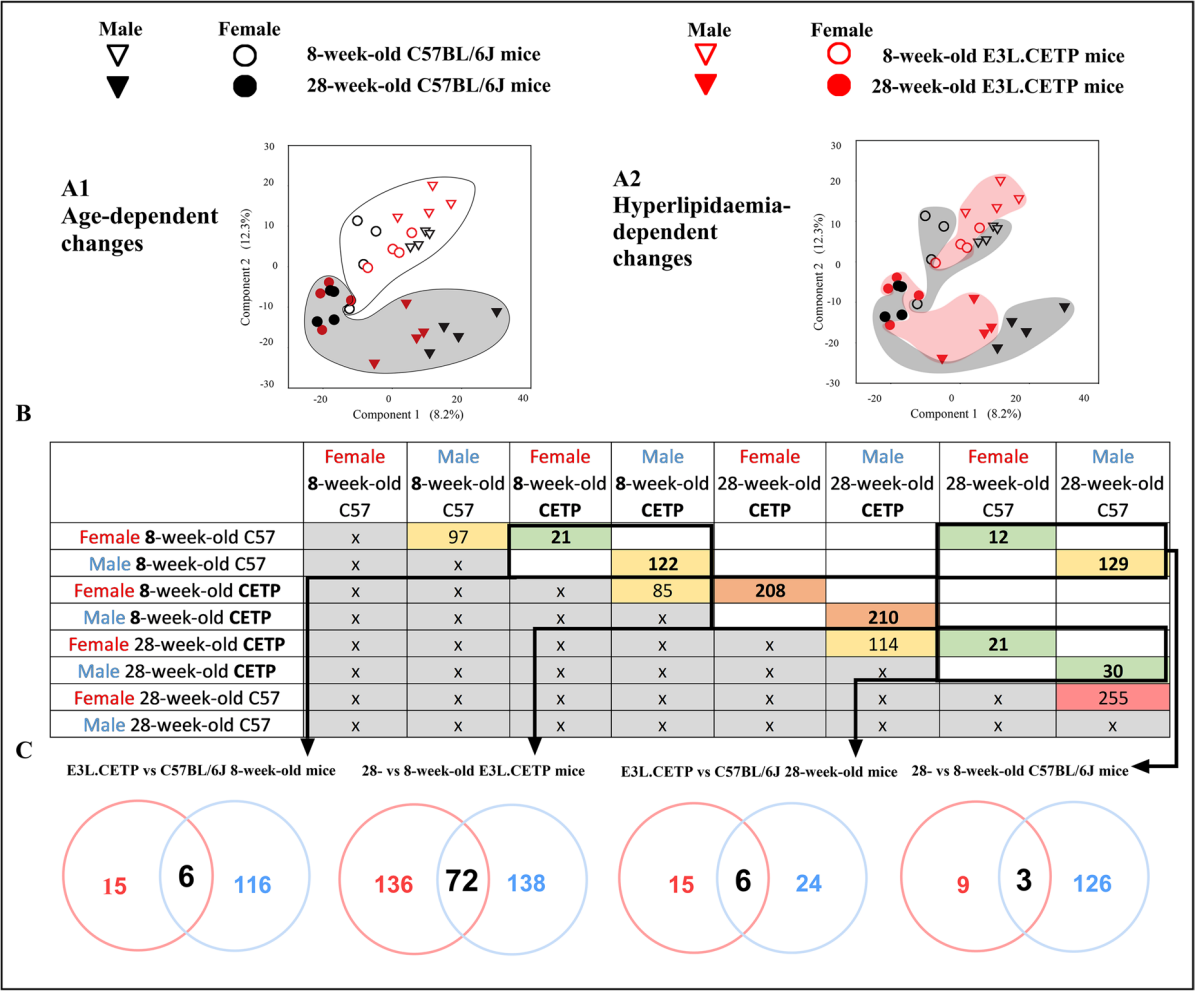
Summary of significant changes in plasma proteome revealed by global proteomic analysis in plasma. Principal component analysis of protein content variance in plasma (**A1**, **2**, PCA). Table (**B**) and Venn diagrams (**C**) presenting differentially expressed proteins (DEPs) in plasma in 8- and 28-week-old female and male E3L.CETP (CETP) mice in comparison to age- and sex-matched control mice C57BL/6J (C57) as well as in ageing process in the female and male E3L.CETP and C57BL/6J mice. Size of groups: *n* = 4. Statistics: Student’s *t*-test. Legend: red circles represent DEPs for female and blue circles for male mice. Presented PCA plots, table and Venn diagrams were prepared based only on proteins that were significantly different between compared groups

Multidirectional proteomic analyses were performed to analyse ageing-related changes in plasma (8-week-old mice compared to 28-week-old mice within the same strain of mice) and hyperlipidaemia effects in 8- and 28-week-old mice (comparison of E3L.CETP mice to age-matched control C57BL/6J mice). Analyses were performed separately in male and female mice. The PCA analysis revealed 1328 statistically significant changes in the concentrations of proteins at 5% FDR across groups of animals that were distinct in sex, lipidaemia status and age. A similar phenomenon as in the aorta was observed, namely more pronounced separation of 8-week-old mice proteome (Fig. 7A1), from proteome of 28-week-old mice than the separation of proteome of E3L.CETP mice vs C57BL/6J mice (Fig. 7A2), in either 8-week-old or 28-week-old mice. Furthermore, the number of identified DEPs in plasma proteome also reflected greater impact of ageing in comparison to hyperlipidaemia on plasma proteome (Fig. 7B–C), and this pattern was also presented in STRING-based analysis (Fig. S12). Interestingly, in contrast to aortic proteome analysis, the number of DEPs involved in ageing process in E3L.CETP mice was greater than in C57BL/6J mice, especially in male mice (208 and 210 for female and male E3L.CETP mice, respectively, as well as 12 and 129 for female and male C57BL/6J mice, respectively). These results suggested that accelerated deterioration of vascular function in ageing, in E3L.CETP vs C57BL/6J mice, detected in vivo by MRI, might have been better reflected by DEPs in plasma than in the aorta. Moreover, accelerated development of endothelial dysfunction in E3L.CETP male mice in comparison to E3L.CETP female mice, detected in vivo, was reflected by a higher number of DEPs (also presented in STRING-based analysis) in comparison between 8-week-old E3L.CETP and C57BL/6J male mice as compared with female mice (122 vs 21, respectively). However, the number of DEPs identified in 28-week-old E3L.CETP vs C57BL/6J mice in both sexes was comparable (30 vs 21, respectively). These early detected changes in the plasma proteome of E3L.CETP male mice reflected pronounced vascular dysfunction in E3L.CETP male mice in comparison to C57BL/6J mice and E3L.CETP female mice.

As regard ageing markers, some DEPs independent of strain but specific for female (Fig. S13A 2 upregulated and Fig. S13B 3 downregulated) and male (Fig. S13A 19 upregulated and Fig. S13B 21 downregulated) mice were detected in plasma and presented as heatmaps (Fig. S14). Despite that most of ageing markers independent of strain but specific for sex of mice indicated similar pattern of changes in the plasma proteome of C57BL/6J and E3L.CETP mice, there was couple of DEPs in E3L.CETP vs C57BL/6J mice, detected only in male but not female: upregulated (serum amyloid A1 -Saa1, R: 400.83 vs 291.00; haptoglobin—Hp, R: 82.94 vs 41.11; orosomucoid 2—Orm2, R: 176.01 vs 143.29, in E3L.CETP male mice vs C57BL/6J male mice, respectively) or downregulated (biliverdin reductase B—Blvrb, R: 0.19 vs 0.35; glutathione peroxidase 1—Gpx1, R: 0.12 vs 0.24; haemoglobin subunit alpha—Hba, R: 0.30 vs 0.38; catalase—Cat, R: 0.19 vs 0.26; in E3L.CETP male mice vs C57BL/6J male mice, respectively). Based on detected DEPs, it was suggested that accelerated age-dependent endothelial dysfunction development in E3L.CETP mice (also more accelerated in male vs female E3L.CETP mice) could be related to increased inflammation and weakening of antioxidant defence in plasma. In turn, among DEPs independent for sex and strain, only one upregulated (immunoglobulin heavy constant gamma 2C—Ighg2c) and only one downregulated (periostin—Postn) DEPs were detected, as presented in a heatmaps (Fig. S13C–D). However, both of detected markers had a higher R of changes in E3L.CETP male and female mice (Ighg2c, R: 8.19 and 8.84 for C57BL/6J female and male mice, respectively; 13.12 and 18.11 for E3L.CETP female and male mice, respectively; Postn, R: 0.37 and 0.62 for C57BL/6J female and male mice, respectively; 0.31 and 0.41 for E3L.CETP female and male mice, respectively). Moreover, few hyperlipidaemia markers independent of age but specific for male and female mice were also detected (Fig. S15A–B), but with a similar pattern of changes in 8- and 28-week-old mice (Fig. S16). There were also only one upregulated (heat shock protein family A—Hspa5) and only one downregulated (apolipoprotein A1—Apoa1) hyperlipidaemia-dependent DEPs independent of strain and sex of mice (Fig. S15C–D) with a similar pattern of changes in 8- and 28-week-old mice. Taken together, analysis of age-related DEPs independent of strain but specific for male and female mice revealed only few processes that could be associated with accelerated endothelial dysfunction development in E3L.CETP mice. Therefore, analysis of age-related DEPs dependent on strain, specific for male and female mice, was performed.

Interestingly, during ageing in plasma of E3L.CETP male mice (Fig. S17) in comparison to C57BL/6J male mice (Fig. S18), a higher number of DEPs were significantly involved, again in response to oxidative stress, observed as a weakening of antioxidative defence (e.g. decreased R of peroxiredoxin-6—Prdx6, peroxiredoxin-2—Prdx2, peroxiredoxin-1—Prdx1, catalase—Cat, glutamate-cysteine ligase—Gclm, solute carrier family 4 member 1—Slc4a1, aminolevulinate dehydratase—Alad) and as inflammatory and oxidative stress markers (e.g. increased R of lipocalin-2—Lcn2 and haptaglobin—Hp) and regulation of metabolic pathways (downregulation of DEPs in E3L.CETP mice vs upregulation in C57BL/6J mice), suggesting that systemic inflammation, oxidative stress and alterations in vascular metabolism could be associated with accelerated vascular ageing, detected in vivo in E3L.CETP male mice.

Then, analysis of age-dependent, hyperlipidaemia-related DEPs for male and female mice was performed to find out whether any of the above processes could be linked to the response to hyperlipidaemia. In 8-week-old mice, some of DEPs were in fact involved in the response to oxidative stress, but contrary to the DEPs involved in ageing in E3L.CETP mice, the response to oxidative stress was observed as activation of antioxidative defence (e.g. increased peroxiredoxin-6—Prdx6, peroxiredoxin-2—Prdx2, catalase—Cat, glutamate-cysteine ligase—Gclm, solute carrier family 4 member 1—Slc4a1, aminolevulinate dehydratase—Alad). The regulation of metabolic pathways (contrary to DEPs involved in ageing in E3L.CETP mice—upregulation of DEPs) as well as inflammation were also the dominant processes, in which hyperlipidaemia-dependent (E3L.CETP vs C57BL/6J mice) DEPs were involved, in plasma of 8-week-old male mice. Simultaneously, these processes were not detected in 8-week-old female mice (Fig. S19), what could reflect accelerated vascular ageing in young E3L.CETP male vs female mice. In 28-week-old mice, hyperlipidaemia-dependent changes were comparable in both sexes (Fig. S20).

**Fig. 8:**
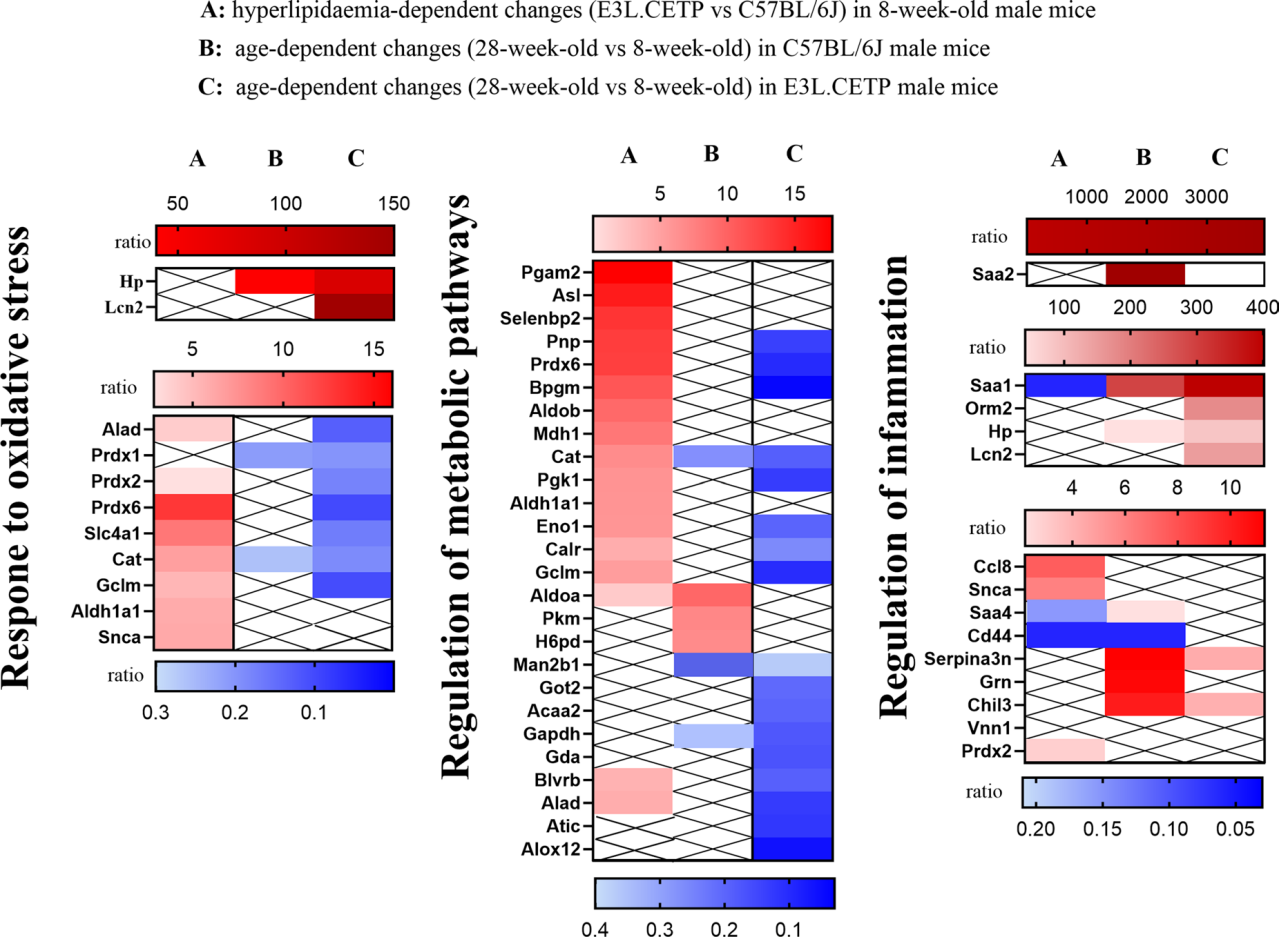
Early hyperlipidaemia-dependent changes in male mice, that might have contributed to accelerated age-dependent development of changes in plasma proteome in E3L.CETP male mice. Characterisation of the ageing-induced changes in plasma of E3L.CETP male mice vs C57BL/6J male mice, of those biomarkers that appeared dominant in the early stage of the hyperlipidemia in plasma of 8-week-old mice, based on heatmaps of expression ratio of hyperlipidaemia (E3L.CETP vs C57BL/6J) markers dependent on age and specific for sex in murine plasma of 8-week-old male mice (**A**) markers of ageing (28-week-old vs 8-week-old) dependent on age and specific for sex in murine plasma in C57BL/6J (**B**) and E3L.CETP (**C**) mice, involved in response to oxidative stress, regulation of metabolic pathways and regulation of inflammation. Red colour indicates upregulated (ratio ≥ 1.0) while blue colour indicates downregulated (ratio < 1.0) differentially expressed proteins (DEPs). Crossed out white box indicates absence of DEPs in the considered comparison. Presented heatmaps were prepared based on proteins that were significantly different between compared groups

These results might suggest that hyperlipidaemia effects in plasma, detected in 8-week-old mice, had an impact on the profile of accelerated vascular ageing development in E3L.CETP mice (Fig. 8). In particular, in E3L.CETP male mice, a more activated response to oxidative stress and more activated regulation of metabolic pathways, with a moderate inflammation (presence of upregulated and downregulated DEPs) in 8-week-old E3L.CETP vs C57BL/6J mice (presented in column A in Fig. 8), changed into weakness of oxidative defence, downregulation of DEPs involved in regulation of metabolic pathway and to increased inflammation during ageing, that was definitely more advanced in E3L.CETP (presented in column C in Fig. 8) vs C57BL/6J (presented in column B in Fig. 8) mice. All these changes identified in young E3L.CETP male mice might have in consequence led to accelerated vascular ageing in E3L.CETP mice in comparison to C57BL/6J mice but also in comparison to E3L.CETP female mice.

### *Alterations in vascular mitochondrial metabolism in 48-week-old E3L.CETP mice*

To verify whether vascular metabolism could indeed differentiate functional vascular phenotype in old male vs female E3L.CETP mice, as results of plasma proteomic suggested, the MST was performed in aortic rings ex vivo, isolated from 48-week-old E3L.CETP mice and age- and gender-matched C57BL/6J mice, using Seahorse XFe96 Analyzer, incubated for 24 h with IL-β. Basal OCR, which reflects mitochondrial respiration was significantly impaired in male E3L.CETP mice as compared to C57BL/6J mice, as well as ATP production and proton leak (Fig. S21). In female E3L.CETP mice, no changes were observed in any of the bioenergetic parameters measured.

## Discussion

In the present work, we demonstrated that life-long, mild hyperlipidaemia in E3L.CETP mice fed chow diet, in a pre-atherosclerosis stage, accelerated age-dependent impairment of the endothelial function. This phenomenon was more advanced in male mice as compared with female mice and was better reflected by proteomic changes in plasma than in the aorta. Interestingly, we identified that early resilience in young female E3L.CETP mice to develop impaired vascular vasodilator function was associated with a switch from NO-dependent to H_2_O_2_-dependent vasodilation, blunted systemic proteomic response in plasma and then slower age-dependent deterioration of endothelial dysfunction in female versus male E3L.CETP mice. Altogether, our results indicate that early resilience to the detrimental effects of hyperlipidaemia on vascular function at the young age may determine the slower progression of age-dependent vascular dysfunction that was featured by vascular inflammation, oxidative stress and alterations in vascular metabolism.

It was a surprising finding that in 40-week-old E3L.CETP mice and C57BL/6J mice, the aortic proteome did not uncover substantial differences. In fact, we found that changes related to vascular ageing were in major part similar in 40-week-old E3L.CETP mice and in 40-week-old C57BL/6J mice. Furthermore, accelerated age-dependent endothelial dysfunction development in E3L.CETP mice was not reflected by consistent changes in endothelial-specific protein biomarkers. However, we found clear-cut hyperlipidaemia-induced changes in plasma proteome with a sex-specific profile of DEPs in E3L.CETP mice vs control mice. In particular, young male E3L.CETP mice exhibited more pronounced changes in plasma proteomic profile which reflected lesser resilience to detrimental effects of hyperlipidaemia on endothelial function as compared to female E3L.CETP mice. These results suggest that the pattern of early vascular response might be a key factor contributing to vascular ageing and could be reflected in the plasma proteome at the young age.

There is general consensus in the literature that early identification of subclinical atherosclerosis is instrumental to shift the paradigm from secondary to effective primary prevention of cardiovascular diseases. Our results support that notion, and add another aspect to this paradigm: the pattern of vascular response to hyperlipidaemia at the young age seemed to determine the progression of age-dependent vascular dysfunction at older age occurring still prior to significant atherosclerosis plaque development. Thus, better understanding of the adaptive and maladaptive vascular response to risk factors in young age is instrumental for identification of the mechanisms of the accelerated vascular ageing phenotype to define plasma-based biomarker(s)-guided early diagnosis of the maladaptive vascular response.

Interestingly, accelerated vascular ageing in the present work was best reflected by functional vascular responses assessing NO-dependent function in vivo. In the present work, endothelial dysfunction in vivo was assessed by MRI-based methodology, the method previously validated in a number of studies characterising endothelial function in vivo in various murine models of cardiovascular disease [29, 30, 32, 50–52]. Of note, this approach proved more sensitive to detect changes in endothelial function as compared to ex vivo assessment of endothelial function in the isolated aorta preparation in some of our previous studies [32, 51]. Here, using an MRI-based approach, we demonstrated that the age-dependent development of endothelial dysfunction was accelerated by humanised dyslipidaemia in E3L.CETP mice and detected based on Ach response earlier in life of E3L.CETP mice (14 and 22 weeks of age in males and females, respectively) as compared with C57BL/6J mice (40 weeks of age). FMD in the FA was also earlier impaired in E3L.CETP mice as compared with C57BL/6J mice but the sensitivity for the early detection of impaired FMD as compared with Ach-response was lower, positioning the former method as less sensitive to assess age-dependent impairment of vascular phenotype in vivo.

Importantly, in the present work, we analysed the age-dependent development of endothelial dysfunction in E3L.CETP mice fed a chow diet resulting in mild hyperlipidaemia, in contrast to high fat and cholesterol-containing diets as used in a number of previous studies in this model [18–23, 26, 27]. We also confirmed the absence of advanced atherosclerotic plaques. Even in the 40-week-old in E3L.CETP mice, there were only subtle early pre-atherosclerotic changes in the aortic roots without atherosclerotic plaques in the BCA. These results were in line with the notion that mild plasma cholesterol elevation was not sufficient to induce robust atherosclerosis plaques in rodents [53–55] without additional insults [56] or a Western diet [57] but develops in response to very high cholesterol levels, e.g. in ApoE/LDLR^−/−^ mice [30]. Importantly, the lack of significant atherosclerosis in 40-week-old E3L.CETP mice confirmed that our results refer to a phase of vascular ageing in hyperlipidaemia, before the advanced atherosclerotic plaque development, with the only presence of early lesions in aortic roots but not in BCA, where the robust atherosclerosis is developing in murine models of atherosclerosis [30]. Importantly, despite the fact that advanced form of atherosclerotic plaque was still not observed even in 16–18-month-old E3L.CETP mice, a sex-related diversity in the intensification of the early plaque development was observed with more intense changes in female as compared with male mice. In fact, in 16–18-month-old E3L.CETP male mice, only slight disorganisation of the elastic lamina was observed, while in 16–18-month-old E3L.CETP female mice neointima formation and presence of foam cells were detected. Our results are in accordance with studies in humans showing that younger women have a decreased tendency to develop cardiovascular diseases to men, including atherosclerosis, but then women catch up to men at the age of 60–79 years and even surpass men by the age of 80 years [58–60]. Yet, it must be emphasised again that these lesions that were found in 16–18-month-old E3L.CETP female mice fed chow diet represent quite an early stage of atherosclerosis development, incomparable with the atherosclerotic plaques found for example in ApoE/LDLR^−/−^ mice fed chow diet [30] or E3L.CETP mice fed Western diet [19, 21].

In this context, E3L.CETP mice fed chow diet represent a unique model of the age-dependent trajectory for the accelerated progression of endothelial dysfunction, induced by mild hyperlipidaemia superimposed on ageing that occurs before atherosclerosis development. Accordingly, the humanised dyslipidaemic E3L.CETP mice represent an early vascular ageing phenotype (EVA) [61] and therefore should provide an unique insight into EVA induced by life-long hyperlipidaemia a predisposing factor for early atherosclerosis development [10, 11]. In our previous studies in ApoE/LDLR^−/−^ mice, it was not possible to dissect the effects of hypercholesterolaemia, atherosclerosis and ageing on the progression of endothelial dysfunction, since complex and multifactorial endothelial dysfunction was detected already at the age of 8 weeks, while atherosclerotic plaques were already present at the age range of 12–14 weeks in ApoE/LDLR^−/−^ mice [30]. Similarly, in most previous studies by others, the effect of hyperlipidaemia was studied in young animals [16] even though vascular ageing may alter the endothelial response to hypercholesterolaemia [17].

The most striking feature of hyperlipidaemia-induced changes found in this work was resilience to detrimental effects of hyperlipidaemia on vascular endothelium-dependent vasodilation in 8-week-old female E3L.CETP mice that was associated with a switch from NO-dependent to H_2_O_2_-dependent vasodilation. Interestingly, this phenomenon was associated with blunted systemic proteomic response in plasma and slower age-dependent deterioration of endothelial dysfunction in female vs male E3L.CETP mice. These results seem to suggest that the pattern of vascular response to hyperlipidaemia at the young age may determine the progression of age-dependent vascular dysfunction.

Although it has been observed in humans that H_2_O_2_ is an endothelium-derived hyperpolarizing factor (EDHF) that contributes to flow-induced dilation in human coronary arterioles from patients with heart disease [62], to the best of our knowledge, here, we showed for the first time a switch from NO-dependent to H_2_O_2_-dependent vasodilation induced by dyslipidaemia in the conduit type of vessel, in the aorta in response to Ach. Furthermore, this response preceded the impairment of the FMD in the FA. In turn, the FMD-induced response in this study was abrogated by L-NAME in vivo in both the C57BL/6J and E3L.CETP mice, indicating that the FMD response was mediated by NO in both strains of mice. In contrast, in the young E3L.CETP mice, the Ach-induced response of the aorta was weakly affected by L-NAME in vivo, until the age of 14 weeks and 22 weeks (the age when endothelial dysfunction appeared) in the male and female mice, respectively. In the aorta, ex vivo catalase inhibited the Ach-induced vasodilation in young E3L.CETP mice, particularly in females but was without effects in older E3L.CETP mice. In addition, the involvement of H_2_O_2_ in the endothelium-dependent response was confirmed by an increased level of H_2_O_2_ production in the aorta. The fivefold higher H_2_O_2_ production in the aorta in the young female E3L.CETP mice in comparison to the male E3L.CETP mice demonstrated a clear-cut sexual dimorphism in this response. Overall, these results suggested that the Ach-induced response was H_2_O_2_-dependent particularly in young female E3L.CETP mice. The mechanisms involved in the switch from NO-dependent to H_2_O_2_-dependent vasodilation in the aorta in E3L.CETP mice needed to be delineated in more detail and could be similar to or different from the mechanism proposed earlier for resistance arteries [63–66].

Nevertheless, results of our study may provide valuable insights on the mechanisms that contribute to the differential manifestations of cardiovascular ageing in males and females, which is currently incompletely understood [67, 68]. Since a switch from NO-dependent to H_2_O_2_-dependent vasodilation was associated with blunted plasma proteomic response and slower endothelial dysfunction development in female vs male E3L.CETP mice, this response might have an adaptive nature. However, as the source of the H_2_O_2_ was not identified in the present study, it could not be excluded that production of H_2_O_2_ might play a pathophysiological role. Interestingly, it was demonstrated that diabetic mice exhibited miR-21-mediated switch in the mediators of coronary endothelium-dependent dilation, from a NO to H_2_O_2_, which contributed to microvascular dysfunction. Therefore, the NO to H_2_O_2_ switch in endothelium-dependent dilation could be a pathological indicator of coronary microvascular dysfunction in diabetes [66]. Further studies are needed to determine the functional role of the switch from NO to H_2_O_2_ in E3L.CETP mice. 

Along with the switch from NO-dependent to H_2_O_2_-dependent vasodilation in the aorta in young female E3L.CETP mice, we identified a number of sex-specific hyperlipidaemia-dependent DEPs in 8-week-old E3L.CETP mice, while in older mice (28-week-old), the number of hyperlipidaemia-dependent DEPs was comparable between sexes. Of note, the analysis of DEPs dependent on age and sex allowed for detection of a number of changes in plasma proteome of 8-week-old male E3L.CETP mice with response to oxidative stress and regulation of metabolic pathways detected as the main two processes. Interestingly, similar processes (and also similar DEPs) featured vascular ageing as discussed above. However, in contrast to ageing, upregulation instead of downregulation of antioxidative defence and metabolic pathway were detected in 8-week-old male E3L.CETP vs C57BL/6J mice. These results suggest that these processes could be activated as protection in an early response to mild hyperlipidaemia but their activity declines with age, leading to accelerated endothelial dysfunction development.

Overall, these findings suggest that the early resilience to detrimental effects of hyperlipidaemia on vascular endothelium-dependent vasodilation in young 8-week-old female E3L.CETP mice represented the important difference in response to hyperlipidaemia as compared with 8-week-old E3L.CETP male mice. This phenomenon was associated with blunted systemic proteomic response in plasma and slower age-dependent deterioration of endothelial dysfunction in female versus male E3L.CETP mice. Accordingly, we suggest that the early pattern of vascular response to hyperlipidaemia at the young age may determine the accelerated ageing in E3L.CETP male mice, detected in the plasma proteome and by MRI in vivo as an accelerated progression of age-dependent endothelial dysfunction. These results are in accordance with the Young Finns Study, which has demonstrated that presence of pathology risk factors in childhood had a significant impact on pathology development in adults [69].

The interesting sex-specific feature of accelerated vascular phenotype in 28-week-old E3L.CETP male mice was the downregulation of metabolic pathways, which was not observed in E3L.CETP female mice or in C57BL/6J mice of both sexes. In particular, there were some downregulated DEPs detected during ageing in male E3L.CETP mice (e.g. peroxiredoxin-6, calreticulin, gamma glutamyl cysteine ligase) deficiency of which were reported to be associated with mitochondrial dysfunction [70–72]. Using a recently optimised protocol for functional analysis of vascular energy metabolism in isolated single rings of the murine aorta, by the Seahorse XFe96 Analyzer [33], we demonstrated that mitochondrial respiration, reflected by basal OCR, was significantly impaired in 48-week-old male but not female E3L.CETP mice as compared to C57BL/6J mice. Measurements were performed in aortic rings after stimulation with IL-β (24 h pre-incubation), since measurements in basal conditions did not allow to reveal significant changes between C57BL/6J and E3L.CETP mice (data not shown). These results indicate that accelerated vascular ageing in male vs female E3L.CETP mice might be indeed associated with an impaired reserve of mitochondrial energy metabolism in E3L.CETP male mice in response to a pro-inflammatory stimulus, as compared with E3L.CETP female mice. In general, these results are in line with some previous studies demonstrating the role of vascular mitochondrial dysfunction in vascular ageing and therapeutic effects of NAD precursors preventing vascular ageing [73–77]. Whether this type of interventions would prevent accelerated age-dependent endothelial dysfunction in E3L.CETP mice remains to be established.

In the present work, despite (1) clearly accelerated and progressive impairment of endothelial function in E3L.CETP mice as compared with C57BL/6J, (2) more pronounced impairment of endothelial function in male as compared to female E3L.CETP mice and (3) more pronounced impairment of vascular metabolic status in E3L.CETP male mice as compared with C57BL/6J male mice, we failed to identify a clear-cut biomarker signatures of dyslipidaemia-induced accelerated vascular dysfunction in aged E3L.CETP animals that would clearly reflect more pronounced age-dependent deterioration of vascular function in E3L.CETP mice, in a sex-dependent manner as compared with C57BL/6J mice.

The comprehensive set of data including classical endothelial biomarkers did not uncover specific sex-dependent biomarker of the vascular phenotype in 28-week-old E3L.CETP mice, despite that in our previous studies, biomarkers of endothelial dysfunction were clearly changed during atherosclerosis development [30], at early and late stages of breast cancer progression [78] and to a lesser extent in mice fed a high-fat diet [51]. Although non-targeted proteomics as well as multidirectional analysis of the proteome of the aorta did also not uncover any specific biomarker, it was demonstrated based on these analyses that ageing rather than hyperlipidemia had a dominant impact on the outcome of the proteome analysis in both aorta and plasma samples in old E3L.CETP mice and C57BL/6J mice.

Ageing markers in the aortic proteome identified based on different DEPs analyses revealed that most of them were independent of strain and sex of mice, and had similar ratios of changes in E3L.CETP and C57BL/6J mice with the exception of upregulated in E3L.CETP vs C57BL/6J mice; DEPs associated with the extracellular cell matrix regulation (e.g. calponin 2, AXL receptor tyrosine kinase, aggrecan, annexin A4, laminin, alpha 4, collagen type VI alpha 2 chain, integrin alpha V, Laminin, beta 2), and downregulated DEPs annotated to ribosomes (e.g. ribosomal proteins: L8, S5, S14, S3A1, L12, S2, L9), suggested that accelerated age-dependent development of endothelial dysfunction in E3L.CETP mice could be related to changes in extracellular matrix and inhibition in protein synthesis. However, the latter changes were sex-independent, thus did not reflect more severe impairment of endothelial function in old E3L.CETP male vs female mice.

In contrast to the aortic proteome, the plasma proteome revealed only one upregulated (immunoglobulin heavy constant gamma 2) and one downregulated (periostin) DEPs independent of strain and sex of mice, suggesting that some of age-related DEPs in plasma could reflect difference in the level of endothelial dysfunction development in male vs female E3L.CETP mice. The detected DEPs independent of strain and sex of mice in plasma were more pronounced in E3L.CETP, in particular male mice, what could be especially important in the context of periostin, being a 13th member of the vitamin K-dependent protein (VKDP) family [79]. In our previous study, we demonstrated that vitamin K_2_-MK-7 improves NO-dependent endothelial function in ApoE/LDLR^−/−^ mice [50]; however, it was not determined whether the beneficial effect of vitamin K_2_-MK-7 was linked to an improved carboxylation status in the endothelium, to matrix Gla protein or to other VKDPs. Therefore, E3L.CETP mice might provide a potentially useful model to study the mechanisms of the vitamin K-dependent effect on endothelial function and vascular ageing independent of atherosclerosis and vascular calcification [80, 81].

On the other hand, even though we identified some ageing markers in the plasma proteome, specific for sex of mice, they were mostly independent of strain of the mice. However, some of them, i.e. upregulated DEPs related to inflammation (serum amyloid A1, haptoglobin, orosomucoid 2) and downregulation of DEPs (biliverdin reductase B, glutathione peroxidase 1, haemoglobin subunit alpha) related to oxidative stress, were identified in E3L.CETP vs C57BL/6J male mice and these processes could have contributed to increased oxidative stress [82–84], and more pronounced endothelial dysfunction in E3L.CETP vs C57BL/6J male mice.

Finally, analysis of processes involved in the regulation of ageing, specific for sex of mice and specific for E3L.CETP and separately for C57BL/6J mice, revealed additional DEPs which deficiency leads to endothelial dysfunction development associated with increased oxidative stress, e.g. decreased antioxidant enzymes: peroxiredoxins—Prdx6, Prdx2, Prdx1, catalase or glutamate-cysteine ligase modifier subunit and delta-aminolevulinic acid dehydratase [85–88]. In addition, there was a higher plasma level of lipocalin 2 during ageing in male E3L.CETP mice, which is a pro-inflammatory adipokine upregulated in obese human subjects and animal models [89].

As a potential limitation of this study, we note that the plasma cholesterol and triglyceride levels remain fairly constant during ageing of the E3L.CETP mice in contrast to humans where they in general rise with age. However, the rise in humans is mostly due to changes in food intake and composition of the diet next to lower physical activity, whereas the mice were maintained on the same chow diet during the entire study. While we acknowledge the limitations associated with our current study, E3L.CETP mice on a chow diet provide an unique tool to study mechanisms of sex-dependent pathogenesis of dyslipidemia-accelerated vascular ageing.

In conclusion, the E3L.CETP mice displayed an accelerated age-dependent impairment of the endothelial function in response to mild hyperlipidaemia as compared with the age-dependent endothelial dysfunction in the C57BL/6J mice. Accordingly, the E3L.CETP mice, a unique, translational murine model of humanised dyslipidaemia [18, 22, 23] when fed a chow diet, displayed an accelerated age-dependent progression of the endothelial dysfunction, preceding atherosclerotic plaque development that might mimic the age-dependent development of the vascular dysfunction trajectory over the life course in humans with mild dyslipidaemia. E3L.CETP mice on a chow diet represent an unique tool to study mechanisms of insidious progression of early adaptive to late maladaptive endothelial responses to humanised dyslipidaemia, with a distinct sex-dependent evolution of changes. In particular, our results suggest that the pattern of vascular response to hyperlipidaemia at the young age may determine the progression of age-dependent vascular dysfunction. Thus, our results support the notion that early identification of detrimental effects of risk factor on endothelial function should be focused in young age to prevent age-related progression of cardiovascular disease. To better understand the relationship between the early endothelial functional phenotype and late alterations in the vascular metabolism, inflammation and oxidant stress, further studies are needed. These types of studies seem instrumental to understand the early mechanisms of adaptive and maladaptive vascular responses for the effective primary prevention of cardiovascular diseases before atherosclerosis development.

## Supplementary Information

Below is the link to the electronic supplementary material.

Supplementary file1 (DOCX 14.0 MB)

## Data Availability

The data, analytic methods and study materials will be made available on reasonable request to other researchers for the purpose of reproducing the results or replicating the procedure. The mass spectrometry data from plasma were deposited to the ProteomeXchange Consortium via the PRIDE partner repository with the dataset identifier PXD029562. Proteomic datasets from the aorta were deposited to the RODBUK Cracow Open Research Data Repository (https://doi.org/10.57903/UJ/PGQTUF).
