# Supplementary material for: Effects of life-long hyperlipidaemia on age-dependent development of endothelial dysfunction in humanised dyslipidaemic mice
Source: GeroScience. 2025 Apr 17;47(3):2673–701. doi: 10.1007/s11357-025-01578-w (PMC12181563; doi:10.1007/s11357-025-01578-w)
Supplement: Supplementary file 1 — Supplementary file1 (DOCX 14.0 MB) [file 11357_2025_1578_MOESM1_ESM.docx]

**Effects of life-long hyperlipidaemia on age-dependent development of endothelial dysfunction in humanised dyslipidaemic mice**

**Anna Bar^1^, Piotr Berkowicz^1^, Anna Kurpinska^1^, Tasnim Mohaissen^1,2^, Agnieszka Karaś^1^, Patrycja Kaczara^1^, Joanna Suraj-Prażmowska^1^, Magdalena Sternak^1^, Brygida Marczyk^1^, Agata Malinowska^4^, Agnieszka Kij^1^, Agnieszka Jasztal^1^, Izabela Czyzynska-Cichon^1^, Elsbet J. Pieterman^5^ , Hans M.G. Princen^5^, Jacek R. Wiśniewski^6^ and Stefan Chlopicki^1,3*^**

^1^ Jagiellonian University, Jagiellonian Centre for Experimental Therapeutics (JCET), Bobrzynskiego 14, 30-348, Krakow, Poland;

^2^ Department of Biomedical Sciences, Faculty of Health and Medical Sciences, University of Copenhagen, Copenhagen, Denmark;

^3^ Jagiellonian University Medical College, Faculty of Medicine, Chair of Pharmacology, Grzegorzecka 16, 31-531, Krakow, Poland;

^4^ Polish Academy of Sciences, Mass Spectrometry Laboratory, Institute of Biochemistry and Biophysics, Pawińskiego St 5a, 02-106 Warsaw, Poland;

^5^ The Netherlands Organisation of Applied Scientific Research (TNO), Metabolic Health Research, Gaubius Laboratory, 2333 CK, Leiden, The Netherlands;

^6^ Max Planck Institute of Biochemistry, Department of Proteomics and Signal Transduction, Martinsried, Germany.

**Journal name:** Geroscience

***Correspondence:**

Prof. Stefan Chlopicki, M.D., PhD.

E-mails: [stefan.chlopicki@jcet.eu](mailto:stefan.chlopicki@jcet.eu)

Jagiellonian Centre for Experimental Therapeutics (JCET),
Jagiellonian University, ul. Bobrzynskiego 14, 30-348 Krakow, Poland.

Telephone number: +48 12 6645464

Fax: +48 12 2974615

**SUPPLEMENTAL MATERIAL**


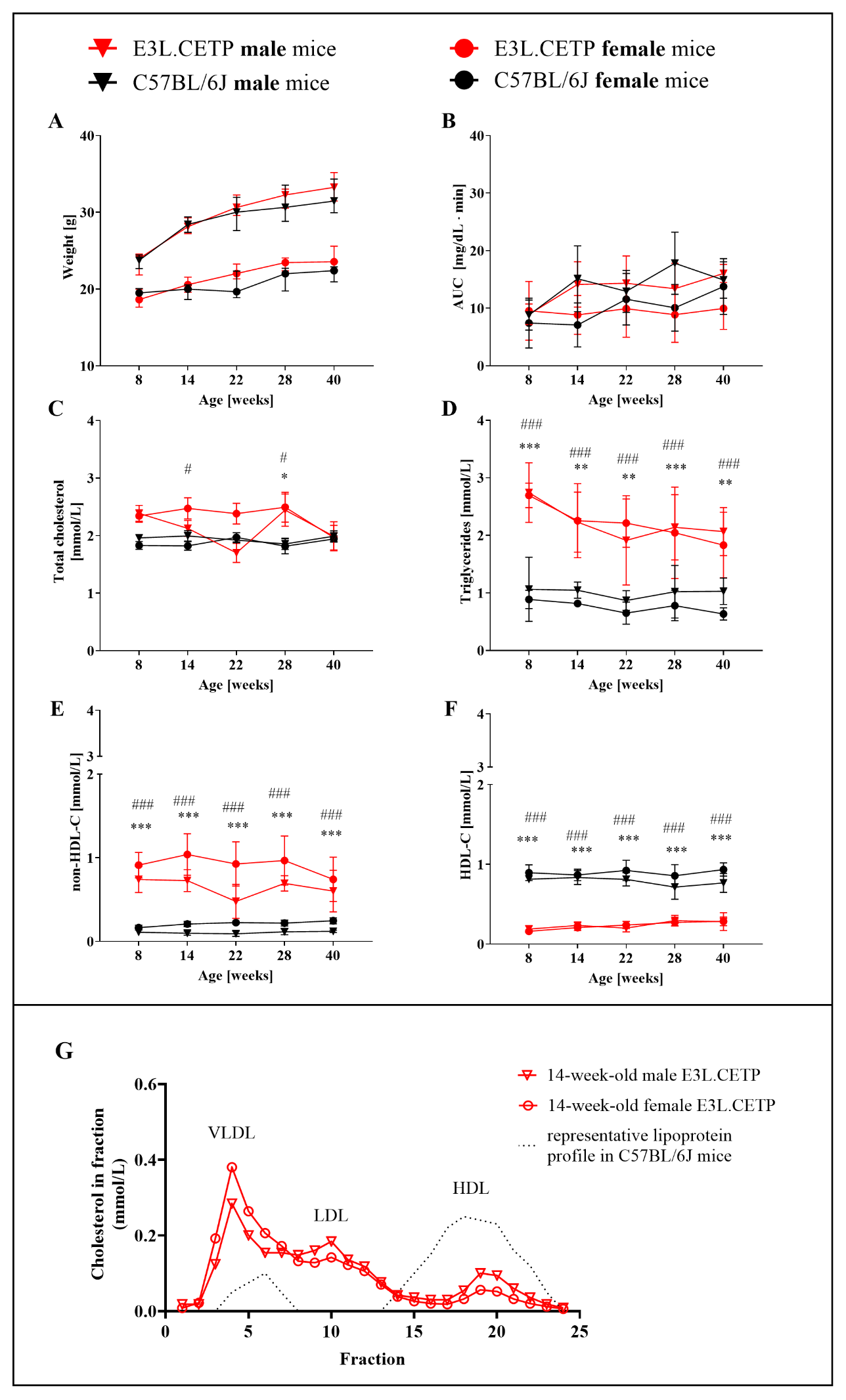


**Figure S1. Weight changes, response to glucose tolerance test and lipid profile in E3L.CETP mice.** Mice weight (**A**), area under the glucose tolerance test (GTT) curve (AUC, **B**), total cholesterol (**C**), triglycerides (**D**), non-high-density lipoprotein cholesterol (non-HDL-C, **E**), HDL-C (**F**) and lipoprotein profiles (**G**) in 8-, 14-, 22-, 28- and 40-week-old E3L.CETP male and female mice, in comparison to age- and gender-matched control mice (C57BL/6J). Size of groups: A: n=16-20, B: n=6-10, C-F: n=5-6. Data are presented as the mean and standard deviation (B-F) or as the median and interquartile range (A). Statistics: B-F: two-way ANOVA followed by Tukey’s post hoc test; A: Kruskal–Wallis ANOVA: * p<0.05, ** p<0.01, *** p<0.001 for E3L.CETP vs C57BL/6J male mice; # p<0.05, ### p<0.001 for E3L.CETP vs C57BL/6J female mice.

**
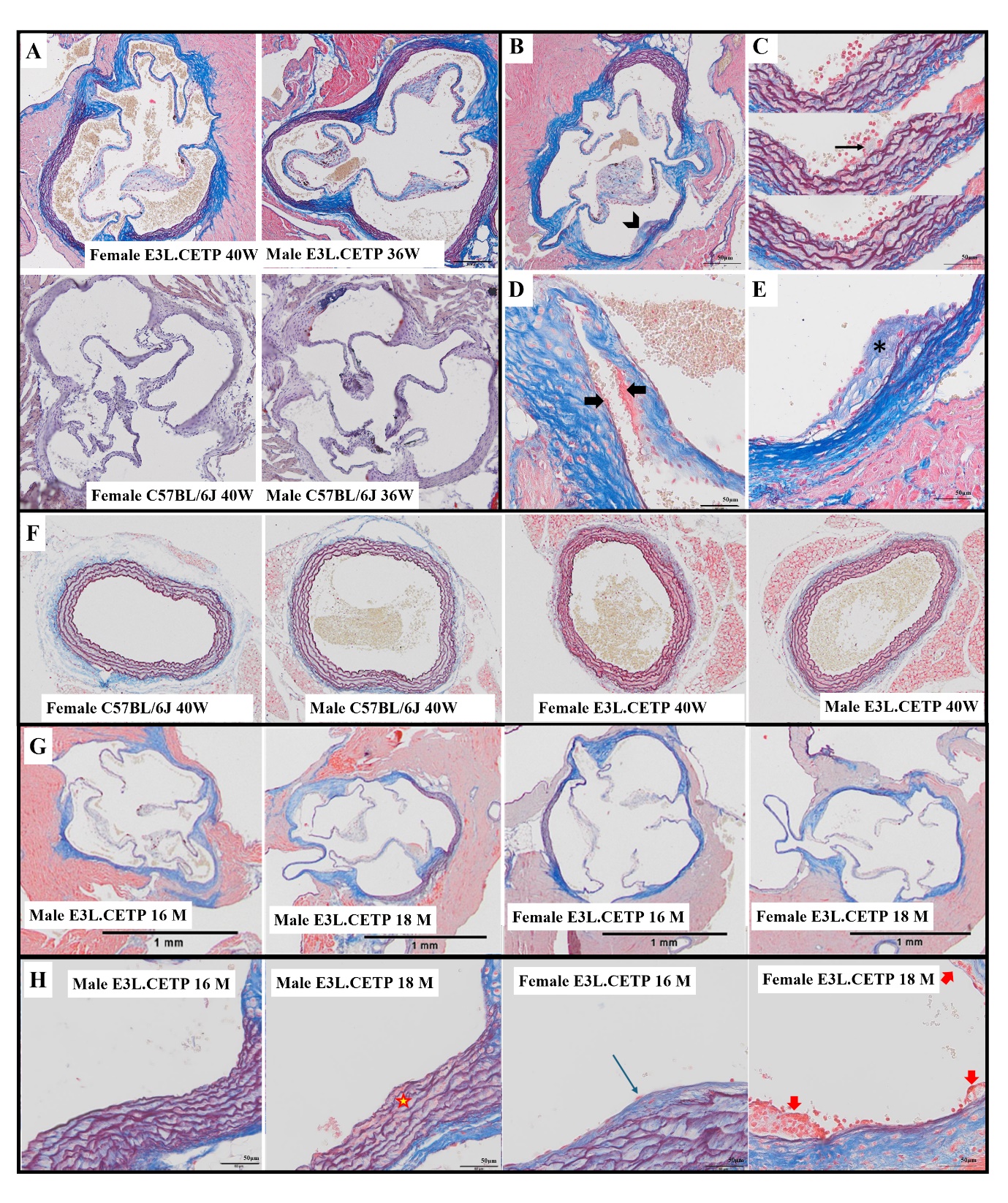
**

**Figure S2. Representative images of the cross-sections of the aortic root and brachiocephalic artery of E3L.CETP mice.** Cross-sections of the aortic root (**A-E**) and brachiocephalic artery (**F**) in 36-40-week-old E3L.CETP vs C57BL/6J (36W, 40W; A-F) male and female mice and of the aortic root in 16-18-month-old E3L.CETP (16M, 18M; **G-H**) male and female mice. **B:** Aortic root with visible early atherosclerotic lesion (black arrowhead). 100x magnification. **C:** Wall of the ascending aorta within the aortic root with visible leukocytes, grouping in the vessel lumen and adherence to the vessel wall. Platelet aggregates were visible between the leukocytes (black arrow). A stack of three sections – with a thickness of 25 μm. 400x magnification. **D:** Aortic root at the level of the annulus with a visible valve leaflet. A layer of fresh fibrin stained red (thick black arrows) was visible on the surface of the annulus and the aortic surface of the valve. 400x magnification. **E:** Organised, collagen-rich plaque on the aortic root surface at the level of the valve annulus (black asterisk). **H:** Wall of the ascending aorta within the aortic root, without significant atherosclerotic changes in 16- and 18-month-old E3L.CETP male mice (only slight disorganization of the elastic lamina was observed, red asterisk) and with neointima formation (blue arrow) as well as formation of the early atherosclerotic plaque with the presence of foam cells (red arrows) in 16- and 18-month-old E3L.CETP female mice. Staining with Unna’s orcein combined with Martius, Scarlet and Blue trichrome (OMSB): A - two upper panels, B, C, D, E, F, G, H; Hematoxylin and eosin staining: A – two lower panels.

**
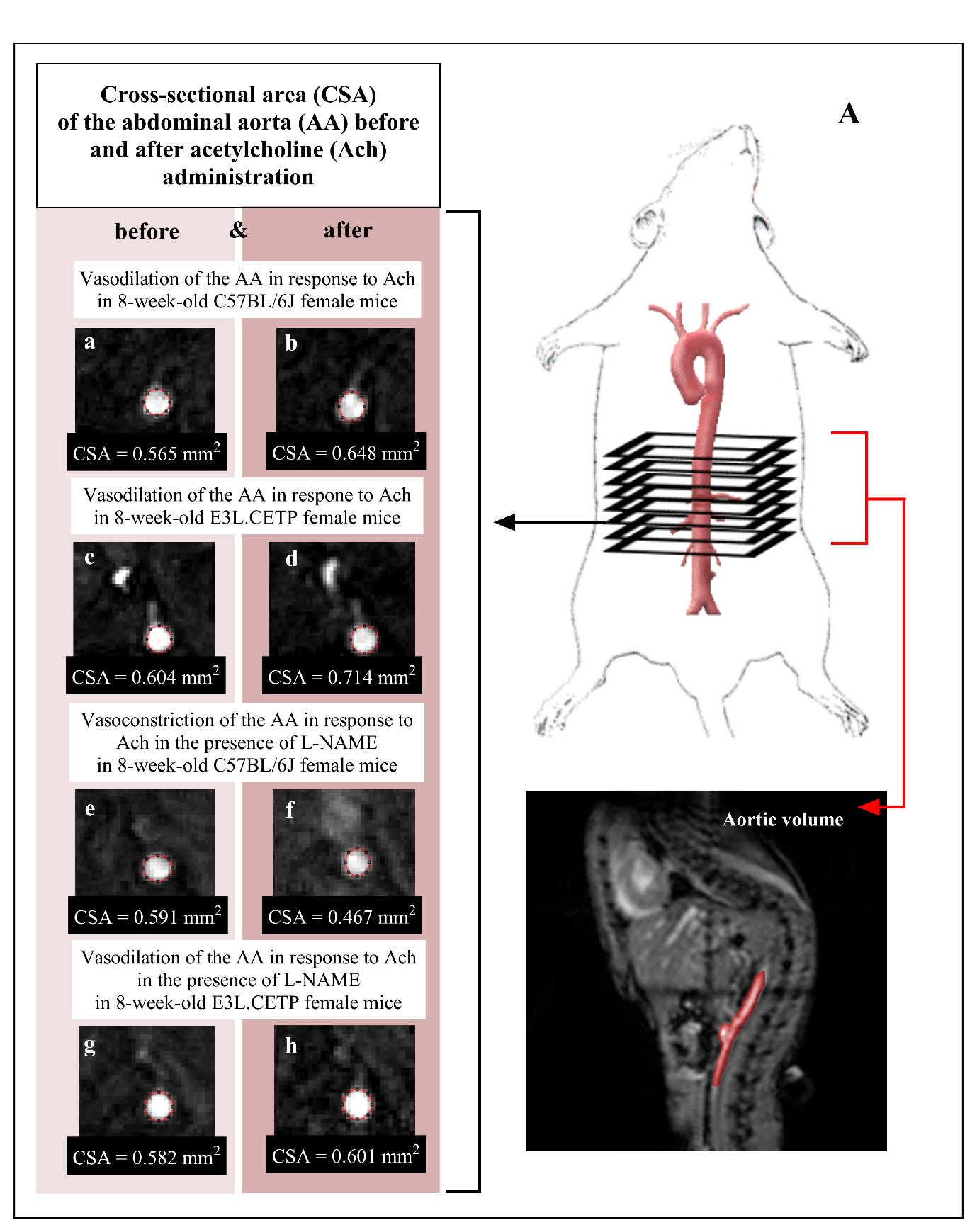
**

**Figure S3. Representative MRI images of the cross-section of the aorta before and after acetylcholine administration.** (**A**) Scheme showing the principle of the MRI–based in vivo detection of endothelium-dependent vascular response induced by acetylcholine (Ach) with representative images of the cross sections of the abdominal aorta (AA) depicting Ach-induced vasodilation in 8-week-old: C57BL/6J (a, b before and after Ach, respectively) and E3L.CETP female mice (c, d before and after Ach, respectively) in the absence of N-nitro-L-arginine methyl ester (L-NAME) as well as in E3L.CETP mice in the presence of L-NAME (g, h before and after Ach, respectively) and Ach-induced vasoconstriction in 8-week-old C57BL/6 female mice in the presence of L-NAME (g,h before and after Ach, respectively). CSA – cross-sectional area.

**Figure S4. Flowchart on methodological pipeline for proteomic studies in the aorta and plasma.** Scheme of data analyses obtained from global quantitative proteomic analysis for aortic and plasma proteome in 8-40-week-old and 8-28-week-old E3L.CETP male and female mice as compared to age and sex-matched C57BL/6J mice, respectively. Multidirectional proteomic analysis was aimed at characterization of global changes as well as the identification of ageing and hyperlipidaemia biomarkers. The figure number containing results of respective data analyses is provided in the square brackets. DEPs - differentially expressed proteins.

**
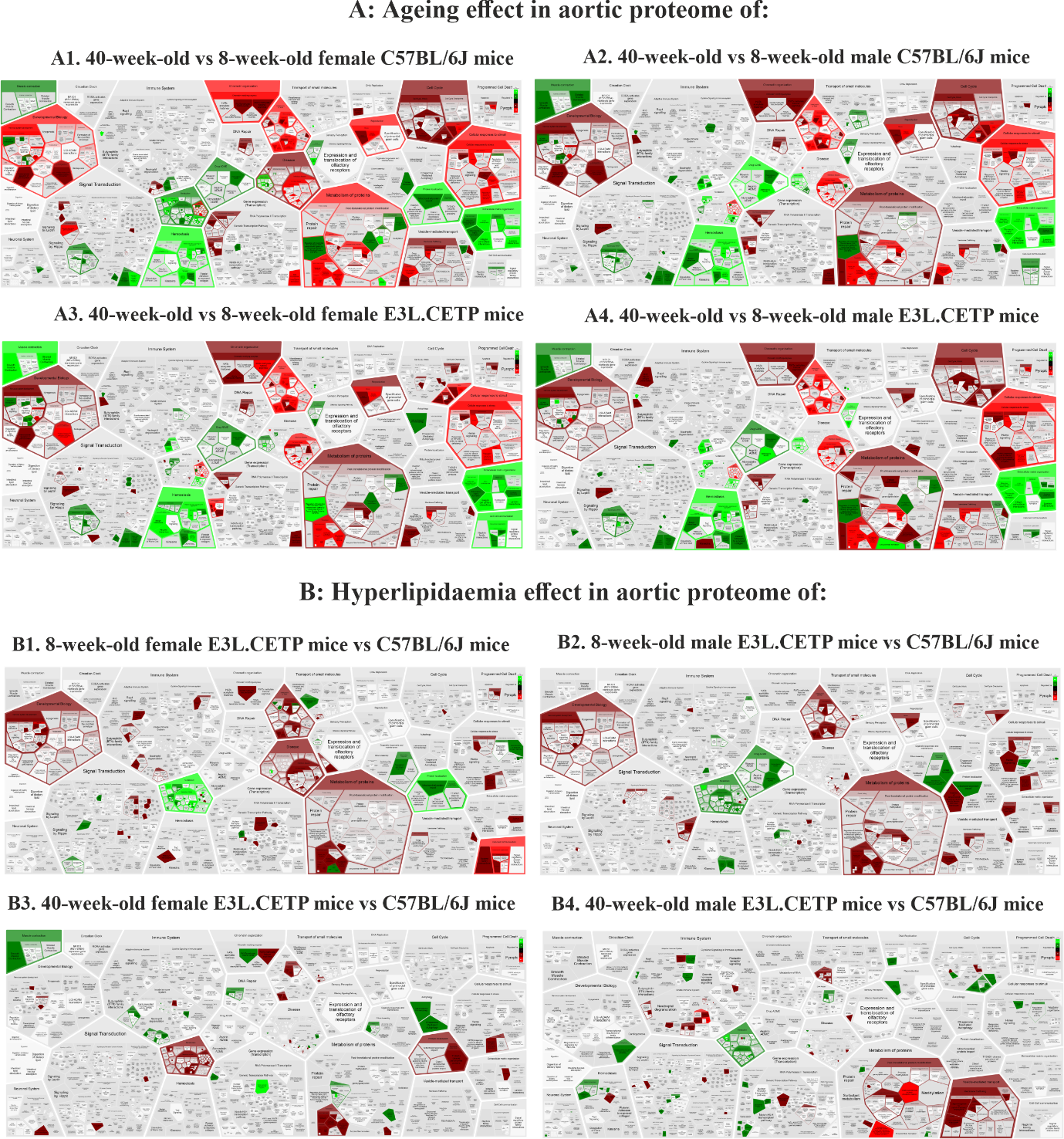
**

**Figure S5. Changes in** **biological processes in the aorta induced by ageing (A) as compared to hyperlipidaemia (B) in E3L.CETP and C57BL/6J mice.** REACTOME diagrams presenting activated and inhibited processes in aorta in 40-week-old vs 8-week-old female (**A1**) and male (**A2**) C57BL/6J mice, in 40-week-old vs 8-week-old female (**A3**) and male (**A4**) E3L.CETP mice, in 8-week-old female (**B1**) and male (**B2**) E3L.CETP mice compared to C57Bl/6J mice and in 40-week-old female (**B3**) and male (**B4**) E3L.CETP mice compared to C57Bl/6J mice. Legend: Increased green intensity indicates greater activation, while increased red intensity indicates stronger inhibition..


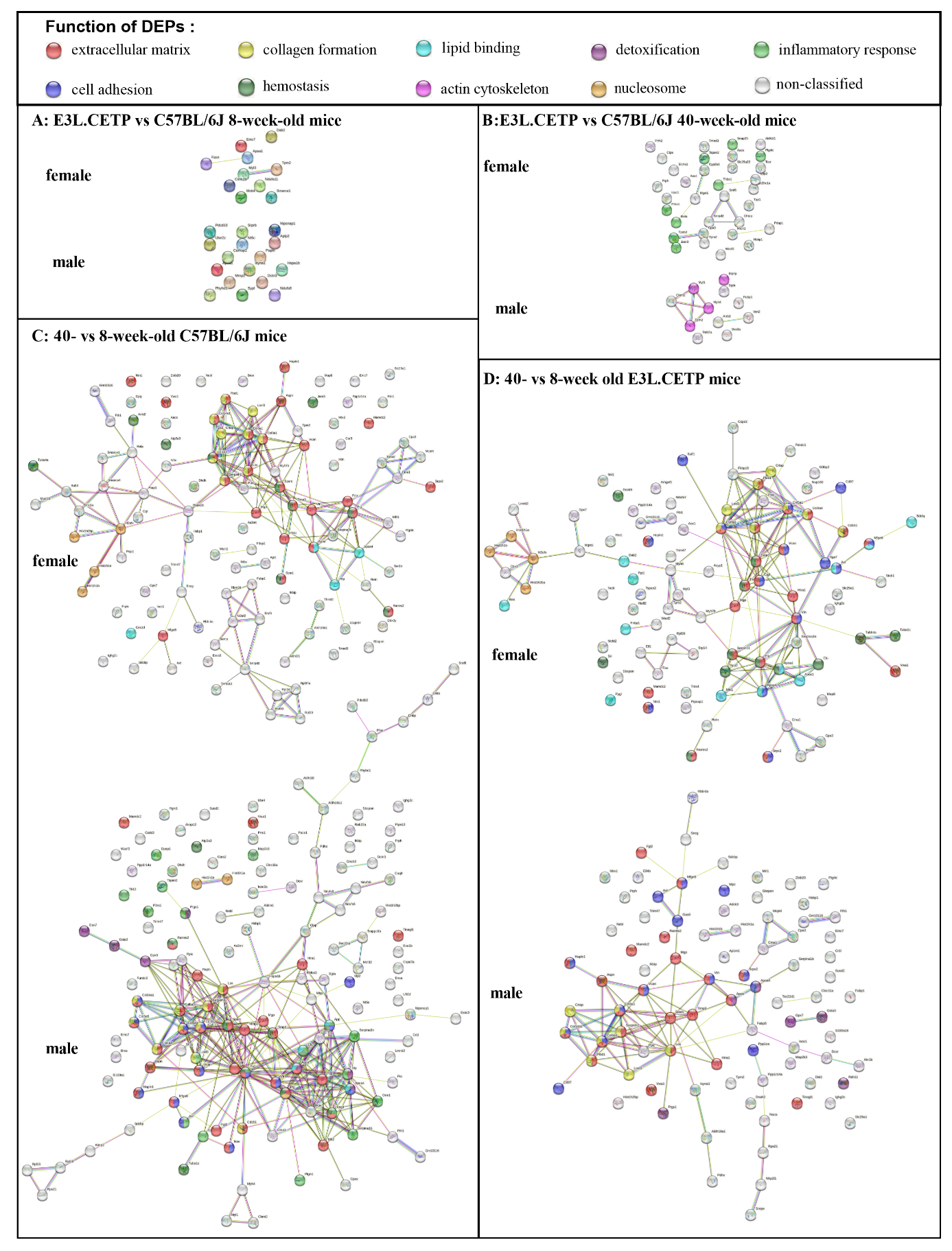


**Figure S6. Differential expressed proteins (DEPs) in aorta detected along ageing in comparison to hyperlipidaemia-dependent DEPs in aortic proteome.** The most significant hyperlipidaemia-dependent proteins differentially expressed (DEPs; 2≤ratio≤0.5) in the male and female 8-week-old (**A**) and 40-week-old (**B**) E3L.CETP vs C57BL/6J mice as well as the most significant age-dependent DEPs in the C57BL/6J (**C**) and E3L.CETP (**D**) 40- vs 8-week-old mice. The DEPs were classified by function as regulation of extracellular matrix (red colour), cell adhesion (dark blue colour), lipid binding (light blue colour), actin cytoskeleton (violet colour), inflammatory response (green colour), collagen formation (yellow colour), hemostasis (dark green colour), detoxification (dark violet colour) and nucleosome (orange colour). The non-classified DEPs are marked as white. Created in STRING: functional protein association networks. The immunoglobulins are not presented in the graphics.


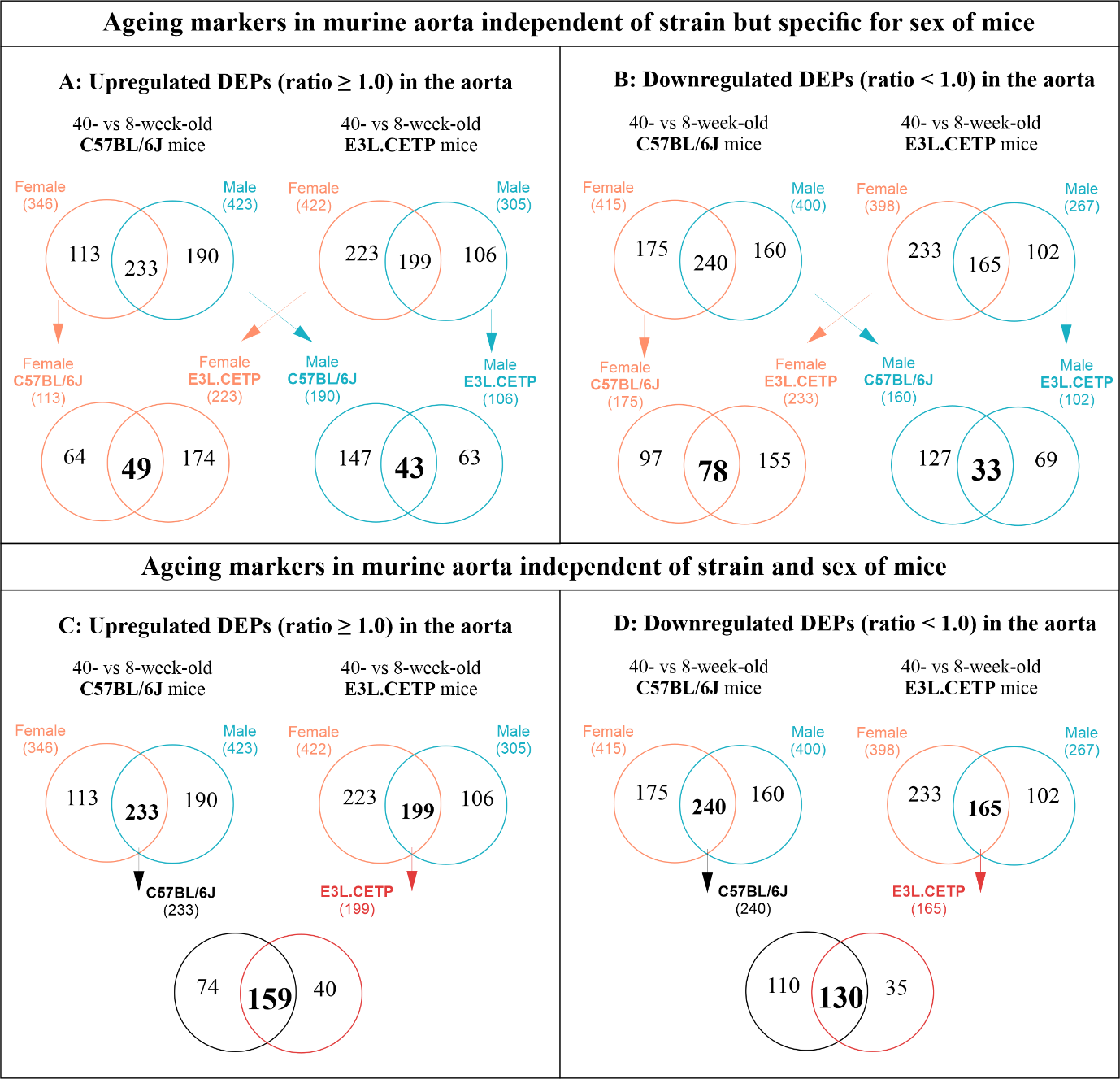


**Figure S7. Ageing markers (respective DEPs) in murine aorta independent of strain but specific for sex of mice as well as independent of strain and sex of mice.** Venn diagrams presenting unique and common DEPs (**A, C**: upregulated ≥ ratio 1.0 or **B, D**: downregulated < ratio 1.0) in aortic proteome between female and male mice in 40- vs 8-week-old E3L.CETP and C57BL/6J mice. From unique DEPs for female C57BL/6J and female E3L.CETP mice as well as from unique DEPs for male C57BL/6J and male E3L.CETP mice, common DEPs independent of strain were identified, specific for female and male, respectively (**A, B**). From common DEPs for female and male C57BL/6J mice as well as from common DEPs for female and male E3L.CETP mice, common DEPs independent of strain and sex of mice were identified (**C, D**).

**Figure S8.** **Ageing markers (respective DEPs) in murine aorta independent of strain but specific for sex of mice.** Heatmaps of ratio of ageing (40-week-old vs 8-week-old) markers expression in murine aorta 43 specific for male (**A**) and 49 specific for female (**B**) upregulated (ratio ≥1.0) as well as 33 specific for male (**C**) and 78 specific for female (**D**) downregulated (ratio < 1.0) differentially expressed proteins (DEPs) in E3L.CETP and C57BL/6J mice. Heatmaps were created based on data presented in Supplementary Tables 8-11 (Table S8-S11).

**Figure S9.** **Biological processes characteristic for ageing in murine aorta**. The biological processes identified based on ageing markers in murine aorta independent of strain and sex of mice with the KEGG database. Upregulated proteins – DEPs with ratio over 1.0; downregulated proteins – DEPs with ratio below 1.0.


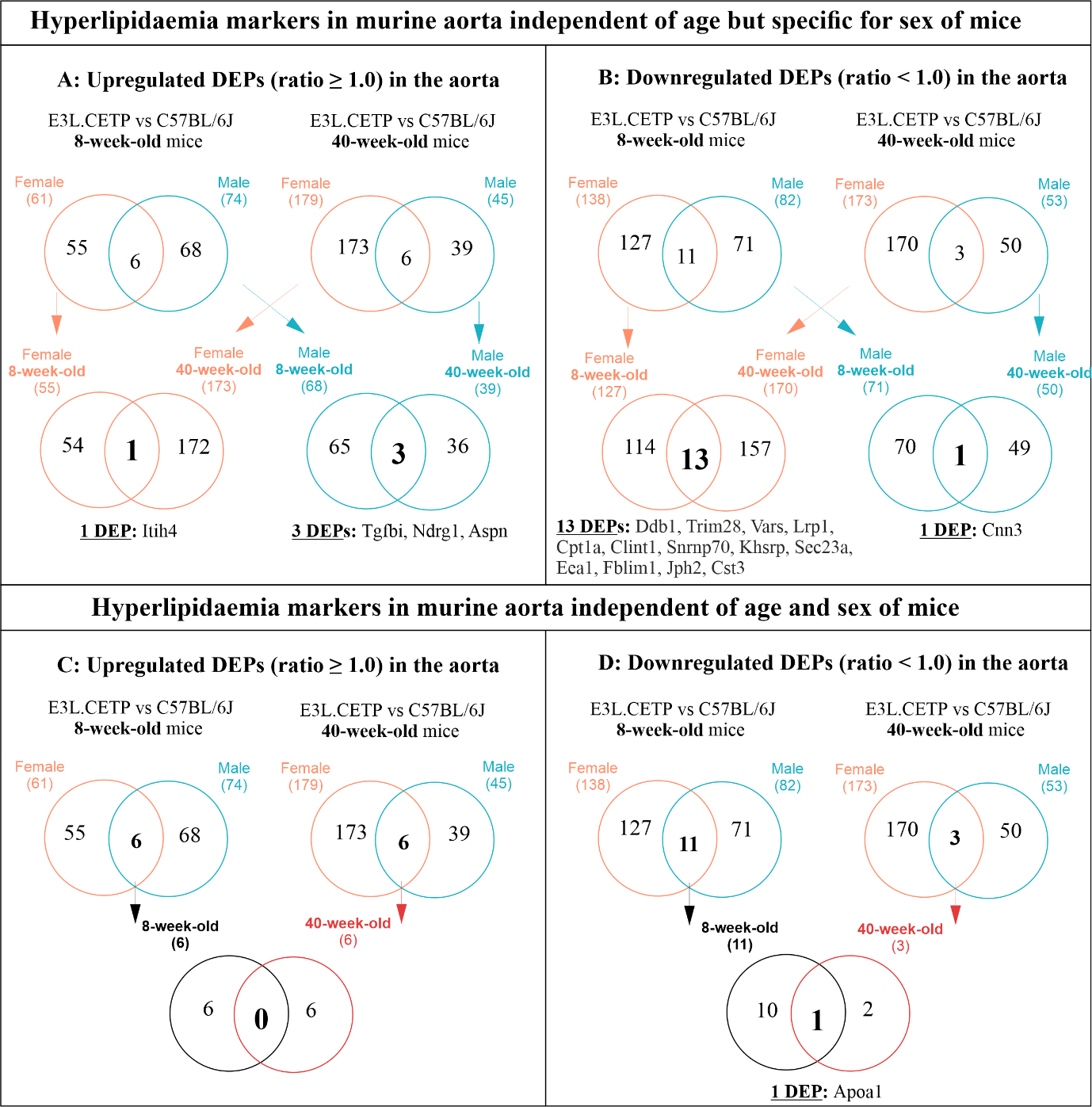


**Figure S10. Hyperlipidaemia markers (respective DEPs) in murine aorta independent of age but specific for sex of mice as well as independent of age and sex of mice.** Venn diagrams presenting unique and common DEPs (**A, C**: upregulated ≥ ratio 1.0 or **B, D**: downregulated < ratio 1.0) in aortic proteome between female and male mice in E3L.CETP vs C57BL/6J 8-week-old and 40-week-old mice. From unique DEPs for female 8-week-old and female 40-week-old mice as well as from unique DEPs for male 8-week-old and male 40-week-old mice, common DEPs independent of age were identified, specific for female and male, respectively (**A, B**). From common DEPs for female and male 8-week-old mice as well as from common DEPs for female and male 40-week-old mice, common DEPs independent of age and sex of mice were identified (**C, D**).


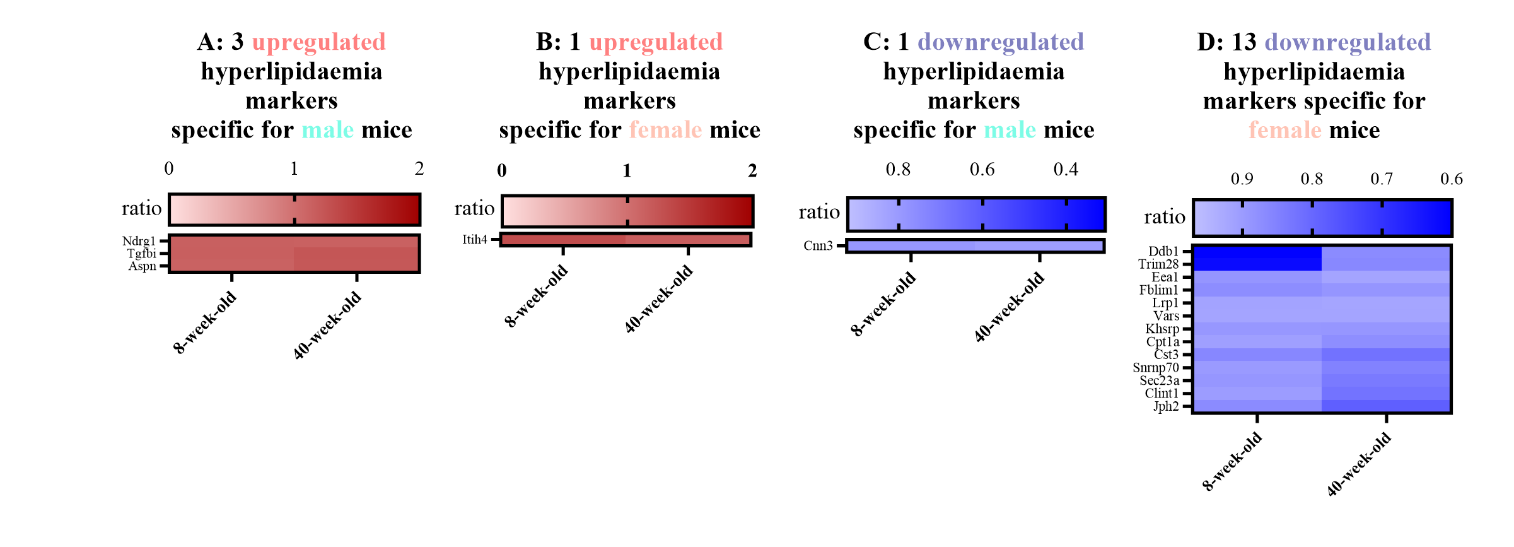


**Figure S11**. **Hyperlipidaemia markers (respective DEPs) in murine aorta independent of age but specific for sex of mice.**

Heatmaps of ratio of hyperlipidaemia (E3L.CETP vs C57BL/6J) markers expression in murine aorta of 3 specific for male (**A**) and 1 specific for female (**B**) upregulated (ratio ≥1.0) as well as 1 specific for male (**C**) and 13 specific for female (**D**) downregulated (ratio < 1.0) differentially expressed proteins (DEPs) in 8-week-old and 40-week-old mice. Heatmaps were created based on data presented in Supplementary Tables 12-15 (Table S12-S15).


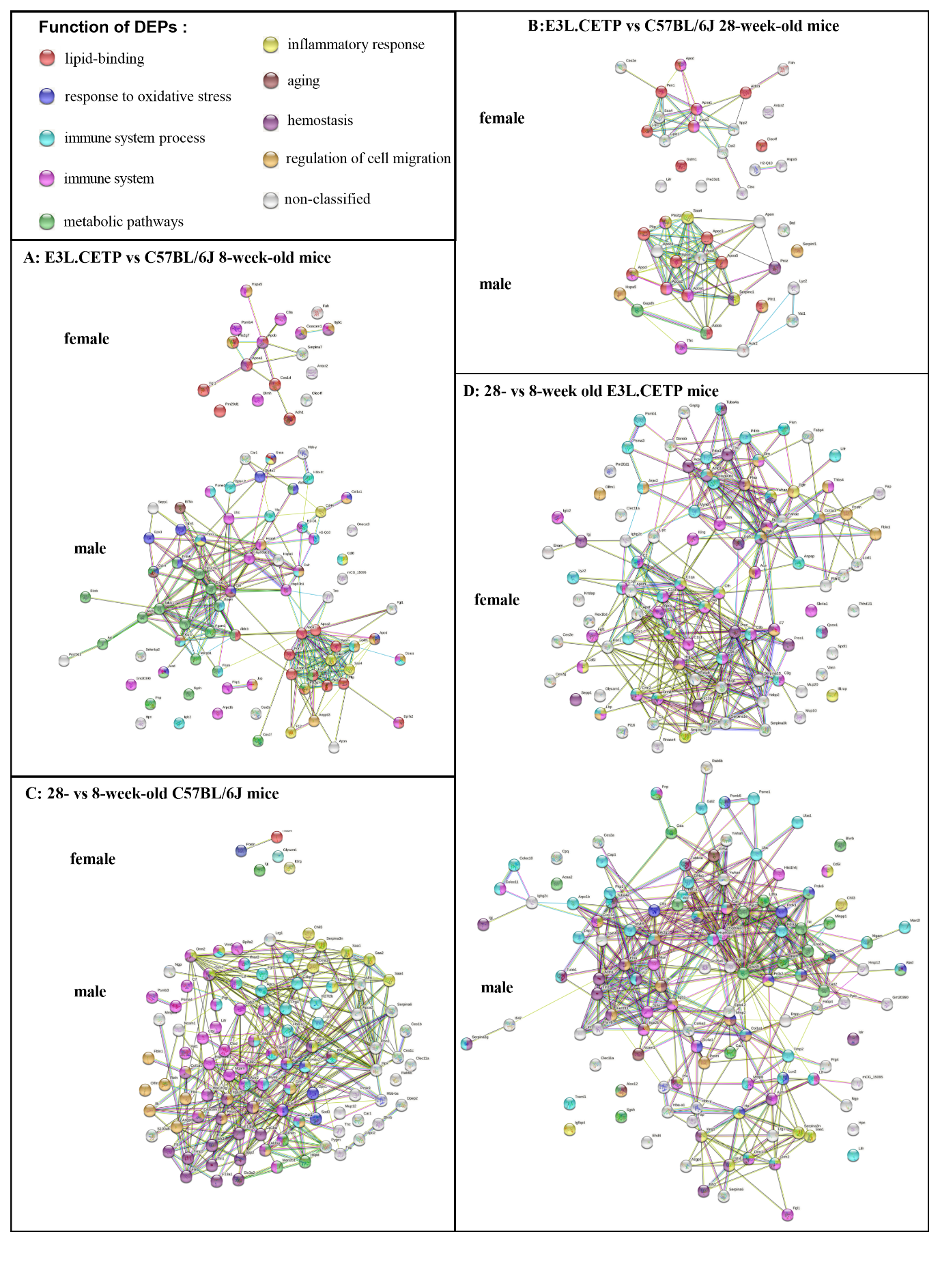


**Figure S12. Differential expressed proteins (DEPs) in plasma detected along ageing process in comparison to hyperlipidaemia-dependent DEPs in aortic proteome.** The most significant hyperlipidaemia-dependent proteins differentially expressed (DEPs; 2≤ratio≤0.5) in the male and female 8-week-old (**A**) and 28-week-old (**B**) E3L.CETP vs C57BL/6J mice as well as the most significant age-dependent DEPs in the C57BL/6J (**C**) and E3L.CETP (**D**) 8- vs 28-week-old mice. The DEPs were classified by function as lipid-binding (red colour), response to oxidative stress (dark blue colour), immune system (light blue and violet colour), metabolic pathways (green colour), inflammatory response (yellow colour), ageing (brown colour), hemostasis (dark violet colour), and regulation of cell migration (orange colour). The non-classified DEPs are marked as white. Created in STRING: functional protein association networks. The immunoglobulins are not presented in the graphics.


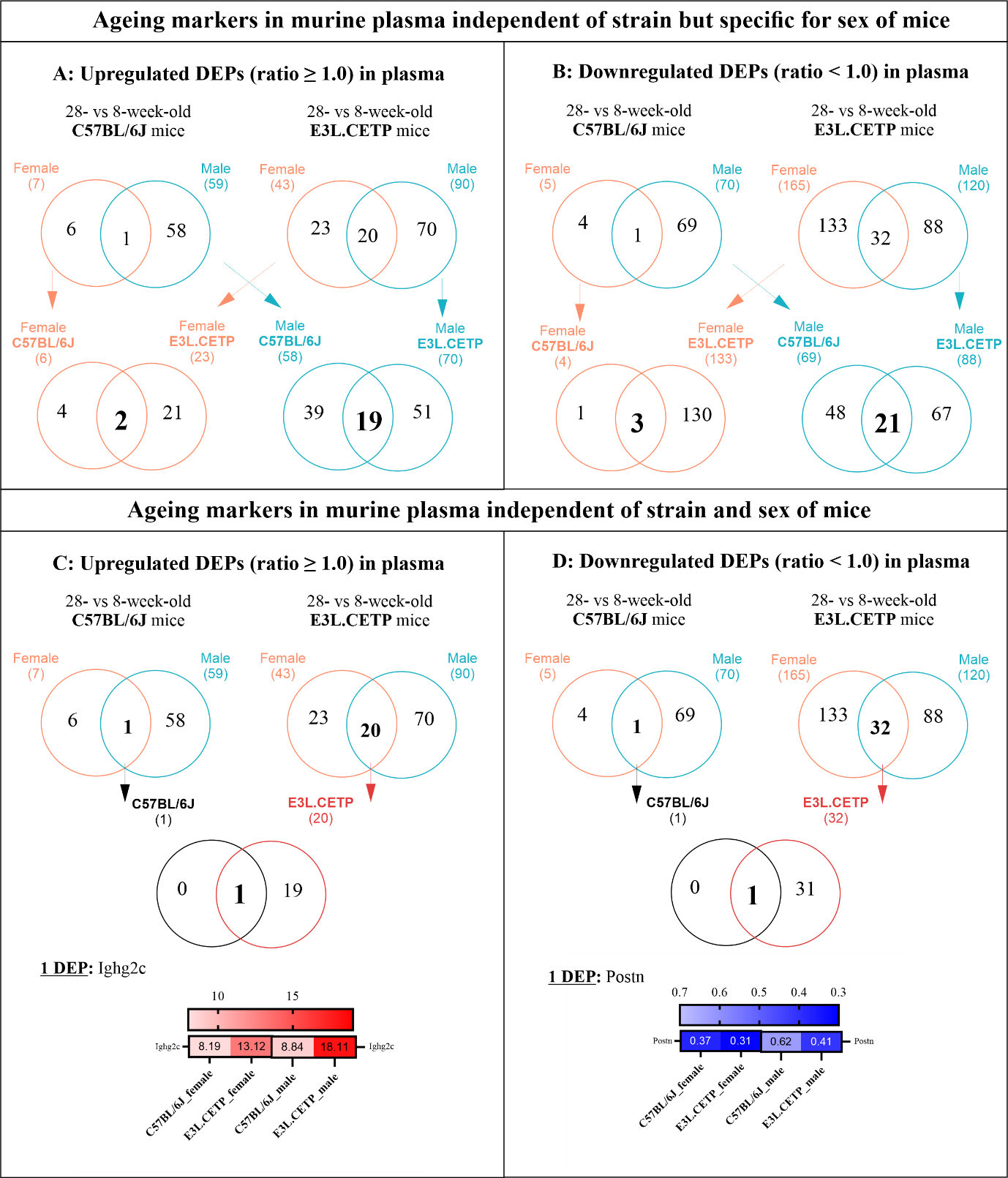


**Figure S13. Ageing markers (respective DEPs) in murine plasma independent of strain but specific for sex of mice as well as independent of strain and sex of mice.** Venn diagrams presenting unique and common DEPs (**A, C**: upregulated ≥ ratio 1.0 or **B, D**: downregulated < ratio 1.0) in plasma proteome between female and male mice in 28- vs 8-week-old E3L.CETP and C57BL/6J mice. From unique DEPs for female C57BL/6J and female E3L.CETP mice as well as from unique DEPs for male C57BL/6J and male E3L.CETP mice, common DEPs independent of strain were identified, specific for female and male, respectively (**A, B**). From common DEPs for female and male C57BL/6J mice as well as from common DEPs for female and male E3L.CETP mice, common DEPs independent of strain and sex of mice were identified (**C, D**).


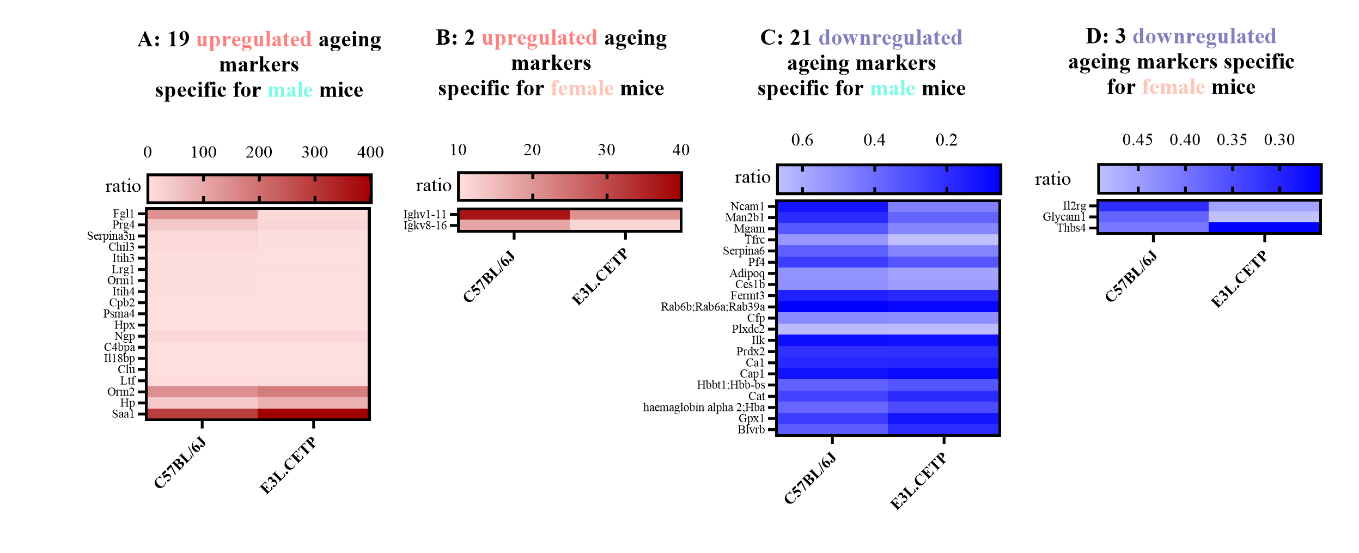


**Figure S14.** **Ageing markers (respective DEPs) in murine plasma independent of strain but specific for sex of mice.** Heatmaps of ratio of ageing (28-week-old vs 8-week-old) markers expression in murine aorta 19 specific for male (**A**) and 2 specific for female (**B**) upregulated (ratio ≥1.0) as well as 21 specific for male (**C**) and 3 specific for female (**D**) downregulated (ratio < 1.0) differentially expressed proteins (DEPs) in E3L.CETP and C57BL/6J mice. Heatmaps were created based on data presented in Supplementary Tables 16-19 (Table S16-S19).


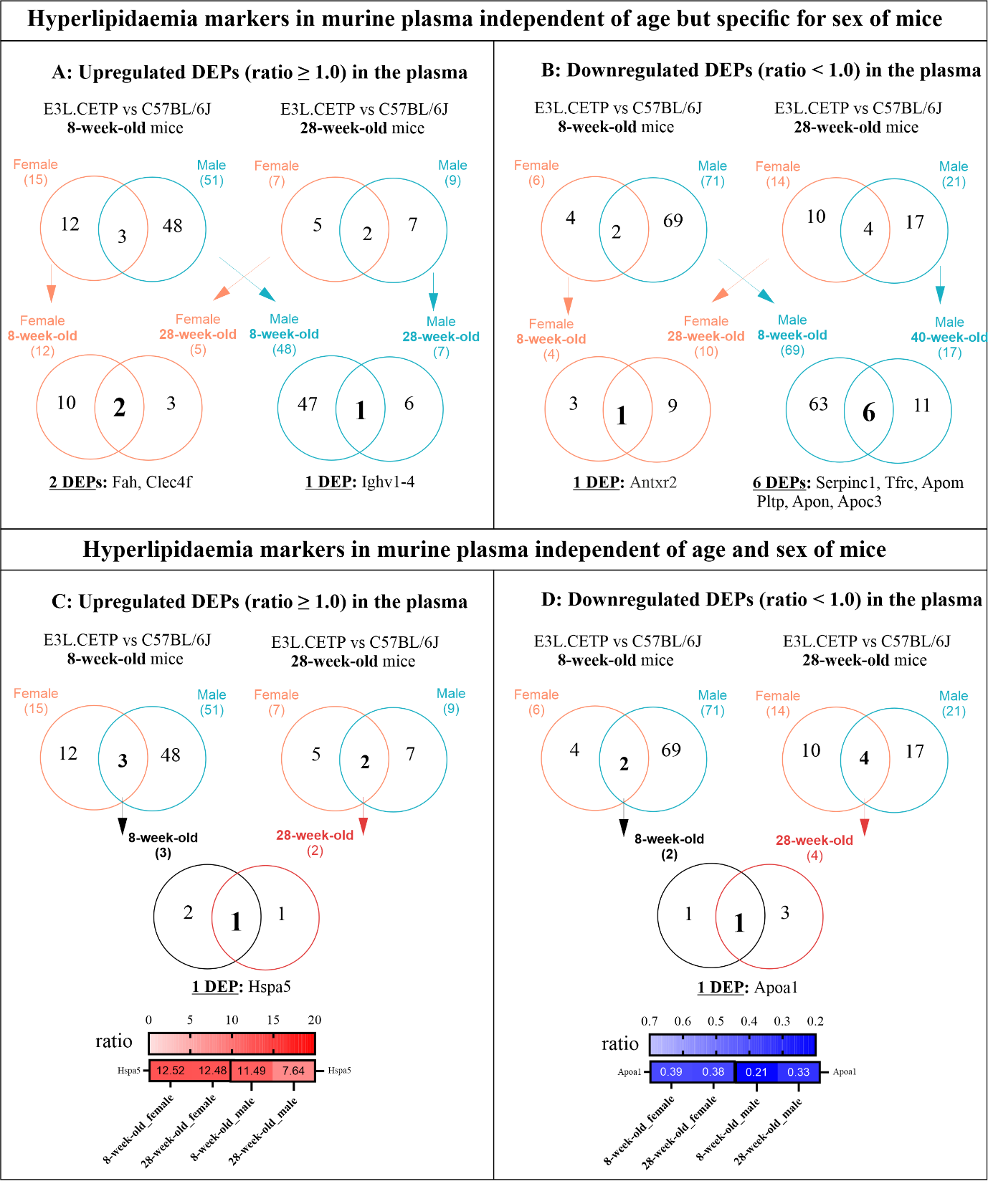


**Figure S15. Hyperlipidaemia markers (respective DEPs) in murine plasma independent of age but specific for sex of mice as well as independent of age and sex of mice.** Venn diagrams presenting unique and common DEPs (**A, C**: upregulated ≥ ratio 1.0 or **B, D**: downregulated < ratio 1.0) in plasma proteome between female and male mice in E3L.CETP vs C57BL/6J 8-week-old and 28-week-old mice. From unique DEPs for female 8-week-old and female 40-week-old mice as well as from unique DEPs for male 8-week-old and male 28-week-old mice, common DEPs independent of strain were identified, specific for female and male, respectively (**A, B**). From common DEPs for female and male 8-week-old mice as well as from common DEPs for female and male 28-week-old mice, common DEPs independent of age and sex of mice were identified (**C, D**).


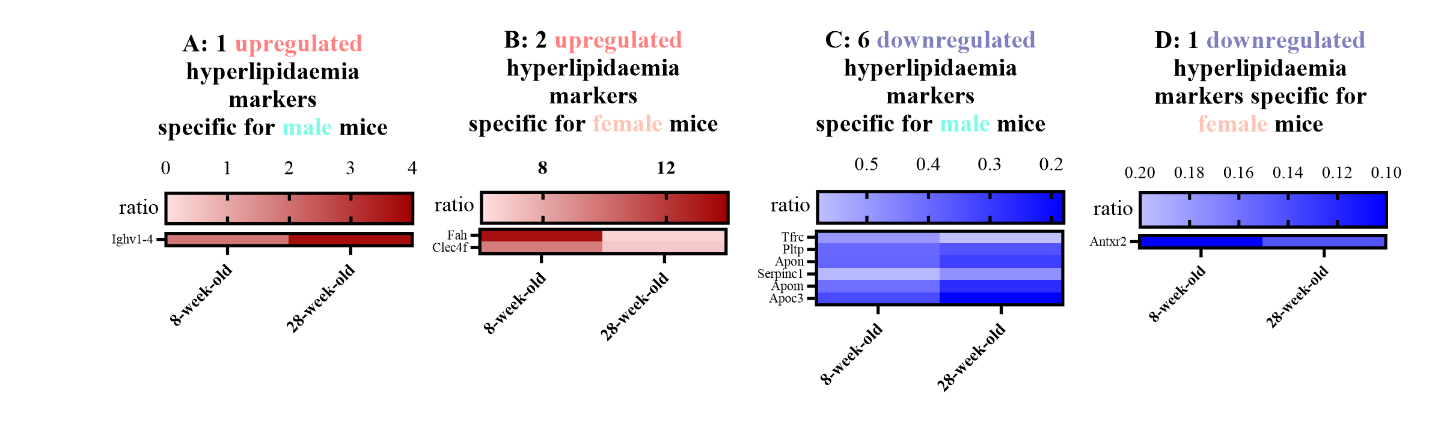


**Figure S16**. **Hyperlipidaemia markers (respective DEPs) in murine plasma independent of age but specific for sex of mice.**

Heatmaps of ratio of hyperlipidaemia (E3L.CETP vs C57BL/6J) markers expression in murine plasma 1 specific for male (**A**) and 2 specific for female (**B**) upregulated (ratio ≥1.0) as well as 6 specific for male (**C**) and 1 specific for female (**D**) downregulated (ratio < 1.0) differentially expressed proteins (DEPs) in 8-week-old and 28-week-old mice. Heatmaps were created based on data presented in Supplementary Tables 20-23 (Table S20-S23).

**
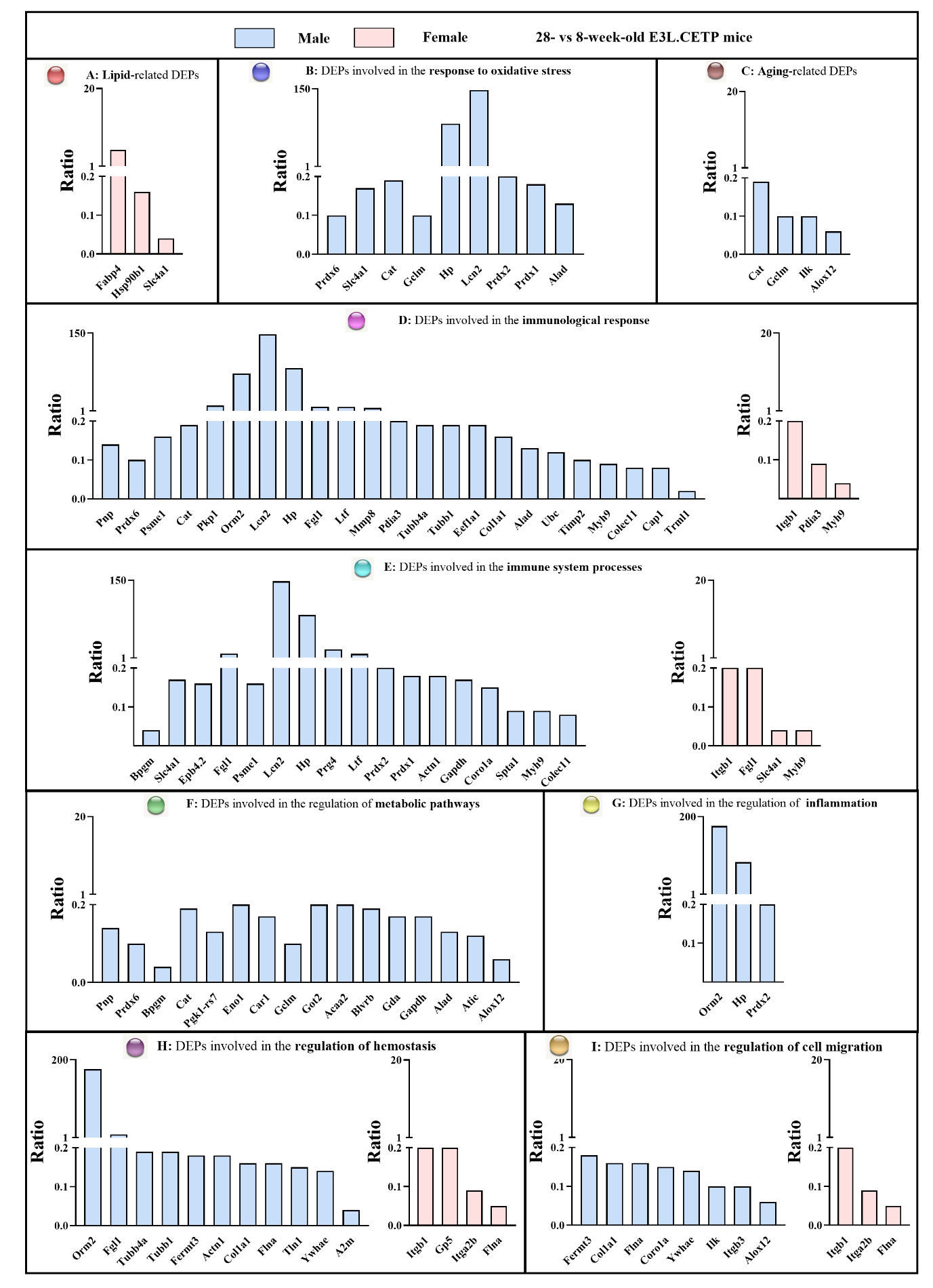
**

**Figure S17.** **The most significant ageing-dependent DEPs in plasma in E3L.CETP mice classified by processes.** The most significant age-dependent proteins differentially expressed (DEPs, 5≤ratio≤0.2) in E3L.CETP 8- vs 28-week-old male and female mice. DEPs were classified by function as lipid-binding proteins (**A**, red colour), response to oxidative stress (**B**, dark blue colour), ageing-related proteins (**C**, brown colour), immunological response (**D**, violet colour), regulation of immune system processes (**E,** light blue), regulation of metabolic pathways (**F**, green colour), inflammatory response (**G**, yellow colour), regulation of hemostasis (**H**, dark violet colour), and regulation of cell migration (**I**, orange colour). Created in STRING: functional protein association networks. The immunoglobulins are not presented in the graphics.


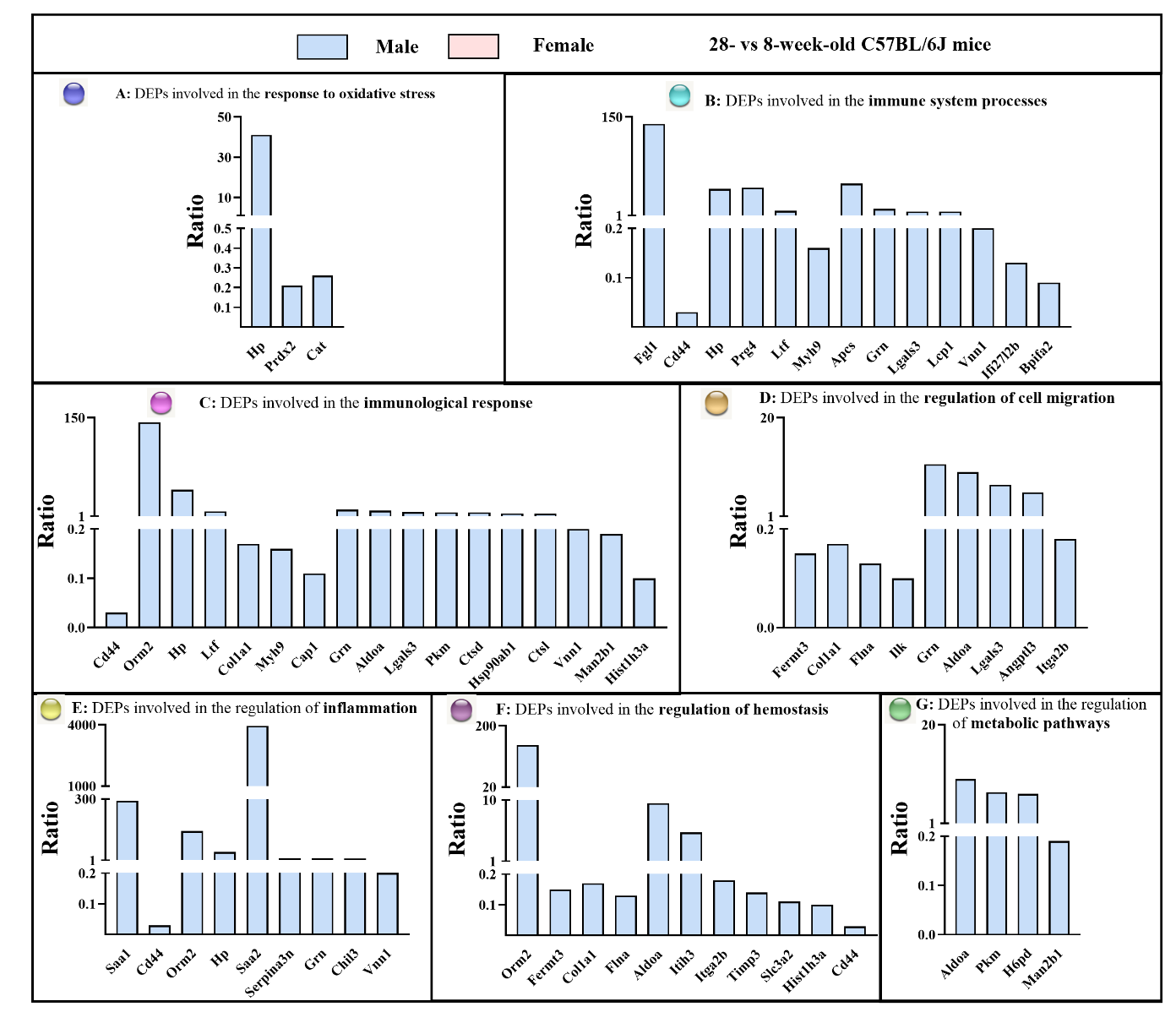


**Figure S18.** **The most significant ageing-dependent DEPs in plasma in C57BL/6J mice classified by processes.** The DEPs were classified by function as a response to oxidative stress (**A**, dark blue colour), regulation of immune system processes (**B**, light blue), immunological response (**C**, violet colour), regulation of cell migration (**D**, orange colour), inflammatory response (**E**, yellow colour), regulation of hemostasis (**F**, dark violet colour), and regulation of metabolic pathways (**G**, green colour). Created in STRING: functional protein association networks.


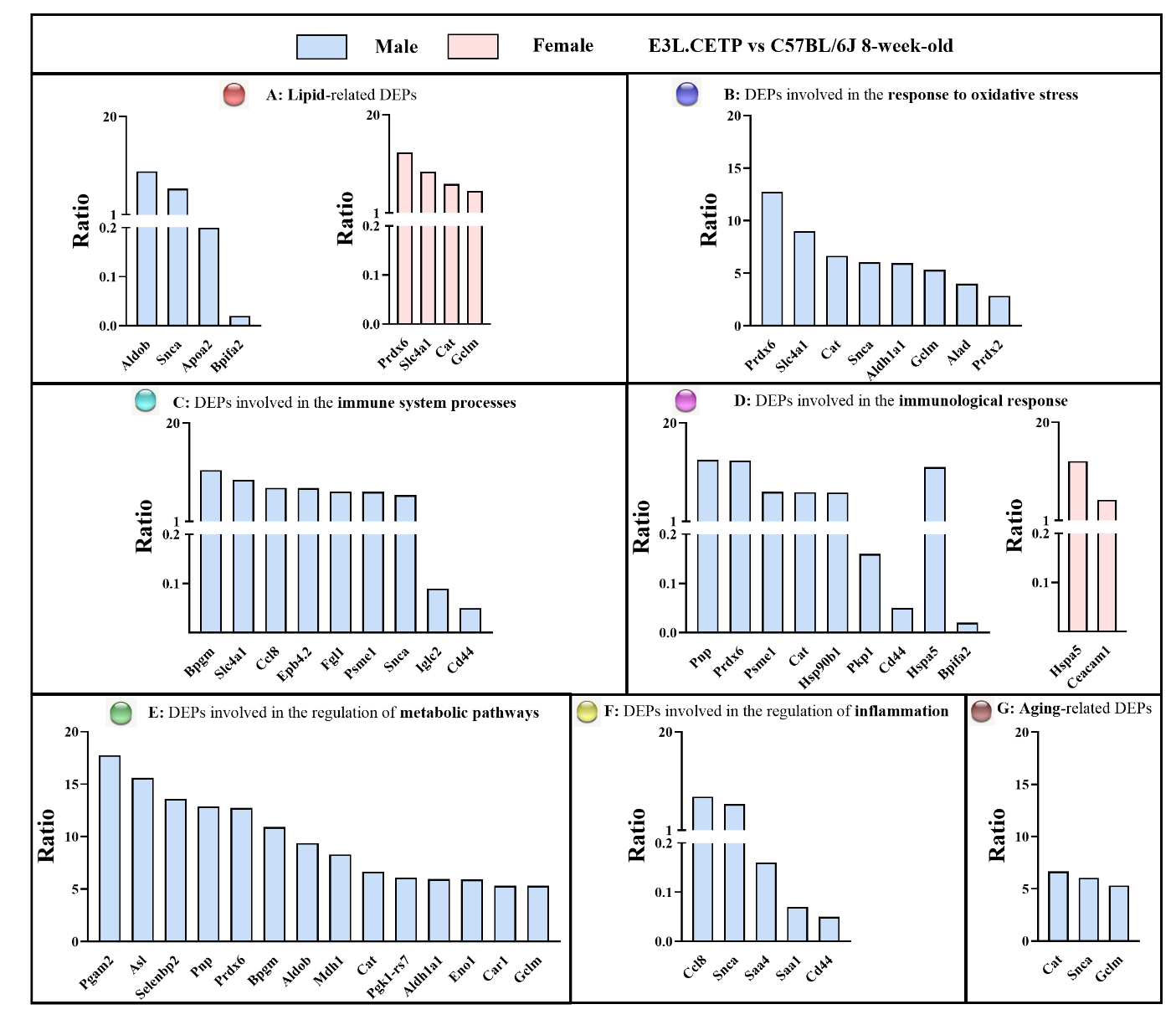


**Figure S19.** **The most significant** **hyperlipidaemia-dependent DEPs in plasma in 8-week-old mice classified by processes.** The most significant hyperlipidaemia-dependent proteins differentially expressed (DEPs, 5≤ratio≤0.2) in male and female 8-week-old E3L.CETP vs C57BL/6J mice. The DEPs were classified by function as lipid-binding proteins (**A**, red colour), response to oxidative stress (**B**, dark blue colour), regulation of immune system processes (**C**, light blue colour), immunological response (**D**, violet colour), regulation of metabolic pathways (**E**, green colour), inflammatory response (**F**, yellow colour), and ageing-related proteins (**G**, brown colour). Created in STRING: functional protein association networks. The immunoglobulins are not presented in the graphics.


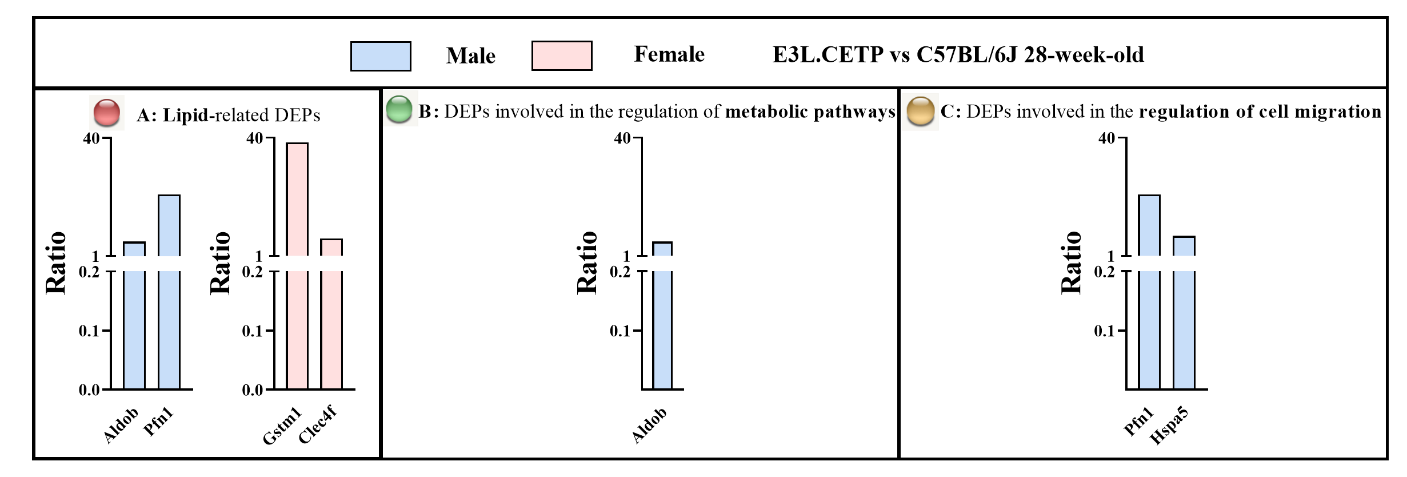


**Figure S20.** **The most significant hyperlipidaemia-dependent DEPs in plasma in 28-week-old mice classified by processes.** The most significant hyperlipidemia-dependent proteins differentially expressed (DEPs, 5≤ratio≤0.2) in male and female 28-week-old E3L.CETP vs C57BL/6J. DEPs were classified by function as lipid-binding proteins (**A**, red colour), regulation of metabolic pathways (**B**, green colour), and regulation of cell migration (**C**, orange colour). Created in STRING: functional protein association networks. The immunoglobulins are not presented in the graphics.


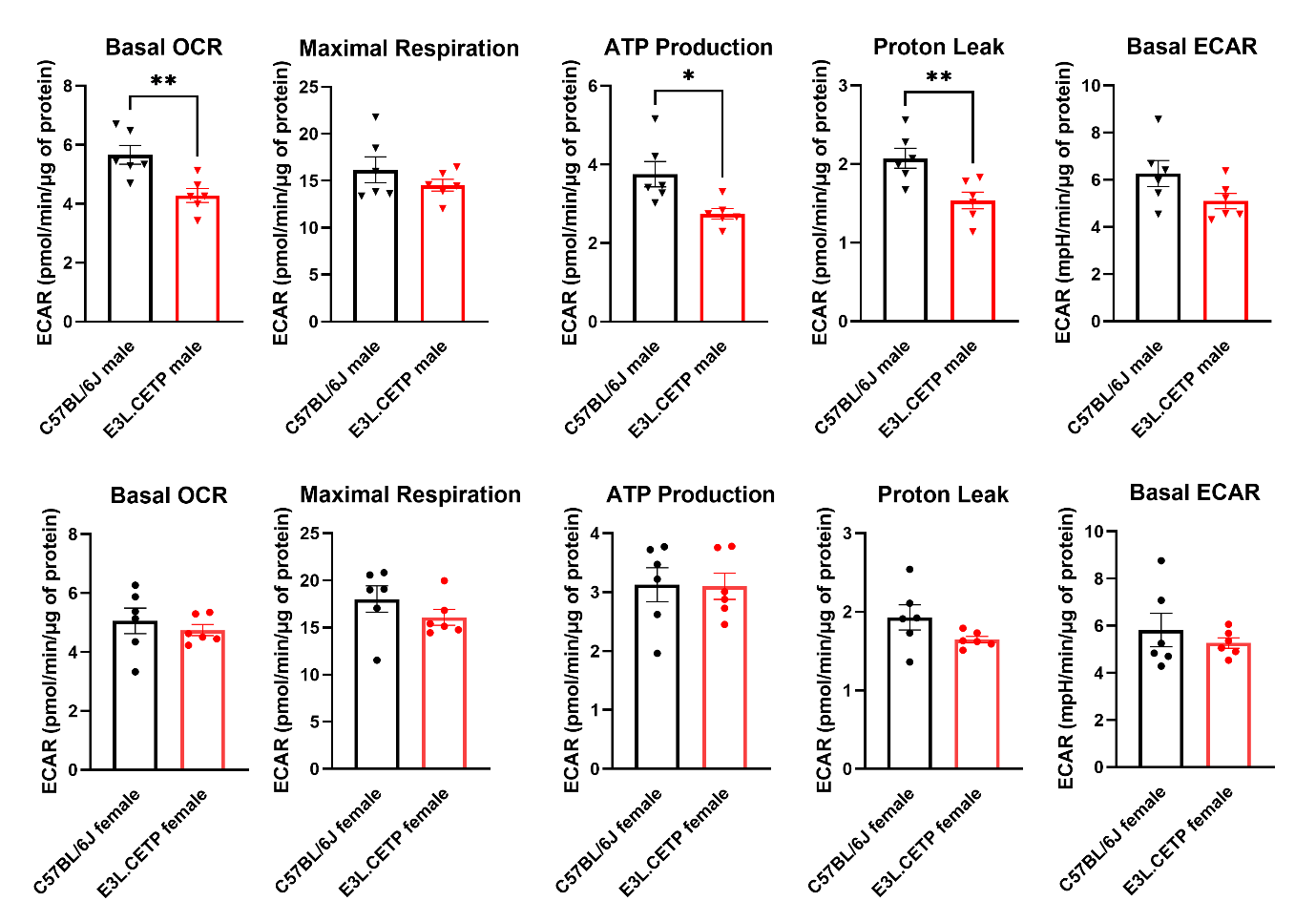


**Figure S21. Impaired vascular mitochondrial metabolism in old E3L.CETP male mice.** Energy metabolism profile was assessed in aortic rings isolated from old (48-week-old) E3L.CETP male and female mice as compared with old C57BL/6J male and female mice measured in single aortic rings, after incubation for 24h with interleukin-1β (IL-β, 1 ng/mL), using the Seahorse XFe96 Analyzer, with mitochondrial stress test (MST). OCR – oxygen consumption rate, ECAR - extracellular acidification rate. Data represent the means ± SEM (n = 5-6), analysed with t-test, *p ≤ 0.05, **p ≤ 0.01.

**SUPPLEMENTAL TABLES**

**Table S1.** Ageing markers (respective DEPs) in murine aorta independent of strain and sex of mice. Ratio of ageing (40-week old vs 8-week-old) markers expression in murine aorta of 159 upregulated (ratio ≥ 1.0) as well as 130 downregulated (ratio < 1.0) differentially expressed proteins (DEPs) in C57BL/6J female mice. Presented table was prepared based on proteins that were significantly different between compared groups. Statistics: Student’s t-test. Supplementary Table S1 refers to Figure 6 of the main manuscript.

| **No.** | **Protein ID** | **Protein annotation** | **Accession** | **Regulation** | **Ratio** |
| --- | --- | --- | --- | --- | --- |
| 1 | Ighg2a | Ig gamma-2A chain C region secreted form | P01865 | Up | 34.72 |
| 2 | Igh-3 | Ig gamma-2B chain C region | P01867 | Up | 22.03 |
| 3 | Ftl1 | Ferritin light chain 1 | P29391 | Up | 9.24 |
| 4 | Cma1 | Chymase | P21844 | Up | 6.07 |
| 5 | IGKC | Ig kappa chain C region | P01834 | Up | 5.97 |
| 6 | Vtn | Vitronectin | P29788 | Up | 5.00 |
| 7 | Mamdc2 | MAM domain-containing protein 2 | Q8CG85 | Up | 4.49 |
| 8 | Mgp | Matrix Gla protein | P19788 | Up | 4.28 |
| 9 | Mfge8 | Lactadherin | P21956 | Up | 4.07 |
| 10 | Rarres2 | Retinoic acid receptor responder protein 2 | Q9DD06 | Up | 3.96 |
| 11 | Ighm | Ig mu chain C region | P01872 | Up | 3.55 |
| 12 | Timp3 | Metalloproteinase inhibitor 3 | P39876 | Up | 3.45 |
| 13 | Htra1 | Serine protease HTRA1 | Q9R118 | Up | 3.37 |
| 14 | Sdcbp | Syntenin-1 | O08992 | Up | 3.29 |
| 15 | Trim47 | Tripartite motif-containing protein 47 | Q8C0E3 | Up | 3.16 |
| 16 | Vcan | Versican core protein | Q62059 | Up | 2.93 |
| 17 | Apoa4 | Apolipoprotein A-IV | P06728 | Up | 2.81 |
| 18 | Nebl | LIM zinc-binding domain-containing Nebulette | Q9DC07 | Up | 2.76 |
| 19 | Hapln1 | Hyaluronan and proteoglycan link protein 1 | Q9QUP5 | Up | 2.69 |
| 20 | Slc29a1 | Equilibrative nucleoside transporter 1 | Q9JIM1 | Up | 2.60 |
| 21 | Axl | Tyrosine-protein kinase receptor UFO | Q00993 | Up | 2.38 |
| 22 | Ntn1 | Netrin-1 | O09118 | Up | 2.27 |
| 23 | Fth1 | Ferritin heavy chain | P09528 | Up | 2.17 |
| 24 | Sbspon | Somatomedin-B and thrombospondin type-1 domain-containing protein | Q3UPR9 | Up | 2.13 |
| 25 | Loxl3 | Lysyl oxidase homolog 3 | Q9Z175 | Up | 2.06 |
| 26 | Aspn | Asporin | Q99MQ4 | Up | 2.05 |
| 27 | Plg | Plasminogen | P20918 | Up | 2.04 |
| 28 | Atp2a3 | Sarcoplasmic/endoplasmic reticulum calcium ATPase 3 | Q64518 | Up | 2.02 |
| 29 | Clu | Clusterin | Q06890 | Up | 1.99 |
| 30 | Ston1 | Stonin-1 | Q8CDJ8 | Up | 1.88 |
| 31 | Itgb5 | Integrin beta-5 | O70309 | Up | 1.84 |
| 32 | Serpinc1 | Antithrombin-III | P32261 | Up | 1.83 |
| 33 | Susd2 | Sushi domain-containing protein 2 | Q9DBX3 | Up | 1.80 |
| 34 | Tinagl1 | Tubulointerstitial nephritis antigen-like | Q99JR5 | Up | 1.79 |
| 35 | App | Amyloid beta A4 protein | P12023 | Up | 1.79 |
| 36 | Serpina1b | Alpha-1-antitrypsin 1-2 | P22599 | Up | 1.77 |
| 37 | Bcam | Basal cell adhesion molecule | Q9R069 | Up | 1.76 |
| 38 | Maob | Amine oxidase [flavin-containing] B | Q8BW75 | Up | 1.70 |
| 39 | Cfd | Complement factor D | P03953 | Up | 1.67 |
| 40 | Col6a2 | Collagen alpha-2(VI) chain | Q02788 | Up | 1.65 |
| 41 | Col6a1 | Collagen alpha-1(VI) chain | Q04857 | Up | 1.65 |
| 42 | Agrn | Agrin | A2ASQ1 | Up | 1.63 |
| 43 | Fhl3 | Four and a half LIM domains protein 3 | Q9R059 | Up | 1.62 |
| 44 | Cr1l | Complement component receptor 1-like protein | Q64735 | Up | 1.57 |
| 45 | Aldh9a1 | 4-trimethylaminobutyraldehyde dehydrogenase | Q9JLJ2 | Up | 1.55 |
| 46 | Fbln2 | Fibulin-2 | P37889 | Up | 1.55 |
| 47 | Inmt | Indolethylamine N-methyltransferase | P40936 | Up | 1.53 |
| 48 | Lama5 | Laminin subunit alpha-5 | Q61001 | Up | 1.53 |
| 49 | Rnase4 | Ribonuclease 4 | Q9JJH1 | Up | 1.50 |
| 50 | Lamb2 | Laminin subunit beta-2 | Q61292 | Up | 1.48 |
| 51 | Prelp | Prolargin | Q9JK53 | Up | 1.48 |
| 52 | Akr1b8 | Aldose reductase-related protein 2 | P45377 | Up | 1.47 |
| 53 | Itga3 | Integrin alpha-3 | Q62470 | Up | 1.45 |
| 54 | Lamc1 | Laminin subunit gamma-1 | P02468 | Up | 1.45 |
| 55 | Fn1 | Fibronectin;Anastellin | P11276 | Up | 1.45 |
| 56 | Cd109 | CD109 antigen | Q8R422 | Up | 1.45 |
| 57 | Serpina1c;Serpina1a | Alpha-1-antitrypsin 1-3;Alpha-1-antitrypsin 1-1 | Q00896 | Up | 1.44 |
| 58 | Mfap4 | Microfibril-associated glycoprotein 4 | Q9D1H9 | Up | 1.44 |
| 59 | Cbr1 | Carbonyl reductase [NADPH] 1 | P48758 | Up | 1.43 |
| 60 | Tagln2 | Transgelin-2 | Q9WVA4 | Up | 1.40 |
| 61 | Gstm1 | Glutathione S-transferase Mu 1 | P10649 | Up | 1.40 |
| 62 | Efemp1 | EGF-containing fibulin-like extracellular matrix protein 1 | Q8BPB5 | Up | 1.39 |
| 63 | Itgav | Integrin alpha-V | P43406 | Up | 1.39 |
| 64 | Fhl1 | Four and a half LIM domains protein 1 | P97447 | Up | 1.38 |
| 65 | Tes | Testin | P47226 | Up | 1.37 |
| 66 | Mcam | Cell surface glycoprotein MUC18 | Q8R2Y2 | Up | 1.35 |
| 67 | Efemp2 | EGF-containing fibulin-like extracellular matrix protein 2 | Q9WVJ9 | Up | 1.35 |
| 68 | Selenbp1 | Selenium-binding protein 1;Selenium-binding protein 2 | P17563 | Up | 1.35 |
| 69 | Nptn | Neuroplastin | P97300 | Up | 1.35 |
| 70 | Abhd14b | Alpha/beta hydrolase domain-containing protein 14B | Q8VCR7 | Up | 1.35 |
| 71 | Sfxn3 | Sideroflexin-3 | Q91V61 | Up | 1.32 |
| 72 | Maoa | Amine oxidase [flavin-containing] A | Q64133 | Up | 1.32 |
| 73 | Col18a1 | Collagen alpha-1(XVIII) chain;Endostatin | P39061 | Up | 1.32 |
| 74 | Gstp1 | Glutathione S-transferase P 1 | P19157 | Up | 1.32 |
| 75 | Bgn | Biglycan | P28653 | Up | 1.31 |
| 76 | Acan | Aggrecan core protein | Q61282 | Up | 1.31 |
| 77 | G6pdx | Glucose-6-phosphate 1-dehydrogenase X | Q00612 | Up | 1.31 |
| 78 | Fblim1 | Filamin-binding LIM protein 1 | Q71FD7 | Up | 1.31 |
| 79 | Slc44a2 | Choline transporter-like protein 2 | Q8BY89 | Up | 1.30 |
| 80 | Cnn2 | Calponin-2 | Q08093 | Up | 1.30 |
| 81 | Ola1 | Obg-like ATPase 1 | Q9CZ30 | Up | 1.29 |
| 82 | Cyb5r3 | NADH-cytochrome b5 reductase 3 | Q9DCN2 | Up | 1.29 |
| 83 | Itgb1 | Integrin beta-1 | P09055 | Up | 1.29 |
| 84 | Lum | Lumican | P51885 | Up | 1.29 |
| 85 | Lama4 | Laminin subunit alpha-4 | P97927 | Up | 1.28 |
| 86 | Entpd1 | Ectonucleoside triphosphate diphosphohydrolase 1 | P55772 | Up | 1.28 |
| 87 | Cd9 | CD9 antigen | P40240 | Up | 1.27 |
| 88 | Atp1b3 | Sodium/potassium-transporting ATPase subunit beta-3 | P97370 | Up | 1.27 |
| 89 | Col4a2 | Collagen alpha-2(IV) chain;Canstatin | P08122 | Up | 1.26 |
| 90 | Pdcd6 | Programmed cell death protein 6 | P12815 | Up | 1.26 |
| 91 | Flot1 | Flotillin-1 | O08917 | Up | 1.26 |
| 92 | Rras | Ras-related protein R-Ras | P10833 | Up | 1.26 |
| 93 | Dcn | Decorin | P28654 | Up | 1.26 |
| 94 | Anxa4 | Annexin A4 | P97429 | Up | 1.25 |
| 95 | Adipoq | Adiponectin | Q60994 | Up | 1.25 |
| 96 | Tns2 | Tensin-2 | Q8CGB6 | Up | 1.25 |
| 97 | Vwf | von Willebrand factor;von Willebrand antigen 2 | Q8CIZ8 | Up | 1.25 |
| 98 | Pdxk | Pyridoxal kinase | Q8K183 | Up | 1.24 |
| 99 | Eml2 | Echinoderm microtubule-associated protein-like 2 | Q7TNG5 | Up | 1.24 |
| 100 | Atic | Bifunctional purine biosynthesis protein PURH | Q9CWJ9 | Up | 1.23 |
| 101 | Ndrg1 | Protein NDRG1 | Q62433 | Up | 1.23 |
| 102 | Gpi | Glucose-6-phosphate isomerase | P06745 | Up | 1.23 |
| 103 | Park7 | Protein deglycase DJ-1 | Q99LX0 | Up | 1.23 |
| 104 | Pgm5 | Phosphoglucomutase-like protein 5 | Q8BZF8 | Up | 1.23 |
| 105 | Cd200 | OX-2 membrane glycoprotein | O54901 | Up | 1.23 |
| 106 | Entpd2 | Ectonucleoside triphosphate diphosphohydrolase 2 | O55026 | Up | 1.22 |
| 107 | Gstt3 | Glutathione S-transferase theta-3 | Q99L20 | Up | 1.22 |
| 108 | Tpm3 | Tropomyosin alpha-3 chain | P21107 | Up | 1.22 |
| 109 | Col4a1 | Collagen alpha-1(IV) chain;Arresten | P02463 | Up | 1.22 |
| 110 | Sntb2 | Beta-2-syntrophin | Q61235 | Up | 1.21 |
| 111 | Rhoa | Transforming protein RhoA | Q9QUI0 | Up | 1.21 |
| 112 | Spr | Sepiapterin reductase | Q64105 | Up | 1.20 |
| 113 | Gapdh | Glyceraldehyde-3-phosphate dehydrogenase | P16858 | Up | 1.20 |
| 114 | Blvra | Biliverdin reductase A | Q9CY64 | Up | 1.19 |
| 115 | Tgfbi | Transforming growth factor-beta-induced protein ig-h3 | P82198 | Up | 1.19 |
| 116 | Tgm2 | Protein-glutamine gamma-glutamyltransferase 2 | P21981 | Up | 1.19 |
| 117 | Dag1 | Dystroglycan | Q62165 | Up | 1.19 |
| 118 | Itga8 | Integrin alpha-8 | A2ARA8 | Up | 1.18 |
| 119 | Dtna | Dystrobrevin alpha | Q9D2N4 | Up | 1.18 |
| 120 | Tpm1 | Tropomyosin alpha-1 chain | P58771 | Up | 1.18 |
| 121 | Gyg1 | Glycogenin-1 | Q9R062 | Up | 1.18 |
| 122 | Hspg2 | Basement membrane-specific heparan sulfate proteoglycan core protein | Q05793 | Up | 1.17 |
| 123 | Gstm2 | Glutathione S-transferase Mu 2 | P15626 | Up | 1.17 |
| 124 | Ephx1 | Epoxide hydrolase 1 | Q9D379 | Up | 1.17 |
| 125 | Aldh2 | Aldehyde dehydrogenase. mitochondrial | P47738 | Up | 1.17 |
| 126 | Sorbs2 | Sorbin and SH3 domain-containing protein 2 | Q3UTJ2 | Up | 1.17 |
| 127 | Cacna2d1 | Voltage-dependent calcium channel subunit alpha-2/delta-1 | O08532 | Up | 1.16 |
| 128 | Smtn | Smoothelin | Q921U8 | Up | 1.15 |
| 129 | Anxa11 | Annexin A11 | P97384 | Up | 1.15 |
| 130 | Nqo1 | NAD(P)H dehydrogenase [quinone] 1 | Q64669 | Up | 1.15 |
| 131 | Pkm | Pyruvate kinase PKM | P52480 | Up | 1.15 |
| 132 | Asah1 | Acid ceramidase | Q9WV54 | Up | 1.15 |
| 133 | Cdh13 | Cadherin-13 | Q9WTR5 | Up | 1.15 |
| 134 | Adh5 | Alcohol dehydrogenase class-3 | P28474 | Up | 1.14 |
| 135 | Cbr3 | Carbonyl reductase [NADPH] 3 | Q8K354 | Up | 1.14 |
| 136 | Msn | Moesin | P26041 | Up | 1.14 |
| 137 | Cp | Ceruloplasmin | Q61147 | Up | 1.14 |
| 138 | Rab18 | Ras-related protein Rab-18 | P35293 | Up | 1.14 |
| 139 | Gnai2 | Guanine nucleotide-binding protein G(i) subunit alpha-2 | P08752 | Up | 1.13 |
| 140 | Ehd4 | EH domain-containing protein 4 | Q9EQP2 | Up | 1.13 |
| 141 | Adh1 | Alcohol dehydrogenase 1 | P00329 | Up | 1.13 |
| 142 | Dmd | Dystrophin | P11531 | Up | 1.13 |
| 143 | Ptgis | Prostacyclin synthase | O35074 | Up | 1.13 |
| 144 | Acta2 | Actin. aortic smooth muscle | P62737 | Up | 1.12 |
| 145 | Glod4 | Glyoxalase domain-containing protein 4 | Q9CPV4 | Up | 1.12 |
| 146 | Gstt1 | Glutathione S-transferase theta-1 | Q64471 | Up | 1.11 |
| 147 | Ppia | Peptidyl-prolyl cis-trans isomerase A | P17742 | Up | 1.11 |
| 148 | Flna | Filamin-A | Q8BTM8 | Up | 1.11 |
| 149 | Ilk | Integrin-linked protein kinase | O55222 | Up | 1.08 |
| 150 | Csrp1 | Cysteine and glycine-rich protein 1 | P97315 | Up | 1.08 |
| 151 | Ehd1 | EH domain-containing protein 1 | Q9WVK4 | Up | 1.08 |
| 152 | Got2 | Aspartate aminotransferase. mitochondrial | P05202 | Up | 1.07 |
| 153 | Atp1a1 | Sodium/potassium-transporting ATPase subunit alpha-1 | Q8VDN2 | Up | 1.07 |
| 154 | Aldoa | Fructose-bisphosphate aldolase A | P05064 | Up | 1.06 |
| 155 | Camk2g | Calcium/calmodulin-dependent protein kinase type II subunit gamma | Q923T9 | Up | 1.06 |
| 156 | Anxa1 | Annexin A1 | P10107 | Up | 1.06 |
| 157 | Pfkp | ATP-dependent 6-phosphofructokinase. platelet type | Q9WUA3 | Up | 1.06 |
| 158 | Tln1 | Talin-1 | P26039 | Up | 1.05 |
| 159 | Fermt2 | Fermitin family homolog 2 | Q8CIB5 | Up | 1.04 |
| 160 | Idh1 | Isocitrate dehydrogenase [NADP] cytoplasmic | O88844 | Down | 0.98 |
| 161 | Cdc42bpb | Serine/threonine-protein kinase MRCK beta | Q7TT50 | Down | 0.96 |
| 162 | Tkt | Transketolase | P40142 | Down | 0.95 |
| 163 | Calu | Calumenin | O35887 | Down | 0.94 |
| 164 | Rpl11 | 60S ribosomal protein L11 | Q9CXW4 | Down | 0.91 |
| 165 | Aco1 | Cytoplasmic aconitate hydratase | P28271 | Down | 0.91 |
| 166 | Sept9 | Septin-9 | Q80UG5 | Down | 0.91 |
| 167 | Sec13 | Protein SEC13 homolog | Q9D1M0 | Down | 0.90 |
| 168 | Hnrnpa3 | Heterogeneous nuclear ribonucleoprotein A3 | Q8BG05 | Down | 0.90 |
| 169 | Eprs | Bifunctional glutamate/proline--tRNA ligase | Q8CGC7 | Down | 0.89 |
| 170 | Rpl4 | 60S ribosomal protein L4 | Q9D8E6 | Down | 0.89 |
| 171 | Uso1 | General vesicular transport factor p115 | Q9Z1Z0 | Down | 0.89 |
| 172 | Hnrnpf | Heterogeneous nuclear ribonucleoprotein F | Q9Z2X1 | Down | 0.89 |
| 173 | Pabpc1 | Polyadenylate-binding protein 1 | P29341 | Down | 0.88 |
| 174 | Pdia4 | Protein disulfide-isomerase A4 | P08003 | Down | 0.87 |
| 175 | Rps17 | 40S ribosomal protein S17 | P63276 | Down | 0.87 |
| 176 | Rpl8 | 60S ribosomal protein L8 | P62918 | Down | 0.87 |
| 177 | Rpl9 | 60S ribosomal protein L9 | P51410 | Down | 0.86 |
| 178 | Dhx9 | ATP-dependent RNA helicase A | O70133 | Down | 0.86 |
| 179 | Syncrip | Heterogeneous nuclear ribonucleoprotein Q | Q7TMK9 | Down | 0.86 |
| 180 | Tppp3 | Tubulin polymerization-promoting protein family member 3 | Q9CRB6 | Down | 0.86 |
| 181 | Rpl12 | 60S ribosomal protein L12 | P35979 | Down | 0.86 |
| 182 | Erp44 | Endoplasmic reticulum resident protein 44 | Q9D1Q6 | Down | 0.86 |
| 183 | Pdia3 | Protein disulfide-isomerase A3 | P27773 | Down | 0.86 |
| 184 | Khsrp | Far upstream element-binding protein 2 | Q3U0V1 | Down | 0.85 |
| 185 | FAM120A | Constitutive coactivator of PPAR-gamma-like protein 1 | Q6A0A9 | Down | 0.85 |
| 186 | Fstl1 | Follistatin-related protein 1 | Q62356 | Down | 0.85 |
| 187 | Ssb | Lupus La protein homolog | P32067 | Down | 0.85 |
| 188 | Calr | Calreticulin | P14211 | Down | 0.85 |
| 189 | Copa | Coatomer subunit alpha;Xenin;Proxenin | Q8CIE6 | Down | 0.85 |
| 190 | Rps2 | 40S ribosomal protein S2 | P25444 | Down | 0.84 |
| 191 | Ncl | Nucleolin | P09405 | Down | 0.84 |
| 192 | Copg1 | Coatomer subunit gamma-1 | Q9QZE5 | Down | 0.84 |
| 193 | Hnrnpd | Heterogeneous nuclear ribonucleoprotein D0 | Q60668 | Down | 0.84 |
| 194 | Sec22b | Vesicle-trafficking protein SEC22b | O08547 | Down | 0.84 |
| 195 | Rpl6 | 60S ribosomal protein L6 | P47911 | Down | 0.84 |
| 196 | Rpn2 | Dolichyl-diphosphooligosaccharide--protein glycosyltransferase subunit 2 | Q9DBG6 | Down | 0.84 |
| 197 | Rpl13 | 60S ribosomal protein L13 | P47963 | Down | 0.84 |
| 198 | Cope | Coatomer subunit epsilon | O89079 | Down | 0.83 |
| 199 | Rpl3 | 60S ribosomal protein L3 | P27659 | Down | 0.83 |
| 200 | Ybx1 | Nuclease-sensitive element-binding protein 1 | P62960 | Down | 0.83 |
| 201 | Copb2 | Coatomer subunit beta | O55029 | Down | 0.83 |
| 202 | Cav2 | Caveolin-2 | Q9WVC3 | Down | 0.83 |
| 203 | Asph | Aspartyl/asparaginyl beta-hydroxylase | Q8BSY0 | Down | 0.83 |
| 204 | Hsp90b1 | Endoplasmin | P08113 | Down | 0.83 |
| 205 | Eef1b | Elongation factor 1-beta | O70251 | Down | 0.83 |
| 206 | Gnb2l1 | Guanine nucleotide-binding protein subunit beta-2-like 1 | P68040 | Down | 0.83 |
| 207 | Hnrnpl | Heterogeneous nuclear ribonucleoprotein L | Q8R081 | Down | 0.83 |
| 208 | Rpn1 | Dolichyl-diphosphooligosaccharide--protein glycosyltransferase subunit 1 | Q91YQ5 | Down | 0.82 |
| 209 | Eif4g1 | Eukaryotic translation initiation factor 4 gamma 1 | Q6NZJ6 | Down | 0.82 |
| 210 | Fmod | Fibromodulin | P50608 | Down | 0.82 |
| 211 | Txndc5 | Thioredoxin domain-containing protein 5 | Q91W90 | Down | 0.82 |
| 212 | Hspa5 | 78 kDa glucose-regulated protein | P20029 | Down | 0.82 |
| 213 | Blmh | Bleomycin hydrolase | Q8R016 | Down | 0.82 |
| 214 | Hnrnph1 | Heterogeneous nuclear ribonucleoprotein H | O35737 | Down | 0.82 |
| 215 | Trim28 | Transcription intermediary factor 1-beta | Q62318 | Down | 0.82 |
| 216 | Hnrnpul2 | Heterogeneous nuclear ribonucleoprotein U-like protein 2 | Q00PI9 | Down | 0.82 |
| 217 | Rpl17 | 60S ribosomal protein L17 | Q9CPR4 | Down | 0.81 |
| 218 | P4hb | Protein disulfide-isomerase | P09103 | Down | 0.81 |
| 219 | Sf1 | Splicing factor 1 | Q64213 | Down | 0.81 |
| 220 | Rps14 | 40S ribosomal protein S14 | P62264 | Down | 0.81 |
| 221 | Ddx39b | Spliceosome RNA helicase Ddx39b | Q9Z1N5 | Down | 0.80 |
| 222 | Rpl5 | 60S ribosomal protein L5 | P47962 | Down | 0.80 |
| 223 | Rps3a | 40S ribosomal protein S3a | P97351 | Down | 0.80 |
| 224 | Tmed10 | Transmembrane emp24 domain-containing protein 10 | Q9D1D4 | Down | 0.80 |
| 225 | Hnrnpa2b1 | Heterogeneous nuclear ribonucleoproteins A2/B1 | O88569 | Down | 0.80 |
| 226 | Ppib | Peptidyl-prolyl cis-trans isomerase B | P24369 | Down | 0.79 |
| 227 | Nid2 | Nidogen-2 | O88322 | Down | 0.79 |
| 228 | Fkbp4 | Peptidyl-prolyl cis-trans isomerase FKBP4 | P30416 | Down | 0.79 |
| 229 | Rplp0 | 60S acidic ribosomal protein P0 | P14869 | Down | 0.79 |
| 230 | Rps15 | 40S ribosomal protein S15 | P62843 | Down | 0.79 |
| 231 | Rps12 | 40S ribosomal protein S12 | P63323 | Down | 0.79 |
| 232 | Rpl31 | 60S ribosomal protein L31 | P62900 | Down | 0.79 |
| 233 | Rps7 | 40S ribosomal protein S7 | P62082 | Down | 0.78 |
| 234 | Copb1 | Coatomer subunit beta | Q9JIF7 | Down | 0.78 |
| 235 | Hnrnpm | Heterogeneous nuclear ribonucleoprotein M | Q9D0E1 | Down | 0.78 |
| 236 | Rps8 | 40S ribosomal protein S8 | P62242 | Down | 0.78 |
| 237 | Cbr2 | Carbonyl reductase [NADPH] 2 | P08074 | Down | 0.77 |
| 238 | Serbp1 | Plasminogen activator inhibitor 1 RNA-binding protein | Q9CY58 | Down | 0.77 |
| 239 | Hnrnpa1 | Heterogeneous nuclear ribonucleoprotein A1 | P49312 | Down | 0.77 |
| 240 | Btf3 | Transcription factor BTF3 | Q64152 | Down | 0.77 |
| 241 | Lasp1 | LIM and SH3 domain protein 1 | Q61792 | Down | 0.76 |
| 242 | Rps20 | 40S ribosomal protein S20 | P60867 | Down | 0.76 |
| 243 | Rpl23 | 60S ribosomal protein L23 | P62830 | Down | 0.76 |
| 244 | Eef1d | Elongation factor 1-delta | P57776 | Down | 0.75 |
| 245 | Arcn1 | Coatomer subunit delta | Q5XJY5 | Down | 0.75 |
| 246 | Rbbp7 | Histone-binding protein RBBP7 | Q60973 | Down | 0.75 |
| 247 | Hnrnpk | Heterogeneous nuclear ribonucleoprotein K | P61979 | Down | 0.75 |
| 248 | H2afv;H2afz | Histone H2A.V | Q3THW5 | Down | 0.74 |
| 249 | Hdlbp | Vigilin | Q8VDJ3 | Down | 0.74 |
| 250 | Lrpap1 | Alpha-2-macroglobulin receptor-associated protein | P55302 | Down | 0.74 |
| 251 | Rpl10a | 60S ribosomal protein L10a | P53026 | Down | 0.73 |
| 252 | Rps23 | 40S ribosomal protein S23 | P62267 | Down | 0.73 |
| 253 | Fus | RNA-binding protein FUS | P56959 | Down | 0.73 |
| 254 | Tubb6 | Tubulin beta-6 chain | Q922F4 | Down | 0.73 |
| 255 | Fam114a1 | Protein Noxp20 | Q9D281 | Down | 0.72 |
| 256 | Rrbp1 | Ribosome-binding protein 1 | Q99PL5 | Down | 0.72 |
| 257 | Cmpk1 | UMP-CMP kinase | Q9DBP5 | Down | 0.71 |
| 258 | Phgdh | D-3-phosphoglycerate dehydrogenase | Q61753 | Down | 0.71 |
| 259 | Eif4b | Eukaryotic translation initiation factor 4B | Q8BGD9 | Down | 0.71 |
| 260 | Rps5 | 40S ribosomal protein S5 | P97461 | Down | 0.70 |
| 261 | Arf4 | ADP-ribosylation factor 4 | P61750 | Down | 0.70 |
| 262 | P4ha1 | Prolyl 4-hydroxylase subunit alpha-1 | Q60715 | Down | 0.68 |
| 263 | Hnrnpa0 | Heterogeneous nuclear ribonucleoprotein A0 | Q9CX86 | Down | 0.68 |
| 264 | Akap12 | A-kinase anchor protein 12 | Q9WTQ5 | Down | 0.66 |
| 265 | Palm | Paralemmin-1 | Q9Z0P4 | Down | 0.64 |
| 266 | Hdgf | Hepatoma-derived growth factor | P51859 | Down | 0.63 |
| 267 | Fkbp9 | Peptidyl-prolyl cis-trans isomerase FKBP9 | Q9Z247 | Down | 0.63 |
| 268 | Ckap4 | Cytoskeleton-associated protein 4 | Q8BMK4 | Down | 0.61 |
| 269 | Lmnb1 | Lamin-B1 | P14733 | Down | 0.61 |
| 270 | Erp29 | Endoplasmic reticulum resident protein 29 | P57759 | Down | 0.60 |
| 271 | Rcn3 | Reticulocalbin-3 | Q8BH97 | Down | 0.59 |
| 272 | Copz2 | Coatomer subunit zeta-2 | Q9JHH9 | Down | 0.56 |
| 273 | Cnpy2 | Protein canopy homolog 2 | Q9QXT0 | Down | 0.54 |
| 274 | Col5a2 | Collagen alpha-2(V) chain | Q3U962 | Down | 0.52 |
| 275 | Agfg1 | Arf-GAP domain and FG repeat-containing protein 1 | Q8K2K6 | Down | 0.52 |
| 276 | Nucb2 | Nucleobindin-2;Nesfatin-1 | P81117 | Down | 0.51 |
| 277 | Serpinh1 | Serpin H1 | P19324 | Down | 0.48 |
| 278 | Col14a1 | Collagen alpha-1(XIV) chain | Q80X19 | Down | 0.43 |
| 279 | Gpx7 | Glutathione peroxidase 7 | Q99LJ6 | Down | 0.42 |
| 280 | Sparc | SPARC | P07214 | Down | 0.40 |
| 281 | Col3a1 | Collagen alpha-1(III) chain | P08121 | Down | 0.39 |
| 282 | Ppic | Peptidyl-prolyl cis-trans isomerase C | P30412 | Down | 0.37 |
| 283 | Col5a1 | Collagen alpha-1(V) chain | O88207 | Down | 0.35 |
| 284 | Ppp1r14a | Protein phosphatase 1 regulatory subunit 14A | Q91VC7 | Down | 0.32 |
| 285 | As3mt | Arsenite methyltransferase | Q91WU5 | Down | 0.30 |
| 286 | Lox | Protein-lysine 6-oxidase | P28301 | Down | 0.28 |
| 287 | Hist1h2bp | Histone H2B type 1-P | Q8CGP2 | Down | 0.27 |
| 288 | Hist1h1b | Histone H1.5 | P43276 | Down | 0.11 |
| 289 | Hist1h1a | Histone H1.1 | P43275 | Down | 0.10 |

**Table S2.** Ageing markers (respective DEPs) in murine aorta independent of strain and sex of mice. Ratio of ageing (40-week old vs 8-week-old) markers expression in murine aorta of 159 upregulated (ratio ≥ 1.0) as well as 130 downregulated (ratio < 1.0) differentially expressed proteins (DEPs) in E3L.CETP female mice. Presented table was prepared based on proteins that were significantly different between compared groups. Statistics: Student’s t-test. Supplementary Table S2 refers to Figure 6 of the main manuscript.

| **No.** | **Protein ID** | **Protein annotation** | **Accession** | **Regulation** | **Ratio** |
| --- | --- | --- | --- | --- | --- |
| 1 | Igh-3 | Ig gamma-2B chain C region | P01867 | Up | 35.69 |
| 2 | Ighg2a | Ig gamma-2A chain C region secreted form | P01865 | Up | 31.81 |
| 3 | Mgp | Matrix Gla protein | P19788 | Up | 7.79 |
| 4 | IGKC | Ig kappa chain C region | P01834 | Up | 7.33 |
| 5 | Mamdc2 | MAM domain-containing protein 2 | Q8CG85 | Up | 6.96 |
| 6 | Mfge8 | Lactadherin | P21956 | Up | 4.57 |
| 7 | Sdcbp | Syntenin-1 | O08992 | Up | 4.55 |
| 8 | Vtn | Vitronectin | P29788 | Up | 4.45 |
| 9 | Rarres2 | Retinoic acid receptor responder protein 2 | Q9DD06 | Up | 3.98 |
| 10 | Ftl1 | Ferritin light chain 1 | P29391 | Up | 3.72 |
| 11 | Nebl | LIM zinc-binding domain-containing Nebulette | Q9DC07 | Up | 3.68 |
| 12 | Sbspon | Somatomedin-B and thrombospondin type-1 domain-containing protein | Q3UPR9 | Up | 3.65 |
| 13 | Trim47 | Tripartite motif-containing protein 47 | Q8C0E3 | Up | 3.46 |
| 14 | Ighm | Ig mu chain C region | P01872 | Up | 3.43 |
| 15 | Slc29a1 | Equilibrative nucleoside transporter 1 | Q9JIM1 | Up | 3.29 |
| 16 | Htra1 | Serine protease HTRA1 | Q9R118 | Up | 3.22 |
| 17 | Cma1 | Chymase | P21844 | Up | 3.13 |
| 18 | Vcan | Versican core protein | Q62059 | Up | 3.03 |
| 19 | Hapln1 | Hyaluronan and proteoglycan link protein 1 | Q9QUP5 | Up | 2.89 |
| 20 | Axl | Tyrosine-protein kinase receptor UFO | Q00993 | Up | 2.80 |
| 21 | Loxl3 | Lysyl oxidase homolog 3 | Q9Z175 | Up | 2.62 |
| 22 | Apoa4 | Apolipoprotein A-IV | P06728 | Up | 2.57 |
| 23 | Timp3 | Metalloproteinase inhibitor 3 | P39876 | Up | 2.52 |
| 24 | Fth1 | Ferritin heavy chain | P09528 | Up | 2.12 |
| 25 | Clu | Clusterin | Q06890 | Up | 2.09 |
| 26 | Serpinc1 | Antithrombin-III | P32261 | Up | 2.09 |
| 27 | Plg | Plasminogen | P20918 | Up | 2.05 |
| 28 | Ntn1 | Netrin-1 | O09118 | Up | 2.02 |
| 29 | Bcam | Basal cell adhesion molecule | Q9R069 | Up | 1.99 |
| 30 | Aspn | Asporin | Q99MQ4 | Up | 1.98 |
| 31 | Lamb2 | Laminin subunit beta-2 | Q61292 | Up | 1.97 |
| 32 | Atp2a3 | Sarcoplasmic/endoplasmic reticulum calcium ATPase 3 | Q64518 | Up | 1.92 |
| 33 | Itgb5 | Integrin beta-5 | O70309 | Up | 1.89 |
| 34 | Cnn2 | Calponin-2 | Q08093 | Up | 1.89 |
| 35 | Lamc1 | Laminin subunit gamma-1 | P02468 | Up | 1.83 |
| 36 | App | Amyloid beta A4 protein | P12023 | Up | 1.82 |
| 37 | Lama5 | Laminin subunit alpha-5 | Q61001 | Up | 1.82 |
| 38 | Fn1 | Fibronectin;Anastellin | P11276 | Up | 1.78 |
| 39 | Col6a2 | Collagen alpha-2(VI) chain | Q02788 | Up | 1.72 |
| 40 | Acan | Aggrecan core protein | Q61282 | Up | 1.69 |
| 41 | Agrn | Agrin | A2ASQ1 | Up | 1.69 |
| 42 | Col6a1 | Collagen alpha-1(VI) chain | Q04857 | Up | 1.68 |
| 43 | Tinagl1 | Tubulointerstitial nephritis antigen-like | Q99JR5 | Up | 1.66 |
| 44 | Ston1 | Stonin-1 | Q8CDJ8 | Up | 1.65 |
| 45 | Tpm3 | Tropomyosin alpha-3 chain | P21107 | Up | 1.65 |
| 46 | Lama4 | Laminin subunit alpha-4 | P97927 | Up | 1.64 |
| 47 | Aldh9a1 | 4-trimethylaminobutyraldehyde dehydrogenase | Q9JLJ2 | Up | 1.64 |
| 48 | Itgav | Integrin alpha-V | P43406 | Up | 1.64 |
| 49 | Tagln2 | Transgelin-2 | Q9WVA4 | Up | 1.61 |
| 50 | Fhl3 | Four and a half LIM domains protein 3 | Q9R059 | Up | 1.61 |
| 51 | Cd9 | CD9 antigen | P40240 | Up | 1.61 |
| 52 | Prelp | Prolargin | Q9JK53 | Up | 1.58 |
| 53 | Selenbp1 | Selenium-binding protein 1;Selenium-binding protein 2 | P17563 | Up | 1.58 |
| 54 | Bgn | Biglycan | P28653 | Up | 1.57 |
| 55 | Efemp2 | EGF-containing fibulin-like extracellular matrix protein 2 | Q9WVJ9 | Up | 1.54 |
| 56 | Tes | Testin | P47226 | Up | 1.54 |
| 57 | Cfd | Complement factor D | P03953 | Up | 1.54 |
| 58 | Cd200 | OX-2 membrane glycoprotein | O54901 | Up | 1.52 |
| 59 | Mcam | Cell surface glycoprotein MUC18 | Q8R2Y2 | Up | 1.50 |
| 60 | Efemp1 | EGF-containing fibulin-like extracellular matrix protein 1 | Q8BPB5 | Up | 1.50 |
| 61 | Itga3 | Integrin alpha-3 | Q62470 | Up | 1.49 |
| 62 | Cr1l | Complement component receptor 1-like protein | Q64735 | Up | 1.48 |
| 63 | Sorbs2 | Sorbin and SH3 domain-containing protein 2 | Q3UTJ2 | Up | 1.44 |
| 64 | Abhd14b | Alpha/beta hydrolase domain-containing protein 14B | Q8VCR7 | Up | 1.43 |
| 65 | Atp1b3 | Sodium/potassium-transporting ATPase subunit beta-3 | P97370 | Up | 1.42 |
| 66 | Maob | Amine oxidase [flavin-containing] B | Q8BW75 | Up | 1.42 |
| 67 | Itgb1 | Integrin beta-1 | P09055 | Up | 1.41 |
| 68 | Cd109 | CD109 antigen | Q8R422 | Up | 1.41 |
| 69 | Ndrg1 | Protein NDRG1 | Q62433 | Up | 1.40 |
| 70 | Susd2 | Sushi domain-containing protein 2 | Q9DBX3 | Up | 1.40 |
| 71 | Eml2 | Echinoderm microtubule-associated protein-like 2 | Q7TNG5 | Up | 1.40 |
| 72 | Mfap4 | Microfibril-associated glycoprotein 4 | Q9D1H9 | Up | 1.39 |
| 73 | Cbr1 | Carbonyl reductase [NADPH] 1 | P48758 | Up | 1.39 |
| 74 | Entpd1 | Ectonucleoside triphosphate diphosphohydrolase 1 | P55772 | Up | 1.38 |
| 75 | Asah1 | Acid ceramidase | Q9WV54 | Up | 1.38 |
| 76 | Cacna2d1 | Voltage-dependent calcium channel subunit alpha-2/delta-1 | O08532 | Up | 1.37 |
| 77 | Pdcd6 | Programmed cell death protein 6 | P12815 | Up | 1.37 |
| 78 | Gstp1 | Glutathione S-transferase P 1 | P19157 | Up | 1.36 |
| 79 | Serpina1b | Alpha-1-antitrypsin 1-2 | P22599 | Up | 1.36 |
| 80 | Dtna | Dystrobrevin alpha | Q9D2N4 | Up | 1.36 |
| 81 | Col4a2 | Collagen alpha-2(IV) chain;Canstatin | P08122 | Up | 1.36 |
| 82 | Akr1b8 | Aldose reductase-related protein 2 | P45377 | Up | 1.36 |
| 83 | Cbr3 | Carbonyl reductase [NADPH] 3 | Q8K354 | Up | 1.35 |
| 84 | Anxa4 | Annexin A4 | P97429 | Up | 1.35 |
| 85 | Tgfbi | Transforming growth factor-beta-induced protein ig-h3 | P82198 | Up | 1.34 |
| 86 | Serpina1c;Serpina1a | Alpha-1-antitrypsin 1-3;Alpha-1-antitrypsin 1-1 | Q00896 | Up | 1.33 |
| 87 | Park7 | Protein deglycase DJ-1 | Q99LX0 | Up | 1.33 |
| 88 | Rras | Ras-related protein R-Ras | P10833 | Up | 1.33 |
| 89 | Fblim1 | Filamin-binding LIM protein 1 | Q71FD7 | Up | 1.33 |
| 90 | Smtn | Smoothelin | Q921U8 | Up | 1.32 |
| 91 | Cp | Ceruloplasmin | Q61147 | Up | 1.31 |
| 92 | Maoa | Amine oxidase [flavin-containing] A | Q64133 | Up | 1.31 |
| 93 | Acta2 | Actin, aortic smooth muscle | P62737 | Up | 1.30 |
| 94 | Hspg2 | Basement membrane-specific heparan sulfate proteoglycan core protein | Q05793 | Up | 1.30 |
| 95 | Sfxn3 | Sideroflexin-3 | Q91V61 | Up | 1.29 |
| 96 | Glod4 | Glyoxalase domain-containing protein 4 | Q9CPV4 | Up | 1.29 |
| 97 | Slc44a2 | Choline transporter-like protein 2 | Q8BY89 | Up | 1.29 |
| 98 | Gstm1 | Glutathione S-transferase Mu 1 | P10649 | Up | 1.29 |
| 99 | Rab18 | Ras-related protein Rab-18 | P35293 | Up | 1.28 |
| 100 | Col18a1 | Collagen alpha-1(XVIII) chain;Endostatin | P39061 | Up | 1.28 |
| 101 | Cdh13 | Cadherin-13 | Q9WTR5 | Up | 1.28 |
| 102 | Ehd4 | EH domain-containing protein 4 | Q9EQP2 | Up | 1.27 |
| 103 | Lum | Lumican | P51885 | Up | 1.27 |
| 104 | Tpm1 | Tropomyosin alpha-1 chain | P58771 | Up | 1.26 |
| 105 | Pkm | Pyruvate kinase PKM | P52480 | Up | 1.26 |
| 106 | Fbln2 | Fibulin-2 | P37889 | Up | 1.26 |
| 107 | Spr | Sepiapterin reductase | Q64105 | Up | 1.26 |
| 108 | Gstt3 | Glutathione S-transferase theta-3 | Q99L20 | Up | 1.25 |
| 109 | Gapdh | Glyceraldehyde-3-phosphate dehydrogenase | P16858 | Up | 1.25 |
| 110 | Ola1 | Obg-like ATPase 1 | Q9CZ30 | Up | 1.24 |
| 111 | Rnase4 | Ribonuclease 4 | Q9JJH1 | Up | 1.24 |
| 112 | Ilk | Integrin-linked protein kinase | O55222 | Up | 1.24 |
| 113 | Adh5 | Alcohol dehydrogenase class-3 | P28474 | Up | 1.24 |
| 114 | Cyb5r3 | NADH-cytochrome b5 reductase 3 | Q9DCN2 | Up | 1.23 |
| 115 | Tns2 | Tensin-2 | Q8CGB6 | Up | 1.23 |
| 116 | Msn | Moesin | P26041 | Up | 1.23 |
| 117 | Nptn | Neuroplastin | P97300 | Up | 1.23 |
| 118 | Pgm5 | Phosphoglucomutase-like protein 5 | Q8BZF8 | Up | 1.23 |
| 119 | Itga8 | Integrin alpha-8 | A2ARA8 | Up | 1.23 |
| 120 | Gpi | Glucose-6-phosphate isomerase | P06745 | Up | 1.22 |
| 121 | Dmd | Dystrophin | P11531 | Up | 1.22 |
| 122 | Tgm2 | Protein-glutamine gamma-glutamyltransferase 2 | P21981 | Up | 1.22 |
| 123 | Dcn | Decorin | P28654 | Up | 1.22 |
| 124 | Col4a1 | Collagen alpha-1(IV) chain;Arresten | P02463 | Up | 1.22 |
| 125 | Ppia | Peptidyl-prolyl cis-trans isomerase A | P17742 | Up | 1.21 |
| 126 | Nqo1 | NAD(P)H dehydrogenase [quinone] 1 | Q64669 | Up | 1.21 |
| 127 | Pdxk | Pyridoxal kinase | Q8K183 | Up | 1.21 |
| 128 | Inmt | Indolethylamine N-methyltransferase | P40936 | Up | 1.21 |
| 129 | G6pdx | Glucose-6-phosphate 1-dehydrogenase X | Q00612 | Up | 1.20 |
| 130 | Rhoa | Transforming protein RhoA | Q9QUI0 | Up | 1.19 |
| 131 | Csrp1 | Cysteine and glycine-rich protein 1 | P97315 | Up | 1.19 |
| 132 | Camk2g | Calcium/calmodulin-dependent protein kinase type II subunit gamma | Q923T9 | Up | 1.19 |
| 133 | Ephx1 | Epoxide hydrolase 1 | Q9D379 | Up | 1.19 |
| 134 | Entpd2 | Ectonucleoside triphosphate diphosphohydrolase 2 | O55026 | Up | 1.18 |
| 135 | Adh1 | Alcohol dehydrogenase 1 | P00329 | Up | 1.18 |
| 136 | Gstm2 | Glutathione S-transferase Mu 2 | P15626 | Up | 1.18 |
| 137 | Adipoq | Adiponectin | Q60994 | Up | 1.18 |
| 138 | Tln1 | Talin-1 | P26039 | Up | 1.18 |
| 139 | Gyg1 | Glycogenin-1 | Q9R062 | Up | 1.18 |
| 140 | Flna | Filamin-A | Q8BTM8 | Up | 1.18 |
| 141 | Anxa1 | Annexin A1 | P10107 | Up | 1.17 |
| 142 | Gstt1 | Glutathione S-transferase theta-1 | Q64471 | Up | 1.17 |
| 143 | Atic | Bifunctional purine biosynthesis protein PURH | Q9CWJ9 | Up | 1.17 |
| 144 | Fhl1 | Four and a half LIM domains protein 1 | P97447 | Up | 1.17 |
| 145 | Dag1 | Dystroglycan | Q62165 | Up | 1.16 |
| 146 | Pfkp | ATP-dependent 6-phosphofructokinase, platelet type | Q9WUA3 | Up | 1.16 |
| 147 | Blvra | Biliverdin reductase A | Q9CY64 | Up | 1.15 |
| 148 | Flot1 | Flotillin-1 | O08917 | Up | 1.15 |
| 149 | Atp1a1 | Sodium/potassium-transporting ATPase subunit alpha-1 | Q8VDN2 | Up | 1.15 |
| 150 | Got2 | Aspartate aminotransferase, mitochondrial | P05202 | Up | 1.15 |
| 151 | Fermt2 | Fermitin family homolog 2 | Q8CIB5 | Up | 1.15 |
| 152 | Ehd1 | EH domain-containing protein 1 | Q9WVK4 | Up | 1.14 |
| 153 | Aldoa | Fructose-bisphosphate aldolase A | P05064 | Up | 1.13 |
| 154 | Vwf | von Willebrand factor;von Willebrand antigen 2 | Q8CIZ8 | Up | 1.13 |
| 155 | Anxa11 | Annexin A11 | P97384 | Up | 1.12 |
| 156 | Ptgis | Prostacyclin synthase | O35074 | Up | 1.12 |
| 157 | Sntb2 | Beta-2-syntrophin | Q61235 | Up | 1.11 |
| 158 | Gnai2 | Guanine nucleotide-binding protein G(i) subunit alpha-2 | P08752 | Up | 1.11 |
| 159 | Aldh2 | Aldehyde dehydrogenase, mitochondrial | P47738 | Up | 1.09 |
| 160 | Rpn1 | Dolichyl-diphosphooligosaccharide--protein glycosyltransferase subunit 1 | Q91YQ5 | Down | 0.94 |
| 161 | Eprs | Bifunctional glutamate/proline--tRNA ligase | Q8CGC7 | Down | 0.93 |
| 162 | Arcn1 | Coatomer subunit delta | Q5XJY5 | Down | 0.92 |
| 163 | Calu | Calumenin | O35887 | Down | 0.92 |
| 164 | Tmed10 | Transmembrane emp24 domain-containing protein 10 | Q9D1D4 | Down | 0.90 |
| 165 | Copa | Coatomer subunit alpha;Xenin;Proxenin | Q8CIE6 | Down | 0.90 |
| 166 | Uso1 | General vesicular transport factor p115 | Q9Z1Z0 | Down | 0.90 |
| 167 | Hnrnpa3 | Heterogeneous nuclear ribonucleoprotein A3 | Q8BG05 | Down | 0.89 |
| 168 | Copg1 | Coatomer subunit gamma-1 | Q9QZE5 | Down | 0.89 |
| 169 | Hnrnph1 | Heterogeneous nuclear ribonucleoprotein H | O35737 | Down | 0.89 |
| 170 | Hnrnpl | Heterogeneous nuclear ribonucleoprotein L | Q8R081 | Down | 0.89 |
| 171 | Ddx39b | Spliceosome RNA helicase Ddx39b | Q9Z1N5 | Down | 0.89 |
| 172 | Ncl | Nucleolin | P09405 | Down | 0.89 |
| 173 | Hnrnpf | Heterogeneous nuclear ribonucleoprotein F | Q9Z2X1 | Down | 0.89 |
| 174 | Asph | Aspartyl/asparaginyl beta-hydroxylase | Q8BSY0 | Down | 0.89 |
| 175 | Aco1 | Cytoplasmic aconitate hydratase | P28271 | Down | 0.89 |
| 176 | Blmh | Bleomycin hydrolase | Q8R016 | Down | 0.89 |
| 177 | Hsp90b1 | Endoplasmin | P08113 | Down | 0.88 |
| 178 | Rpn2 | Dolichyl-diphosphooligosaccharide--protein glycosyltransferase subunit 2 | Q9DBG6 | Down | 0.88 |
| 179 | Dhx9 | ATP-dependent RNA helicase A | O70133 | Down | 0.88 |
| 180 | Sept9 | Septin-9 | Q80UG5 | Down | 0.88 |
| 181 | Idh1 | Isocitrate dehydrogenase [NADP] cytoplasmic | O88844 | Down | 0.88 |
| 182 | Rpl4 | 60S ribosomal protein L4 | Q9D8E6 | Down | 0.88 |
| 183 | Hnrnpm | Heterogeneous nuclear ribonucleoprotein M | Q9D0E1 | Down | 0.87 |
| 184 | Rbbp7 | Histone-binding protein RBBP7 | Q60973 | Down | 0.87 |
| 185 | Rplp0 | 60S acidic ribosomal protein P0 | P14869 | Down | 0.87 |
| 186 | Cbr2 | Carbonyl reductase [NADPH] 2 | P08074 | Down | 0.86 |
| 187 | Pdia4 | Protein disulfide-isomerase A4 | P08003 | Down | 0.86 |
| 188 | Syncrip | Heterogeneous nuclear ribonucleoprotein Q | Q7TMK9 | Down | 0.86 |
| 189 | Rpl5 | 60S ribosomal protein L5 | P47962 | Down | 0.86 |
| 190 | Rpl31 | 60S ribosomal protein L31 | P62900 | Down | 0.86 |
| 191 | Fam114a1 | Protein Noxp20 | Q9D281 | Down | 0.86 |
| 192 | Rpl12 | 60S ribosomal protein L12 | P35979 | Down | 0.86 |
| 193 | Tkt | Transketolase | P40142 | Down | 0.86 |
| 194 | Lasp1 | LIM and SH3 domain protein 1 | Q61792 | Down | 0.86 |
| 195 | Pabpc1 | Polyadenylate-binding protein 1 | P29341 | Down | 0.85 |
| 196 | Copb1 | Coatomer subunit beta | Q9JIF7 | Down | 0.85 |
| 197 | Khsrp | Far upstream element-binding protein 2 | Q3U0V1 | Down | 0.85 |
| 198 | Pdia3 | Protein disulfide-isomerase A3 | P27773 | Down | 0.85 |
| 199 | Eif4g1 | Eukaryotic translation initiation factor 4 gamma 1 | Q6NZJ6 | Down | 0.85 |
| 200 | Sec13 | Protein SEC13 homolog | Q9D1M0 | Down | 0.85 |
| 201 | Sec22b | Vesicle-trafficking protein SEC22b | O08547 | Down | 0.85 |
| 202 | Cope | Coatomer subunit epsilon | O89079 | Down | 0.85 |
| 203 | Ssb | Lupus La protein homolog | P32067 | Down | 0.85 |
| 204 | Calr | Calreticulin | P14211 | Down | 0.85 |
| 205 | Eef1b | Elongation factor 1-beta | O70251 | Down | 0.84 |
| 206 | Rpl17 | 60S ribosomal protein L17 | Q9CPR4 | Down | 0.84 |
| 207 | Rpl6 | 60S ribosomal protein L6 | P47911 | Down | 0.84 |
| 208 | Hspa5 | 78 kDa glucose-regulated protein | P20029 | Down | 0.84 |
| 209 | Hnrnpa2b1 | Heterogeneous nuclear ribonucleoproteins A2/B1 | O88569 | Down | 0.84 |
| 210 | P4hb | Protein disulfide-isomerase | P09103 | Down | 0.84 |
| 211 | Rps3a | 40S ribosomal protein S3a | P97351 | Down | 0.84 |
| 212 | Txndc5 | Thioredoxin domain-containing protein 5 | Q91W90 | Down | 0.83 |
| 213 | Ybx1 | Nuclease-sensitive element-binding protein 1 | P62960 | Down | 0.83 |
| 214 | Cav2 | Caveolin-2 | Q9WVC3 | Down | 0.83 |
| 215 | Rps5 | 40S ribosomal protein S5 | P97461 | Down | 0.83 |
| 216 | Nid2 | Nidogen-2 | O88322 | Down | 0.82 |
| 217 | Gnb2l1 | Guanine nucleotide-binding protein subunit beta-2-like 1 | P68040 | Down | 0.82 |
| 218 | Hnrnpul2 | Heterogeneous nuclear ribonucleoprotein U-like protein 2 | Q00PI9 | Down | 0.82 |
| 219 | Hnrnpa0 | Heterogeneous nuclear ribonucleoprotein A0 | Q9CX86 | Down | 0.82 |
| 220 | Cdc42bpb | Serine/threonine-protein kinase MRCK beta | Q7TT50 | Down | 0.81 |
| 221 | Rpl10a | 60S ribosomal protein L10a | P53026 | Down | 0.81 |
| 222 | FAM120A | Constitutive coactivator of PPAR-gamma-like protein 1 | Q6A0A9 | Down | 0.80 |
| 223 | Fstl1 | Follistatin-related protein 1 | Q62356 | Down | 0.80 |
| 224 | Rpl11 | 60S ribosomal protein L11 | Q9CXW4 | Down | 0.80 |
| 225 | Serbp1 | Plasminogen activator inhibitor 1 RNA-binding protein | Q9CY58 | Down | 0.80 |
| 226 | Rps12 | 40S ribosomal protein S12 | P63323 | Down | 0.80 |
| 227 | Arf4 | ADP-ribosylation factor 4 | P61750 | Down | 0.80 |
| 228 | Copb2 | Coatomer subunit beta | O55029 | Down | 0.80 |
| 229 | Rps23 | 40S ribosomal protein S23 | P62267 | Down | 0.80 |
| 230 | Erp44 | Endoplasmic reticulum resident protein 44 | Q9D1Q6 | Down | 0.79 |
| 231 | Rps7 | 40S ribosomal protein S7 | P62082 | Down | 0.79 |
| 232 | Rps14 | 40S ribosomal protein S14 | P62264 | Down | 0.79 |
| 233 | Rps20 | 40S ribosomal protein S20 | P60867 | Down | 0.78 |
| 234 | Ppib | Peptidyl-prolyl cis-trans isomerase B | P24369 | Down | 0.78 |
| 235 | Rpl23 | 60S ribosomal protein L23 | P62830 | Down | 0.78 |
| 236 | Fkbp4 | Peptidyl-prolyl cis-trans isomerase FKBP4 | P30416 | Down | 0.78 |
| 237 | Lrpap1 | Alpha-2-macroglobulin receptor-associated protein | P55302 | Down | 0.78 |
| 238 | Hnrnpk | Heterogeneous nuclear ribonucleoprotein K | P61979 | Down | 0.78 |
| 239 | Btf3 | Transcription factor BTF3 | Q64152 | Down | 0.78 |
| 240 | Sf1 | Splicing factor 1 | Q64213 | Down | 0.77 |
| 241 | Hnrnpd | Heterogeneous nuclear ribonucleoprotein D0 | Q60668 | Down | 0.77 |
| 242 | Rps17 | 40S ribosomal protein S17 | P63276 | Down | 0.77 |
| 243 | Trim28 | Transcription intermediary factor 1-beta | Q62318 | Down | 0.76 |
| 244 | Fmod | Fibromodulin | P50608 | Down | 0.76 |
| 245 | Cmpk1 | UMP-CMP kinase | Q9DBP5 | Down | 0.76 |
| 246 | Agfg1 | Arf-GAP domain and FG repeat-containing protein 1 | Q8K2K6 | Down | 0.76 |
| 247 | Nucb2 | Nucleobindin-2;Nesfatin-1 | P81117 | Down | 0.75 |
| 248 | Rps15 | 40S ribosomal protein S15 | P62843 | Down | 0.75 |
| 249 | Tubb6 | Tubulin beta-6 chain | Q922F4 | Down | 0.74 |
| 250 | Rps8 | 40S ribosomal protein S8 | P62242 | Down | 0.74 |
| 251 | Rrbp1 | Ribosome-binding protein 1 | Q99PL5 | Down | 0.74 |
| 252 | Rpl13 | 60S ribosomal protein L13 | P47963 | Down | 0.73 |
| 253 | Hnrnpa1 | Heterogeneous nuclear ribonucleoprotein A1 | P49312 | Down | 0.73 |
| 254 | Eef1d | Elongation factor 1-delta | P57776 | Down | 0.72 |
| 255 | Hdgf | Hepatoma-derived growth factor | P51859 | Down | 0.72 |
| 256 | Fus | RNA-binding protein FUS | P56959 | Down | 0.72 |
| 257 | Akap12 | A-kinase anchor protein 12 | Q9WTQ5 | Down | 0.72 |
| 258 | Eif4b | Eukaryotic translation initiation factor 4B | Q8BGD9 | Down | 0.71 |
| 259 | Rps2 | 40S ribosomal protein S2 | P25444 | Down | 0.71 |
| 260 | Hdlbp | Vigilin | Q8VDJ3 | Down | 0.71 |
| 261 | Rpl8 | 60S ribosomal protein L8 | P62918 | Down | 0.70 |
| 262 | Rpl3 | 60S ribosomal protein L3 | P27659 | Down | 0.70 |
| 263 | Tppp3 | Tubulin polymerization-promoting protein family member 3 | Q9CRB6 | Down | 0.68 |
| 264 | Erp29 | Endoplasmic reticulum resident protein 29 | P57759 | Down | 0.68 |
| 265 | Rpl9 | 60S ribosomal protein L9 | P51410 | Down | 0.67 |
| 266 | Palm | Paralemmin-1 | Q9Z0P4 | Down | 0.66 |
| 267 | Ckap4 | Cytoskeleton-associated protein 4 | Q8BMK4 | Down | 0.66 |
| 268 | Rcn3 | Reticulocalbin-3 | Q8BH97 | Down | 0.65 |
| 269 | Cnpy2 | Protein canopy homolog 2 | Q9QXT0 | Down | 0.64 |
| 270 | Fkbp9 | Peptidyl-prolyl cis-trans isomerase FKBP9 | Q9Z247 | Down | 0.64 |
| 271 | Phgdh | D-3-phosphoglycerate dehydrogenase | Q61753 | Down | 0.63 |
| 272 | P4ha1 | Prolyl 4-hydroxylase subunit alpha-1 | Q60715 | Down | 0.62 |
| 273 | Lmnb1 | Lamin-B1 | P14733 | Down | 0.61 |
| 274 | Serpinh1 | Serpin H1 | P19324 | Down | 0.57 |
| 275 | Col14a1 | Collagen alpha-1(XIV) chain | Q80X19 | Down | 0.56 |
| 276 | Col5a2 | Collagen alpha-2(V) chain | Q3U962 | Down | 0.55 |
| 277 | As3mt | Arsenite methyltransferase | Q91WU5 | Down | 0.54 |
| 278 | H2afv;H2afz | Histone H2A.V | Q3THW5 | Down | 0.52 |
| 279 | Sparc | SPARC | P07214 | Down | 0.49 |
| 280 | Col3a1 | Collagen alpha-1(III) chain | P08121 | Down | 0.48 |
| 281 | Copz2 | Coatomer subunit zeta-2 | Q9JHH9 | Down | 0.47 |
| 282 | Gpx7 | Glutathione peroxidase 7 | Q99LJ6 | Down | 0.42 |
| 283 | Ppp1r14a | Protein phosphatase 1 regulatory subunit 14A | Q91VC7 | Down | 0.40 |
| 284 | Col5a1 | Collagen alpha-1(V) chain | O88207 | Down | 0.37 |
| 285 | Lox | Protein-lysine 6-oxidase | P28301 | Down | 0.29 |
| 286 | Hist1h2bp | Histone H2B type 1-P | Q8CGP2 | Down | 0.21 |
| 287 | Ppic | Peptidyl-prolyl cis-trans isomerase C | P30412 | Down | 0.15 |
| 288 | Hist1h1a | Histone H1.1 | P43275 | Down | 0.13 |
| 289 | Hist1h1b | Histone H1.5 | P43276 | Down | 0.12 |

**Table S3.** Ageing markers (respective DEPs) in murine aorta independent of strain and sex of mice. Ratio of ageing (40-week old vs 8-week-old) markers expression in murine aorta of 159 upregulated (ratio ≥ 1.0) as well as 130 downregulated (ratio < 1.0) differentially expressed proteins (DEPs) in C57BL/6J male mice. Presented table was prepared based on proteins that were significantly different between compared groups. Statistics: Student’s t-test. Supplementary Table S3 refers to Figure 6 of the main manuscript.

| **No.** | **Protein ID** | **Protein annotation** | **Accession** | **Regulation** | **Ratio** |
| --- | --- | --- | --- | --- | --- |
| 1 | IGKC | Ig kappa chain C region | P01834 | Up | 17.16 |
| 2 | Igh-3 | Ig gamma-2B chain C region | P01867 | Up | 16.80 |
| 3 | Ighg2a | Ig gamma-2A chain C region secreted form | P01865 | Up | 16.44 |
| 4 | Nebl | LIM zinc-binding domain-containing Nebulette | Q9DC07 | Up | 10.30 |
| 5 | Mgp | Matrix Gla protein | P19788 | Up | 9.70 |
| 6 | Ighm | Ig mu chain C region | P01872 | Up | 8.94 |
| 7 | Ftl1 | Ferritin light chain 1 | P29391 | Up | 8.14 |
| 8 | Mfge8 | Lactadherin | P21956 | Up | 7.03 |
| 9 | Vtn | Vitronectin | P29788 | Up | 5.22 |
| 10 | Sdcbp | Syntenin-1 | O08992 | Up | 5.04 |
| 11 | Mamdc2 | MAM domain-containing protein 2 | Q8CG85 | Up | 4.52 |
| 12 | Timp3 | Metalloproteinase inhibitor 3 | P39876 | Up | 4.34 |
| 13 | Trim47 | Tripartite motif-containing protein 47 | Q8C0E3 | Up | 4.19 |
| 14 | Rarres2 | Retinoic acid receptor responder protein 2 | Q9DD06 | Up | 3.92 |
| 15 | Hapln1 | Hyaluronan and proteoglycan link protein 1 | Q9QUP5 | Up | 3.84 |
| 16 | Vcan | Versican core protein | Q62059 | Up | 3.65 |
| 17 | Htra1 | Serine protease HTRA1 | Q9R118 | Up | 3.58 |
| 18 | Slc29a1 | Equilibrative nucleoside transporter 1 | Q9JIM1 | Up | 3.48 |
| 19 | Cma1 | Chymase | P21844 | Up | 3.33 |
| 20 | Plg | Plasminogen | P20918 | Up | 2.93 |
| 21 | Axl | Tyrosine-protein kinase receptor UFO | Q00993 | Up | 2.85 |
| 22 | Apoa4 | Apolipoprotein A-IV | P06728 | Up | 2.73 |
| 23 | Fth1 | Ferritin heavy chain | P09528 | Up | 2.54 |
| 24 | Atp2a3 | Sarcoplasmic/endoplasmic reticulum calcium ATPase 3 | Q64518 | Up | 2.48 |
| 25 | Clu | Clusterin | Q06890 | Up | 2.40 |
| 26 | Susd2 | Sushi domain-containing protein 2 | Q9DBX3 | Up | 2.38 |
| 27 | Loxl3 | Lysyl oxidase homolog 3 | Q9Z175 | Up | 2.36 |
| 28 | App | Amyloid beta A4 protein | P12023 | Up | 2.36 |
| 29 | Serpina1b | Alpha-1-antitrypsin 1-2 | P22599 | Up | 2.32 |
| 30 | Fn1 | Fibronectin;Anastellin | P11276 | Up | 2.28 |
| 31 | Aspn | Asporin | Q99MQ4 | Up | 2.12 |
| 32 | Cr1l | Complement component receptor 1-like protein | Q64735 | Up | 2.08 |
| 33 | Sbspon | Somatomedin-B and thrombospondin type-1 domain-containing protein | Q3UPR9 | Up | 2.06 |
| 34 | Tinagl1 | Tubulointerstitial nephritis antigen-like | Q99JR5 | Up | 2.05 |
| 35 | Serpinc1 | Antithrombin-III | P32261 | Up | 1.97 |
| 36 | Ston1 | Stonin-1 | Q8CDJ8 | Up | 1.94 |
| 37 | Rnase4 | Ribonuclease 4 | Q9JJH1 | Up | 1.94 |
| 38 | Bcam | Basal cell adhesion molecule | Q9R069 | Up | 1.93 |
| 39 | Inmt | Indolethylamine N-methyltransferase | P40936 | Up | 1.81 |
| 40 | Ntn1 | Netrin-1 | O09118 | Up | 1.78 |
| 41 | Serpina1c;Serpina1a | Alpha-1-antitrypsin 1-3;Alpha-1-antitrypsin 1-1 | Q00896 | Up | 1.77 |
| 42 | Cfd | Complement factor D | P03953 | Up | 1.71 |
| 43 | Col6a2 | Collagen alpha-2(VI) chain | Q02788 | Up | 1.70 |
| 44 | Lama5 | Laminin subunit alpha-5 | Q61001 | Up | 1.69 |
| 45 | Col6a1 | Collagen alpha-1(VI) chain | Q04857 | Up | 1.69 |
| 46 | Gstm1 | Glutathione S-transferase Mu 1 | P10649 | Up | 1.67 |
| 47 | Cd200 | OX-2 membrane glycoprotein | O54901 | Up | 1.66 |
| 48 | Agrn | Agrin | A2ASQ1 | Up | 1.65 |
| 49 | Itgb5 | Integrin beta-5 | O70309 | Up | 1.65 |
| 50 | Lamb2 | Laminin subunit beta-2 | Q61292 | Up | 1.65 |
| 51 | Lamc1 | Laminin subunit gamma-1 | P02468 | Up | 1.65 |
| 52 | Col4a1 | Collagen alpha-1(IV) chain;Arresten | P02463 | Up | 1.63 |
| 53 | Anxa4 | Annexin A4 | P97429 | Up | 1.62 |
| 54 | Col18a1 | Collagen alpha-1(XVIII) chain;Endostatin | P39061 | Up | 1.61 |
| 55 | Ndrg1 | Protein NDRG1 | Q62433 | Up | 1.59 |
| 56 | Maob | Amine oxidase [flavin-containing] B | Q8BW75 | Up | 1.58 |
| 57 | Tpm3 | Tropomyosin alpha-3 chain | P21107 | Up | 1.58 |
| 58 | Aldoa | Fructose-bisphosphate aldolase A | P05064 | Up | 1.57 |
| 59 | Mfap4 | Microfibril-associated glycoprotein 4 | Q9D1H9 | Up | 1.57 |
| 60 | Itga3 | Integrin alpha-3 | Q62470 | Up | 1.56 |
| 61 | Acan | Aggrecan core protein | Q61282 | Up | 1.54 |
| 62 | Tes | Testin | P47226 | Up | 1.52 |
| 63 | Fbln2 | Fibulin-2 | P37889 | Up | 1.51 |
| 64 | Fhl3 | Four and a half LIM domains protein 3 | Q9R059 | Up | 1.50 |
| 65 | Sorbs2 | Sorbin and SH3 domain-containing protein 2 | Q3UTJ2 | Up | 1.50 |
| 66 | Rras | Ras-related protein R-Ras | P10833 | Up | 1.50 |
| 67 | Prelp | Prolargin | Q9JK53 | Up | 1.49 |
| 68 | G6pdx | Glucose-6-phosphate 1-dehydrogenase X | Q00612 | Up | 1.49 |
| 69 | Itgav | Integrin alpha-V | P43406 | Up | 1.49 |
| 70 | Tagln2 | Transgelin-2 | Q9WVA4 | Up | 1.48 |
| 71 | Lama4 | Laminin subunit alpha-4 | P97927 | Up | 1.46 |
| 72 | Tgm2 | Protein-glutamine gamma-glutamyltransferase 2 | P21981 | Up | 1.45 |
| 73 | Aldh9a1 | 4-trimethylaminobutyraldehyde dehydrogenase | Q9JLJ2 | Up | 1.45 |
| 74 | Col4a2 | Collagen alpha-2(IV) chain;Canstatin | P08122 | Up | 1.44 |
| 75 | Vwf | von Willebrand factor;von Willebrand antigen 2 | Q8CIZ8 | Up | 1.44 |
| 76 | Cd109 | CD109 antigen | Q8R422 | Up | 1.43 |
| 77 | Slc44a2 | Choline transporter-like protein 2 | Q8BY89 | Up | 1.43 |
| 78 | Gpi | Glucose-6-phosphate isomerase | P06745 | Up | 1.43 |
| 79 | Akr1b8 | Aldose reductase-related protein 2 | P45377 | Up | 1.43 |
| 80 | Adh1 | Alcohol dehydrogenase 1 | P00329 | Up | 1.41 |
| 81 | Itgb1 | Integrin beta-1 | P09055 | Up | 1.41 |
| 82 | Entpd1 | Ectonucleoside triphosphate diphosphohydrolase 1 | P55772 | Up | 1.41 |
| 83 | Sfxn3 | Sideroflexin-3 | Q91V61 | Up | 1.41 |
| 84 | Flot1 | Flotillin-1 | O08917 | Up | 1.39 |
| 85 | Selenbp1 | Selenium-binding protein 1;Selenium-binding protein 2 | P17563 | Up | 1.39 |
| 86 | Hspg2 | Basement membrane-specific heparan sulfate proteoglycan core protein | Q05793 | Up | 1.38 |
| 87 | Mcam | Cell surface glycoprotein MUC18 | Q8R2Y2 | Up | 1.38 |
| 88 | Efemp1 | EGF-containing fibulin-like extracellular matrix protein 1 | Q8BPB5 | Up | 1.37 |
| 89 | Cd9 | CD9 antigen | P40240 | Up | 1.37 |
| 90 | Tns2 | Tensin-2 | Q8CGB6 | Up | 1.36 |
| 91 | Cp | Ceruloplasmin | Q61147 | Up | 1.36 |
| 92 | Ola1 | Obg-like ATPase 1 | Q9CZ30 | Up | 1.36 |
| 93 | Nqo1 | NAD(P)H dehydrogenase [quinone] 1 | Q64669 | Up | 1.35 |
| 94 | Park7 | Protein deglycase DJ-1 | Q99LX0 | Up | 1.35 |
| 95 | Smtn | Smoothelin | Q921U8 | Up | 1.34 |
| 96 | Bgn | Biglycan | P28653 | Up | 1.34 |
| 97 | Cbr1 | Carbonyl reductase [NADPH] 1 | P48758 | Up | 1.34 |
| 98 | Sntb2 | Beta-2-syntrophin | Q61235 | Up | 1.34 |
| 99 | Pkm | Pyruvate kinase PKM | P52480 | Up | 1.33 |
| 100 | Efemp2 | EGF-containing fibulin-like extracellular matrix protein 2 | Q9WVJ9 | Up | 1.33 |
| 101 | Itga8 | Integrin alpha-8 | A2ARA8 | Up | 1.32 |
| 102 | Pgm5 | Phosphoglucomutase-like protein 5 | Q8BZF8 | Up | 1.32 |
| 103 | Anxa11 | Annexin A11 | P97384 | Up | 1.32 |
| 104 | Msn | Moesin | P26041 | Up | 1.31 |
| 105 | Gyg1 | Glycogenin-1 | Q9R062 | Up | 1.31 |
| 106 | Tpm1 | Tropomyosin alpha-1 chain | P58771 | Up | 1.31 |
| 107 | Nptn | Neuroplastin | P97300 | Up | 1.31 |
| 108 | Gstp1 | Glutathione S-transferase P 1 | P19157 | Up | 1.31 |
| 109 | Rhoa | Transforming protein RhoA | Q9QUI0 | Up | 1.30 |
| 110 | Spr | Sepiapterin reductase | Q64105 | Up | 1.30 |
| 111 | Acta2 | Actin, aortic smooth muscle | P62737 | Up | 1.30 |
| 112 | Ehd4 | EH domain-containing protein 4 | Q9EQP2 | Up | 1.28 |
| 113 | Fblim1 | Filamin-binding LIM protein 1 | Q71FD7 | Up | 1.28 |
| 114 | Cyb5r3 | NADH-cytochrome b5 reductase 3 | Q9DCN2 | Up | 1.28 |
| 115 | Maoa | Amine oxidase [flavin-containing] A | Q64133 | Up | 1.28 |
| 116 | Abhd14b | Alpha/beta hydrolase domain-containing protein 14B | Q8VCR7 | Up | 1.28 |
| 117 | Csrp1 | Cysteine and glycine-rich protein 1 | P97315 | Up | 1.26 |
| 118 | Lum | Lumican | P51885 | Up | 1.26 |
| 119 | Eml2 | Echinoderm microtubule-associated protein-like 2 | Q7TNG5 | Up | 1.26 |
| 120 | Gapdh | Glyceraldehyde-3-phosphate dehydrogenase | P16858 | Up | 1.25 |
| 121 | Gstm2 | Glutathione S-transferase Mu 2 | P15626 | Up | 1.25 |
| 122 | Asah1 | Acid ceramidase | Q9WV54 | Up | 1.24 |
| 123 | Ilk | Integrin-linked protein kinase | O55222 | Up | 1.24 |
| 124 | Cbr3 | Carbonyl reductase [NADPH] 3 | Q8K354 | Up | 1.24 |
| 125 | Cnn2 | Calponin-2 | Q08093 | Up | 1.23 |
| 126 | Atp1b3 | Sodium/potassium-transporting ATPase subunit beta-3 | P97370 | Up | 1.23 |
| 127 | Adh5 | Alcohol dehydrogenase class-3 | P28474 | Up | 1.23 |
| 128 | Atic | Bifunctional purine biosynthesis protein PURH | Q9CWJ9 | Up | 1.23 |
| 129 | Pdcd6 | Programmed cell death protein 6 | P12815 | Up | 1.22 |
| 130 | Adipoq | Adiponectin | Q60994 | Up | 1.22 |
| 131 | Atp1a1 | Sodium/potassium-transporting ATPase subunit alpha-1 | Q8VDN2 | Up | 1.21 |
| 132 | Ptgis | Prostacyclin synthase | O35074 | Up | 1.21 |
| 133 | Blvra | Biliverdin reductase A | Q9CY64 | Up | 1.20 |
| 134 | Anxa1 | Annexin A1 | P10107 | Up | 1.19 |
| 135 | Fhl1 | Four and a half LIM domains protein 1 | P97447 | Up | 1.19 |
| 136 | Aldh2 | Aldehyde dehydrogenase, mitochondrial | P47738 | Up | 1.19 |
| 137 | Gstt1 | Glutathione S-transferase theta-1 | Q64471 | Up | 1.18 |
| 138 | Dmd | Dystrophin | P11531 | Up | 1.18 |
| 139 | Flna | Filamin-A | Q8BTM8 | Up | 1.18 |
| 140 | Entpd2 | Ectonucleoside triphosphate diphosphohydrolase 2 | O55026 | Up | 1.17 |
| 141 | Dag1 | Dystroglycan | Q62165 | Up | 1.17 |
| 142 | Cdh13 | Cadherin-13 | Q9WTR5 | Up | 1.17 |
| 143 | Fermt2 | Fermitin family homolog 2 | Q8CIB5 | Up | 1.17 |
| 144 | Cacna2d1 | Voltage-dependent calcium channel subunit alpha-2/delta-1 | O08532 | Up | 1.16 |
| 145 | Tgfbi | Transforming growth factor-beta-induced protein ig-h3 | P82198 | Up | 1.16 |
| 146 | Dtna | Dystrobrevin alpha | Q9D2N4 | Up | 1.15 |
| 147 | Ehd1 | EH domain-containing protein 1 | Q9WVK4 | Up | 1.15 |
| 148 | Gstt3 | Glutathione S-transferase theta-3 | Q99L20 | Up | 1.13 |
| 149 | Ephx1 | Epoxide hydrolase 1 | Q9D379 | Up | 1.13 |
| 150 | Tln1 | Talin-1 | P26039 | Up | 1.12 |
| 151 | Got2 | Aspartate aminotransferase, mitochondrial | P05202 | Up | 1.12 |
| 152 | Camk2g | Calcium/calmodulin-dependent protein kinase type II subunit gamma | Q923T9 | Up | 1.12 |
| 153 | Dcn | Decorin | P28654 | Up | 1.11 |
| 154 | Glod4 | Glyoxalase domain-containing protein 4 | Q9CPV4 | Up | 1.11 |
| 155 | Ppia | Peptidyl-prolyl cis-trans isomerase A | P17742 | Up | 1.11 |
| 156 | Pfkp | ATP-dependent 6-phosphofructokinase, platelet type | Q9WUA3 | Up | 1.10 |
| 157 | Gnai2 | Guanine nucleotide-binding protein G(i) subunit alpha-2 | P08752 | Up | 1.10 |
| 158 | Rab18 | Ras-related protein Rab-18 | P35293 | Up | 1.10 |
| 159 | Pdxk | Pyridoxal kinase | Q8K183 | Up | 1.10 |
| 160 | Eprs | Bifunctional glutamate/proline--tRNA ligase | Q8CGC7 | Down | 0.90 |
| 161 | Tkt | Transketolase | P40142 | Down | 0.90 |
| 162 | Pdia4 | Protein disulfide-isomerase A4 | P08003 | Down | 0.89 |
| 163 | Cbr2 | Carbonyl reductase [NADPH] 2 | P08074 | Down | 0.89 |
| 164 | Rps14 | 40S ribosomal protein S14 | P62264 | Down | 0.89 |
| 165 | Khsrp | Far upstream element-binding protein 2 | Q3U0V1 | Down | 0.88 |
| 166 | Idh1 | Isocitrate dehydrogenase [NADP] cytoplasmic | O88844 | Down | 0.87 |
| 167 | Rps2 | 40S ribosomal protein S2 | P25444 | Down | 0.87 |
| 168 | Sept9 | Septin-9 | Q80UG5 | Down | 0.87 |
| 169 | Gnb2l1 | Guanine nucleotide-binding protein subunit beta-2-like 1 | P68040 | Down | 0.86 |
| 170 | Lrpap1 | Alpha-2-macroglobulin receptor-associated protein | P55302 | Down | 0.86 |
| 171 | Rps5 | 40S ribosomal protein S5 | P97461 | Down | 0.86 |
| 172 | Rpl17 | 60S ribosomal protein L17 | Q9CPR4 | Down | 0.86 |
| 173 | Hnrnpf | Heterogeneous nuclear ribonucleoprotein F | Q9Z2X1 | Down | 0.85 |
| 174 | Hnrnpa2b1 | Heterogeneous nuclear ribonucleoproteins A2/B1 | O88569 | Down | 0.85 |
| 175 | Rps20 | 40S ribosomal protein S20 | P60867 | Down | 0.85 |
| 176 | Hnrnph1 | Heterogeneous nuclear ribonucleoprotein H | O35737 | Down | 0.85 |
| 177 | Calu | Calumenin | O35887 | Down | 0.85 |
| 178 | Copg1 | Coatomer subunit gamma-1 | Q9QZE5 | Down | 0.85 |
| 179 | Erp44 | Endoplasmic reticulum resident protein 44 | Q9D1Q6 | Down | 0.84 |
| 180 | Rpl8 | 60S ribosomal protein L8 | P62918 | Down | 0.84 |
| 181 | Hsp90b1 | Endoplasmin | P08113 | Down | 0.83 |
| 182 | Ddx39b | Spliceosome RNA helicase Ddx39b | Q9Z1N5 | Down | 0.83 |
| 183 | Asph | Aspartyl/asparaginyl beta-hydroxylase | Q8BSY0 | Down | 0.83 |
| 184 | Hnrnpk | Heterogeneous nuclear ribonucleoprotein K | P61979 | Down | 0.83 |
| 185 | Uso1 | General vesicular transport factor p115 | Q9Z1Z0 | Down | 0.83 |
| 186 | Hnrnpm | Heterogeneous nuclear ribonucleoprotein M | Q9D0E1 | Down | 0.83 |
| 187 | Rpl4 | 60S ribosomal protein L4 | Q9D8E6 | Down | 0.83 |
| 188 | Hspa5 | 78 kDa glucose-regulated protein | P20029 | Down | 0.83 |
| 189 | Copb2 | Coatomer subunit beta | O55029 | Down | 0.83 |
| 190 | P4hb | Protein disulfide-isomerase | P09103 | Down | 0.83 |
| 191 | Rplp0 | 60S acidic ribosomal protein P0 | P14869 | Down | 0.82 |
| 192 | Eif4g1 | Eukaryotic translation initiation factor 4 gamma 1 | Q6NZJ6 | Down | 0.82 |
| 193 | Arf4 | ADP-ribosylation factor 4 | P61750 | Down | 0.82 |
| 194 | Rps3a | 40S ribosomal protein S3a | P97351 | Down | 0.82 |
| 195 | Rpl5 | 60S ribosomal protein L5 | P47962 | Down | 0.82 |
| 196 | Sec13 | Protein SEC13 homolog | Q9D1M0 | Down | 0.82 |
| 197 | Ncl | Nucleolin | P09405 | Down | 0.82 |
| 198 | Pdia3 | Protein disulfide-isomerase A3 | P27773 | Down | 0.81 |
| 199 | Aco1 | Cytoplasmic aconitate hydratase | P28271 | Down | 0.81 |
| 200 | Rpn1 | Dolichyl-diphosphooligosaccharide--protein glycosyltransferase subunit 1 | Q91YQ5 | Down | 0.81 |
| 201 | Rpl12 | 60S ribosomal protein L12 | P35979 | Down | 0.81 |
| 202 | Ybx1 | Nuclease-sensitive element-binding protein 1 | P62960 | Down | 0.81 |
| 203 | Pabpc1 | Polyadenylate-binding protein 1 | P29341 | Down | 0.81 |
| 204 | Arcn1 | Coatomer subunit delta | Q5XJY5 | Down | 0.81 |
| 205 | Hnrnpl | Heterogeneous nuclear ribonucleoprotein L | Q8R081 | Down | 0.80 |
| 206 | Sf1 | Splicing factor 1 | Q64213 | Down | 0.80 |
| 207 | Rpl9 | 60S ribosomal protein L9 | P51410 | Down | 0.80 |
| 208 | Rps17 | 40S ribosomal protein S17 | P63276 | Down | 0.80 |
| 209 | Blmh | Bleomycin hydrolase | Q8R016 | Down | 0.80 |
| 210 | Cdc42bpb | Serine/threonine-protein kinase MRCK beta | Q7TT50 | Down | 0.79 |
| 211 | Calr | Calreticulin | P14211 | Down | 0.79 |
| 212 | Fkbp4 | Peptidyl-prolyl cis-trans isomerase FKBP4 | P30416 | Down | 0.79 |
| 213 | Tmed10 | Transmembrane emp24 domain-containing protein 10 | Q9D1D4 | Down | 0.79 |
| 214 | Dhx9 | ATP-dependent RNA helicase A | O70133 | Down | 0.79 |
| 215 | Sec22b | Vesicle-trafficking protein SEC22b | O08547 | Down | 0.79 |
| 216 | Copb1 | Coatomer subunit beta | Q9JIF7 | Down | 0.78 |
| 217 | Rpn2 | Dolichyl-diphosphooligosaccharide--protein glycosyltransferase subunit 2 | Q9DBG6 | Down | 0.78 |
| 218 | Rpl3 | 60S ribosomal protein L3 | P27659 | Down | 0.78 |
| 219 | FAM120A | Constitutive coactivator of PPAR-gamma-like protein 1 | Q6A0A9 | Down | 0.78 |
| 220 | Rpl6 | 60S ribosomal protein L6 | P47911 | Down | 0.78 |
| 221 | Rps7 | 40S ribosomal protein S7 | P62082 | Down | 0.78 |
| 222 | Ssb | Lupus La protein homolog | P32067 | Down | 0.78 |
| 223 | Txndc5 | Thioredoxin domain-containing protein 5 | Q91W90 | Down | 0.77 |
| 224 | Hnrnpa3 | Heterogeneous nuclear ribonucleoprotein A3 | Q8BG05 | Down | 0.77 |
| 225 | Copa | Coatomer subunit alpha;Xenin;Proxenin | Q8CIE6 | Down | 0.77 |
| 226 | Rps8 | 40S ribosomal protein S8 | P62242 | Down | 0.77 |
| 227 | Syncrip | Heterogeneous nuclear ribonucleoprotein Q | Q7TMK9 | Down | 0.77 |
| 228 | Eef1d | Elongation factor 1-delta | P57776 | Down | 0.76 |
| 229 | Rpl10a | 60S ribosomal protein L10a | P53026 | Down | 0.76 |
| 230 | Trim28 | Transcription intermediary factor 1-beta | Q62318 | Down | 0.76 |
| 231 | Fmod | Fibromodulin | P50608 | Down | 0.76 |
| 232 | Nid2 | Nidogen-2 | O88322 | Down | 0.75 |
| 233 | Ppib | Peptidyl-prolyl cis-trans isomerase B | P24369 | Down | 0.75 |
| 234 | Rps12 | 40S ribosomal protein S12 | P63323 | Down | 0.75 |
| 235 | Serbp1 | Plasminogen activator inhibitor 1 RNA-binding protein | Q9CY58 | Down | 0.75 |
| 236 | Lasp1 | LIM and SH3 domain protein 1 | Q61792 | Down | 0.75 |
| 237 | Rps15 | 40S ribosomal protein S15 | P62843 | Down | 0.75 |
| 238 | Cope | Coatomer subunit epsilon | O89079 | Down | 0.75 |
| 239 | Rpl31 | 60S ribosomal protein L31 | P62900 | Down | 0.75 |
| 240 | Hnrnpa1 | Heterogeneous nuclear ribonucleoprotein A1 | P49312 | Down | 0.74 |
| 241 | Hnrnpul2 | Heterogeneous nuclear ribonucleoprotein U-like protein 2 | Q00PI9 | Down | 0.74 |
| 242 | Rpl13 | 60S ribosomal protein L13 | P47963 | Down | 0.74 |
| 243 | Hnrnpd | Heterogeneous nuclear ribonucleoprotein D0 | Q60668 | Down | 0.73 |
| 244 | H2afv;H2afz | Histone H2A.V | Q3THW5 | Down | 0.73 |
| 245 | Cav2 | Caveolin-2 | Q9WVC3 | Down | 0.73 |
| 246 | Eef1b | Elongation factor 1-beta | O70251 | Down | 0.72 |
| 247 | Hdlbp | Vigilin | Q8VDJ3 | Down | 0.72 |
| 248 | Fstl1 | Follistatin-related protein 1 | Q62356 | Down | 0.72 |
| 249 | Fus | RNA-binding protein FUS | P56959 | Down | 0.72 |
| 250 | Tubb6 | Tubulin beta-6 chain | Q922F4 | Down | 0.72 |
| 251 | Cnpy2 | Protein canopy homolog 2 | Q9QXT0 | Down | 0.72 |
| 252 | Tppp3 | Tubulin polymerization-promoting protein family member 3 | Q9CRB6 | Down | 0.71 |
| 253 | Rpl11 | 60S ribosomal protein L11 | Q9CXW4 | Down | 0.71 |
| 254 | Rbbp7 | Histone-binding protein RBBP7 | Q60973 | Down | 0.69 |
| 255 | Rrbp1 | Ribosome-binding protein 1 | Q99PL5 | Down | 0.69 |
| 256 | Cmpk1 | UMP-CMP kinase | Q9DBP5 | Down | 0.69 |
| 257 | Erp29 | Endoplasmic reticulum resident protein 29 | P57759 | Down | 0.68 |
| 258 | Rps23 | 40S ribosomal protein S23 | P62267 | Down | 0.68 |
| 259 | Copz2 | Coatomer subunit zeta-2 | Q9JHH9 | Down | 0.68 |
| 260 | Hnrnpa0 | Heterogeneous nuclear ribonucleoprotein A0 | Q9CX86 | Down | 0.66 |
| 261 | Rpl23 | 60S ribosomal protein L23 | P62830 | Down | 0.66 |
| 262 | Phgdh | D-3-phosphoglycerate dehydrogenase | Q61753 | Down | 0.65 |
| 263 | Nucb2 | Nucleobindin-2;Nesfatin-1 | P81117 | Down | 0.64 |
| 264 | Palm | Paralemmin-1 | Q9Z0P4 | Down | 0.63 |
| 265 | Ckap4 | Cytoskeleton-associated protein 4 | Q8BMK4 | Down | 0.63 |
| 266 | Fam114a1 | Protein Noxp20 | Q9D281 | Down | 0.61 |
| 267 | Hdgf | Hepatoma-derived growth factor | P51859 | Down | 0.60 |
| 268 | Lmnb1 | Lamin-B1 | P14733 | Down | 0.58 |
| 269 | Agfg1 | Arf-GAP domain and FG repeat-containing protein 1 | Q8K2K6 | Down | 0.58 |
| 270 | Btf3 | Transcription factor BTF3 | Q64152 | Down | 0.58 |
| 271 | Fkbp9 | Peptidyl-prolyl cis-trans isomerase FKBP9 | Q9Z247 | Down | 0.53 |
| 272 | P4ha1 | Prolyl 4-hydroxylase subunit alpha-1 | Q60715 | Down | 0.51 |
| 273 | Rcn3 | Reticulocalbin-3 | Q8BH97 | Down | 0.50 |
| 274 | As3mt | Arsenite methyltransferase | Q91WU5 | Down | 0.47 |
| 275 | Serpinh1 | Serpin H1 | P19324 | Down | 0.44 |
| 276 | Col5a2 | Collagen alpha-2(V) chain | Q3U962 | Down | 0.43 |
| 277 | Eif4b | Eukaryotic translation initiation factor 4B | Q8BGD9 | Down | 0.43 |
| 278 | Akap12 | A-kinase anchor protein 12 | Q9WTQ5 | Down | 0.43 |
| 279 | Sparc | SPARC | P07214 | Down | 0.37 |
| 280 | Col5a1 | Collagen alpha-1(V) chain | O88207 | Down | 0.33 |
| 281 | Gpx7 | Glutathione peroxidase 7 | Q99LJ6 | Down | 0.33 |
| 282 | Lox | Protein-lysine 6-oxidase | P28301 | Down | 0.31 |
| 283 | Ppp1r14a | Protein phosphatase 1 regulatory subunit 14A | Q91VC7 | Down | 0.31 |
| 284 | Ppic | Peptidyl-prolyl cis-trans isomerase C | P30412 | Down | 0.28 |
| 285 | Col14a1 | Collagen alpha-1(XIV) chain | Q80X19 | Down | 0.25 |
| 286 | Col3a1 | Collagen alpha-1(III) chain | P08121 | Down | 0.22 |
| 287 | Hist1h2bp | Histone H2B type 1-P | Q8CGP2 | Down | 0.20 |
| 288 | Hist1h1a | Histone H1.1 | P43275 | Down | 0.16 |
| 289 | Hist1h1b | Histone H1.5 | P43276 | Down | 0.12 |

**Table S4.** Ageing markers (respective DEPs) in murine aorta independent of strain and sex of mice. Ratio of ageing (40-week old vs 8-week-old) markers expression in murine aorta of 159 upregulated (ratio ≥ 1.0) as well as 130 downregulated (ratio < 1.0) differentially expressed proteins (DEPs) in E3L.CETP male mice. Presented table was prepared based on proteins that were significantly different between compared groups. Statistics: Student’s t-test. Supplementary Table S4 refers to Figure 6 of the main manuscript.

| **No.** | **Protein ID** | **Protein annotation** | **Accession** | **Regulation** | **Ratio** |
| --- | --- | --- | --- | --- | --- |
| 1 | Ighg2a | Ig gamma-2A chain C region secreted form | P01865 | Up | 20.71 |
| 2 | Mgp | Matrix Gla protein | P19788 | Up | 10.54 |
| 3 | Ftl1 | Ferritin light chain 1 | P29391 | Up | 10.05 |
| 4 | Nebl | LIM zinc-binding domain-containing Nebulette | Q9DC07 | Up | 6.28 |
| 5 | Igh-3 | Ig gamma-2B chain C region | P01867 | Up | 6.15 |
| 6 | Mfge8 | Lactadherin | P21956 | Up | 5.21 |
| 7 | Ighm | Ig mu chain C region | P01872 | Up | 4.97 |
| 8 | Cma1 | Chymase | P21844 | Up | 4.59 |
| 9 | Vtn | Vitronectin | P29788 | Up | 3.65 |
| 10 | Trim47 | Tripartite motif-containing protein 47 | Q8C0E3 | Up | 3.59 |
| 11 | Vcan | Versican core protein | Q62059 | Up | 3.55 |
| 12 | Sdcbp | Syntenin-1 | O08992 | Up | 3.51 |
| 13 | Hapln1 | Hyaluronan and proteoglycan link protein 1 | Q9QUP5 | Up | 3.36 |
| 14 | IGKC | Ig kappa chain C region | P01834 | Up | 3.36 |
| 15 | Axl | Tyrosine-protein kinase receptor UFO | Q00993 | Up | 3.17 |
| 16 | Htra1 | Serine protease HTRA1 | Q9R118 | Up | 3.13 |
| 17 | Rarres2 | Retinoic acid receptor responder protein 2 | Q9DD06 | Up | 3.11 |
| 18 | Loxl3 | Lysyl oxidase homolog 3 | Q9Z175 | Up | 3.03 |
| 19 | Timp3 | Metalloproteinase inhibitor 3 | P39876 | Up | 2.98 |
| 20 | Slc29a1 | Equilibrative nucleoside transporter 1 | Q9JIM1 | Up | 2.88 |
| 21 | Cr1l | Complement component receptor 1-like protein | Q64735 | Up | 2.53 |
| 22 | Susd2 | Sushi domain-containing protein 2 | Q9DBX3 | Up | 2.35 |
| 23 | Sbspon | Somatomedin-B and thrombospondin type-1 domain-containing protein | Q3UPR9 | Up | 2.32 |
| 24 | Aspn | Asporin | Q99MQ4 | Up | 2.29 |
| 25 | Serpina1b | Alpha-1-antitrypsin 1-2 | P22599 | Up | 2.20 |
| 26 | Fth1 | Ferritin heavy chain | P09528 | Up | 2.19 |
| 27 | Mamdc2 | MAM domain-containing protein 2 | Q8CG85 | Up | 2.11 |
| 28 | Apoa4 | Apolipoprotein A-IV | P06728 | Up | 2.06 |
| 29 | Tinagl1 | Tubulointerstitial nephritis antigen-like | Q99JR5 | Up | 2.06 |
| 30 | Clu | Clusterin | Q06890 | Up | 1.99 |
| 31 | Bcam | Basal cell adhesion molecule | Q9R069 | Up | 1.97 |
| 32 | App | Amyloid beta A4 protein | P12023 | Up | 1.95 |
| 33 | Fn1 | Fibronectin;Anastellin | P11276 | Up | 1.88 |
| 34 | Anxa4 | Annexin A4 | P97429 | Up | 1.87 |
| 35 | Plg | Plasminogen | P20918 | Up | 1.84 |
| 36 | Itgb5 | Integrin beta-5 | O70309 | Up | 1.84 |
| 37 | Col6a2 | Collagen alpha-2(VI) chain | Q02788 | Up | 1.80 |
| 38 | Acan | Aggrecan core protein | Q61282 | Up | 1.80 |
| 39 | Atp2a3 | Sarcoplasmic/endoplasmic reticulum calcium ATPase 3 | Q64518 | Up | 1.79 |
| 40 | Ntn1 | Netrin-1 | O09118 | Up | 1.77 |
| 41 | Serpinc1 | Antithrombin-III | P32261 | Up | 1.76 |
| 42 | Itga3 | Integrin alpha-3 | Q62470 | Up | 1.76 |
| 43 | Lama5 | Laminin subunit alpha-5 | Q61001 | Up | 1.75 |
| 44 | Tagln2 | Transgelin-2 | Q9WVA4 | Up | 1.74 |
| 45 | Lamb2 | Laminin subunit beta-2 | Q61292 | Up | 1.74 |
| 46 | Mfap4 | Microfibril-associated glycoprotein 4 | Q9D1H9 | Up | 1.70 |
| 47 | Col6a1 | Collagen alpha-1(VI) chain | Q04857 | Up | 1.70 |
| 48 | Gstm1 | Glutathione S-transferase Mu 1 | P10649 | Up | 1.68 |
| 49 | Lamc1 | Laminin subunit gamma-1 | P02468 | Up | 1.65 |
| 50 | Rnase4 | Ribonuclease 4 | Q9JJH1 | Up | 1.63 |
| 51 | Fbln2 | Fibulin-2 | P37889 | Up | 1.63 |
| 52 | Col4a1 | Collagen alpha-1(IV) chain;Arresten | P02463 | Up | 1.61 |
| 53 | Efemp1 | EGF-containing fibulin-like extracellular matrix protein 1 | Q8BPB5 | Up | 1.60 |
| 54 | Ston1 | Stonin-1 | Q8CDJ8 | Up | 1.60 |
| 55 | Inmt | Indolethylamine N-methyltransferase | P40936 | Up | 1.60 |
| 56 | Ndrg1 | Protein NDRG1 | Q62433 | Up | 1.57 |
| 57 | Sfxn3 | Sideroflexin-3 | Q91V61 | Up | 1.57 |
| 58 | Nptn | Neuroplastin | P97300 | Up | 1.57 |
| 59 | Tpm3 | Tropomyosin alpha-3 chain | P21107 | Up | 1.57 |
| 60 | Sorbs2 | Sorbin and SH3 domain-containing protein 2 | Q3UTJ2 | Up | 1.56 |
| 61 | Rras | Ras-related protein R-Ras | P10833 | Up | 1.55 |
| 62 | Itgav | Integrin alpha-V | P43406 | Up | 1.52 |
| 63 | Maob | Amine oxidase [flavin-containing] B | Q8BW75 | Up | 1.51 |
| 64 | Lama4 | Laminin subunit alpha-4 | P97927 | Up | 1.47 |
| 65 | Pdcd6 | Programmed cell death protein 6 | P12815 | Up | 1.47 |
| 66 | Col4a2 | Collagen alpha-2(IV) chain;Canstatin | P08122 | Up | 1.47 |
| 67 | Flot1 | Flotillin-1 | O08917 | Up | 1.46 |
| 68 | Agrn | Agrin | A2ASQ1 | Up | 1.46 |
| 69 | G6pdx | Glucose-6-phosphate 1-dehydrogenase X | Q00612 | Up | 1.45 |
| 70 | Vwf | von Willebrand factor;von Willebrand antigen 2 | Q8CIZ8 | Up | 1.45 |
| 71 | Prelp | Prolargin | Q9JK53 | Up | 1.43 |
| 72 | Fhl3 | Four and a half LIM domains protein 3 | Q9R059 | Up | 1.43 |
| 73 | Col18a1 | Collagen alpha-1(XVIII) chain;Endostatin | P39061 | Up | 1.42 |
| 74 | Itgb1 | Integrin beta-1 | P09055 | Up | 1.41 |
| 75 | Abhd14b | Alpha/beta hydrolase domain-containing protein 14B | Q8VCR7 | Up | 1.41 |
| 76 | Smtn | Smoothelin | Q921U8 | Up | 1.41 |
| 77 | Aldoa | Fructose-bisphosphate aldolase A | P05064 | Up | 1.41 |
| 78 | Atic | Bifunctional purine biosynthesis protein PURH | Q9CWJ9 | Up | 1.41 |
| 79 | Cd109 | CD109 antigen | Q8R422 | Up | 1.40 |
| 80 | Tes | Testin | P47226 | Up | 1.39 |
| 81 | Sntb2 | Beta-2-syntrophin | Q61235 | Up | 1.38 |
| 82 | Tgm2 | Protein-glutamine gamma-glutamyltransferase 2 | P21981 | Up | 1.38 |
| 83 | Pkm | Pyruvate kinase PKM | P52480 | Up | 1.37 |
| 84 | Ilk | Integrin-linked protein kinase | O55222 | Up | 1.37 |
| 85 | Adipoq | Adiponectin | Q60994 | Up | 1.36 |
| 86 | Selenbp1 | Selenium-binding protein 1;Selenium-binding protein 2 | P17563 | Up | 1.35 |
| 87 | Cyb5r3 | NADH-cytochrome b5 reductase 3 | Q9DCN2 | Up | 1.35 |
| 88 | Akr1b8 | Aldose reductase-related protein 2 | P45377 | Up | 1.34 |
| 89 | Aldh9a1 | 4-trimethylaminobutyraldehyde dehydrogenase | Q9JLJ2 | Up | 1.34 |
| 90 | Atp1b3 | Sodium/potassium-transporting ATPase subunit beta-3 | P97370 | Up | 1.34 |
| 91 | Mcam | Cell surface glycoprotein MUC18 | Q8R2Y2 | Up | 1.34 |
| 92 | Entpd2 | Ectonucleoside triphosphate diphosphohydrolase 2 | O55026 | Up | 1.34 |
| 93 | Cd9 | CD9 antigen | P40240 | Up | 1.34 |
| 94 | Efemp2 | EGF-containing fibulin-like extracellular matrix protein 2 | Q9WVJ9 | Up | 1.33 |
| 95 | Cd200 | OX-2 membrane glycoprotein | O54901 | Up | 1.32 |
| 96 | Acta2 | Actin, aortic smooth muscle | P62737 | Up | 1.32 |
| 97 | Gpi | Glucose-6-phosphate isomerase | P06745 | Up | 1.32 |
| 98 | Hspg2 | Basement membrane-specific heparan sulfate proteoglycan core protein | Q05793 | Up | 1.32 |
| 99 | Pgm5 | Phosphoglucomutase-like protein 5 | Q8BZF8 | Up | 1.31 |
| 100 | Gstp1 | Glutathione S-transferase P 1 | P19157 | Up | 1.31 |
| 101 | Rhoa | Transforming protein RhoA | Q9QUI0 | Up | 1.31 |
| 102 | Blvra | Biliverdin reductase A | Q9CY64 | Up | 1.31 |
| 103 | Maoa | Amine oxidase [flavin-containing] A | Q64133 | Up | 1.30 |
| 104 | Cp | Ceruloplasmin | Q61147 | Up | 1.30 |
| 105 | Cbr1 | Carbonyl reductase [NADPH] 1 | P48758 | Up | 1.29 |
| 106 | Gstt3 | Glutathione S-transferase theta-3 | Q99L20 | Up | 1.29 |
| 107 | Spr | Sepiapterin reductase | Q64105 | Up | 1.29 |
| 108 | Anxa11 | Annexin A11 | P97384 | Up | 1.28 |
| 109 | Cdh13 | Cadherin-13 | Q9WTR5 | Up | 1.28 |
| 110 | Cbr3 | Carbonyl reductase [NADPH] 3 | Q8K354 | Up | 1.28 |
| 111 | Slc44a2 | Choline transporter-like protein 2 | Q8BY89 | Up | 1.28 |
| 112 | Ephx1 | Epoxide hydrolase 1 | Q9D379 | Up | 1.28 |
| 113 | Bgn | Biglycan | P28653 | Up | 1.28 |
| 114 | Tns2 | Tensin-2 | Q8CGB6 | Up | 1.27 |
| 115 | Gyg1 | Glycogenin-1 | Q9R062 | Up | 1.27 |
| 116 | Ehd4 | EH domain-containing protein 4 | Q9EQP2 | Up | 1.27 |
| 117 | Gstm2 | Glutathione S-transferase Mu 2 | P15626 | Up | 1.27 |
| 118 | Ola1 | Obg-like ATPase 1 | Q9CZ30 | Up | 1.26 |
| 119 | Cfd | Complement factor D | P03953 | Up | 1.26 |
| 120 | Entpd1 | Ectonucleoside triphosphate diphosphohydrolase 1 | P55772 | Up | 1.26 |
| 121 | Eml2 | Echinoderm microtubule-associated protein-like 2 | Q7TNG5 | Up | 1.25 |
| 122 | Serpina1c;Serpina1a | Alpha-1-antitrypsin 1-3;Alpha-1-antitrypsin 1-1 | Q00896 | Up | 1.25 |
| 123 | Dtna | Dystrobrevin alpha | Q9D2N4 | Up | 1.25 |
| 124 | Tgfbi | Transforming growth factor-beta-induced protein ig-h3 | P82198 | Up | 1.24 |
| 125 | Fblim1 | Filamin-binding LIM protein 1 | Q71FD7 | Up | 1.23 |
| 126 | Cnn2 | Calponin-2 | Q08093 | Up | 1.22 |
| 127 | Itga8 | Integrin alpha-8 | A2ARA8 | Up | 1.22 |
| 128 | Ptgis | Prostacyclin synthase | O35074 | Up | 1.22 |
| 129 | Adh5 | Alcohol dehydrogenase class-3 | P28474 | Up | 1.22 |
| 130 | Adh1 | Alcohol dehydrogenase 1 | P00329 | Up | 1.22 |
| 131 | Lum | Lumican | P51885 | Up | 1.22 |
| 132 | Tpm1 | Tropomyosin alpha-1 chain | P58771 | Up | 1.21 |
| 133 | Cacna2d1 | Voltage-dependent calcium channel subunit alpha-2/delta-1 | O08532 | Up | 1.21 |
| 134 | Ehd1 | EH domain-containing protein 1 | Q9WVK4 | Up | 1.21 |
| 135 | Gstt1 | Glutathione S-transferase theta-1 | Q64471 | Up | 1.21 |
| 136 | Csrp1 | Cysteine and glycine-rich protein 1 | P97315 | Up | 1.20 |
| 137 | Aldh2 | Aldehyde dehydrogenase, mitochondrial | P47738 | Up | 1.19 |
| 138 | Park7 | Protein deglycase DJ-1 | Q99LX0 | Up | 1.19 |
| 139 | Gapdh | Glyceraldehyde-3-phosphate dehydrogenase | P16858 | Up | 1.19 |
| 140 | Camk2g | Calcium/calmodulin-dependent protein kinase type II subunit gamma | Q923T9 | Up | 1.18 |
| 141 | Asah1 | Acid ceramidase | Q9WV54 | Up | 1.18 |
| 142 | Rab18 | Ras-related protein Rab-18 | P35293 | Up | 1.18 |
| 143 | Msn | Moesin | P26041 | Up | 1.18 |
| 144 | Flna | Filamin-A | Q8BTM8 | Up | 1.18 |
| 145 | Dmd | Dystrophin | P11531 | Up | 1.17 |
| 146 | Dcn | Decorin | P28654 | Up | 1.17 |
| 147 | Gnai2 | Guanine nucleotide-binding protein G(i) subunit alpha-2 | P08752 | Up | 1.17 |
| 148 | Fhl1 | Four and a half LIM domains protein 1 | P97447 | Up | 1.17 |
| 149 | Nqo1 | NAD(P)H dehydrogenase [quinone] 1 | Q64669 | Up | 1.17 |
| 150 | Atp1a1 | Sodium/potassium-transporting ATPase subunit alpha-1 | Q8VDN2 | Up | 1.15 |
| 151 | Ppia | Peptidyl-prolyl cis-trans isomerase A | P17742 | Up | 1.15 |
| 152 | Got2 | Aspartate aminotransferase, mitochondrial | P05202 | Up | 1.15 |
| 153 | Anxa1 | Annexin A1 | P10107 | Up | 1.14 |
| 154 | Dag1 | Dystroglycan | Q62165 | Up | 1.13 |
| 155 | Pfkp | ATP-dependent 6-phosphofructokinase, platelet type | Q9WUA3 | Up | 1.12 |
| 156 | Tln1 | Talin-1 | P26039 | Up | 1.12 |
| 157 | Fermt2 | Fermitin family homolog 2 | Q8CIB5 | Up | 1.12 |
| 158 | Glod4 | Glyoxalase domain-containing protein 4 | Q9CPV4 | Up | 1.11 |
| 159 | Pdxk | Pyridoxal kinase | Q8K183 | Up | 1.10 |
| 160 | Sept9 | Septin-9 | Q80UG5 | Down | 0.90 |
| 161 | Uso1 | General vesicular transport factor p115 | Q9Z1Z0 | Down | 0.89 |
| 162 | Hnrnpk | Heterogeneous nuclear ribonucleoprotein K | P61979 | Down | 0.87 |
| 163 | Cdc42bpb | Serine/threonine-protein kinase MRCK beta | Q7TT50 | Down | 0.87 |
| 164 | Eef1d | Elongation factor 1-delta | P57776 | Down | 0.87 |
| 165 | Pabpc1 | Polyadenylate-binding protein 1 | P29341 | Down | 0.87 |
| 166 | Tkt | Transketolase | P40142 | Down | 0.87 |
| 167 | Ddx39b | Spliceosome RNA helicase Ddx39b | Q9Z1N5 | Down | 0.87 |
| 168 | Rpl3 | 60S ribosomal protein L3 | P27659 | Down | 0.87 |
| 169 | Hnrnph1 | Heterogeneous nuclear ribonucleoprotein H | O35737 | Down | 0.86 |
| 170 | Eprs | Bifunctional glutamate/proline--tRNA ligase | Q8CGC7 | Down | 0.86 |
| 171 | Copa | Coatomer subunit alpha;Xenin;Proxenin | Q8CIE6 | Down | 0.86 |
| 172 | Cav2 | Caveolin-2 | Q9WVC3 | Down | 0.86 |
| 173 | Aco1 | Cytoplasmic aconitate hydratase | P28271 | Down | 0.86 |
| 174 | Idh1 | Isocitrate dehydrogenase [NADP] cytoplasmic | O88844 | Down | 0.86 |
| 175 | Pdia4 | Protein disulfide-isomerase A4 | P08003 | Down | 0.86 |
| 176 | FAM120A | Constitutive coactivator of PPAR-gamma-like protein 1 | Q6A0A9 | Down | 0.86 |
| 177 | Asph | Aspartyl/asparaginyl beta-hydroxylase | Q8BSY0 | Down | 0.85 |
| 178 | Calu | Calumenin | O35887 | Down | 0.85 |
| 179 | Rpl17 | 60S ribosomal protein L17 | Q9CPR4 | Down | 0.85 |
| 180 | Rps8 | 40S ribosomal protein S8 | P62242 | Down | 0.85 |
| 181 | Gnb2l1 | Guanine nucleotide-binding protein subunit beta-2-like 1 | P68040 | Down | 0.85 |
| 182 | Nid2 | Nidogen-2 | O88322 | Down | 0.84 |
| 183 | Ncl | Nucleolin | P09405 | Down | 0.84 |
| 184 | Rpl4 | 60S ribosomal protein L4 | Q9D8E6 | Down | 0.84 |
| 185 | Rpn1 | Dolichyl-diphosphooligosaccharide--protein glycosyltransferase subunit 1 | Q91YQ5 | Down | 0.84 |
| 186 | Hnrnpl | Heterogeneous nuclear ribonucleoprotein L | Q8R081 | Down | 0.84 |
| 187 | Fstl1 | Follistatin-related protein 1 | Q62356 | Down | 0.84 |
| 188 | Rpl6 | 60S ribosomal protein L6 | P47911 | Down | 0.84 |
| 189 | Sec13 | Protein SEC13 homolog | Q9D1M0 | Down | 0.84 |
| 190 | Hnrnpm | Heterogeneous nuclear ribonucleoprotein M | Q9D0E1 | Down | 0.84 |
| 191 | Rpl8 | 60S ribosomal protein L8 | P62918 | Down | 0.83 |
| 192 | Rps20 | 40S ribosomal protein S20 | P60867 | Down | 0.83 |
| 193 | Cbr2 | Carbonyl reductase [NADPH] 2 | P08074 | Down | 0.83 |
| 194 | Hnrnpa3 | Heterogeneous nuclear ribonucleoprotein A3 | Q8BG05 | Down | 0.83 |
| 195 | H2afv;H2afz | Histone H2A.V | Q3THW5 | Down | 0.83 |
| 196 | Rpl5 | 60S ribosomal protein L5 | P47962 | Down | 0.82 |
| 197 | Hnrnpf | Heterogeneous nuclear ribonucleoprotein F | Q9Z2X1 | Down | 0.82 |
| 198 | Khsrp | Far upstream element-binding protein 2 | Q3U0V1 | Down | 0.82 |
| 199 | Blmh | Bleomycin hydrolase | Q8R016 | Down | 0.82 |
| 200 | P4hb | Protein disulfide-isomerase | P09103 | Down | 0.82 |
| 201 | Erp44 | Endoplasmic reticulum resident protein 44 | Q9D1Q6 | Down | 0.82 |
| 202 | Txndc5 | Thioredoxin domain-containing protein 5 | Q91W90 | Down | 0.82 |
| 203 | Rplp0 | 60S acidic ribosomal protein P0 | P14869 | Down | 0.82 |
| 204 | Arcn1 | Coatomer subunit delta | Q5XJY5 | Down | 0.81 |
| 205 | Palm | Paralemmin-1 | Q9Z0P4 | Down | 0.81 |
| 206 | Ssb | Lupus La protein homolog | P32067 | Down | 0.81 |
| 207 | Rpn2 | Dolichyl-diphosphooligosaccharide--protein glycosyltransferase subunit 2 | Q9DBG6 | Down | 0.81 |
| 208 | Hnrnpd | Heterogeneous nuclear ribonucleoprotein D0 | Q60668 | Down | 0.81 |
| 209 | Tmed10 | Transmembrane emp24 domain-containing protein 10 | Q9D1D4 | Down | 0.80 |
| 210 | Ybx1 | Nuclease-sensitive element-binding protein 1 | P62960 | Down | 0.80 |
| 211 | Lasp1 | LIM and SH3 domain protein 1 | Q61792 | Down | 0.80 |
| 212 | Hnrnpa2b1 | Heterogeneous nuclear ribonucleoproteins A2/B1 | O88569 | Down | 0.80 |
| 213 | Rpl9 | 60S ribosomal protein L9 | P51410 | Down | 0.80 |
| 214 | Eef1b | Elongation factor 1-beta | O70251 | Down | 0.80 |
| 215 | Calr | Calreticulin | P14211 | Down | 0.79 |
| 216 | Rps7 | 40S ribosomal protein S7 | P62082 | Down | 0.79 |
| 217 | Copg1 | Coatomer subunit gamma-1 | Q9QZE5 | Down | 0.79 |
| 218 | Fus | RNA-binding protein FUS | P56959 | Down | 0.79 |
| 219 | Rbbp7 | Histone-binding protein RBBP7 | Q60973 | Down | 0.79 |
| 220 | Copb1 | Coatomer subunit beta | Q9JIF7 | Down | 0.79 |
| 221 | Rpl11 | 60S ribosomal protein L11 | Q9CXW4 | Down | 0.79 |
| 222 | Rps3a | 40S ribosomal protein S3a | P97351 | Down | 0.79 |
| 223 | Ppib | Peptidyl-prolyl cis-trans isomerase B | P24369 | Down | 0.79 |
| 224 | Sec22b | Vesicle-trafficking protein SEC22b | O08547 | Down | 0.79 |
| 225 | Cope | Coatomer subunit epsilon | O89079 | Down | 0.78 |
| 226 | Rps2 | 40S ribosomal protein S2 | P25444 | Down | 0.78 |
| 227 | Dhx9 | ATP-dependent RNA helicase A | O70133 | Down | 0.78 |
| 228 | Rps17 | 40S ribosomal protein S17 | P63276 | Down | 0.78 |
| 229 | Rpl13 | 60S ribosomal protein L13 | P47963 | Down | 0.78 |
| 230 | Lrpap1 | Alpha-2-macroglobulin receptor-associated protein | P55302 | Down | 0.78 |
| 231 | Arf4 | ADP-ribosylation factor 4 | P61750 | Down | 0.78 |
| 232 | Copb2 | Coatomer subunit beta | O55029 | Down | 0.78 |
| 233 | Hnrnpul2 | Heterogeneous nuclear ribonucleoprotein U-like protein 2 | Q00PI9 | Down | 0.77 |
| 234 | Hsp90b1 | Endoplasmin | P08113 | Down | 0.77 |
| 235 | Rps14 | 40S ribosomal protein S14 | P62264 | Down | 0.77 |
| 236 | Rps5 | 40S ribosomal protein S5 | P97461 | Down | 0.77 |
| 237 | Pdia3 | Protein disulfide-isomerase A3 | P27773 | Down | 0.77 |
| 238 | Tppp3 | Tubulin polymerization-promoting protein family member 3 | Q9CRB6 | Down | 0.77 |
| 239 | Hnrnpa0 | Heterogeneous nuclear ribonucleoprotein A0 | Q9CX86 | Down | 0.76 |
| 240 | Syncrip | Heterogeneous nuclear ribonucleoprotein Q | Q7TMK9 | Down | 0.76 |
| 241 | Sf1 | Splicing factor 1 | Q64213 | Down | 0.76 |
| 242 | Rpl12 | 60S ribosomal protein L12 | P35979 | Down | 0.76 |
| 243 | Rps15 | 40S ribosomal protein S15 | P62843 | Down | 0.76 |
| 244 | Eif4g1 | Eukaryotic translation initiation factor 4 gamma 1 | Q6NZJ6 | Down | 0.75 |
| 245 | Erp29 | Endoplasmic reticulum resident protein 29 | P57759 | Down | 0.75 |
| 246 | Hspa5 | 78 kDa glucose-regulated protein | P20029 | Down | 0.75 |
| 247 | Fkbp4 | Peptidyl-prolyl cis-trans isomerase FKBP4 | P30416 | Down | 0.75 |
| 248 | Hnrnpa1 | Heterogeneous nuclear ribonucleoprotein A1 | P49312 | Down | 0.75 |
| 249 | Rpl10a | 60S ribosomal protein L10a | P53026 | Down | 0.74 |
| 250 | Serbp1 | Plasminogen activator inhibitor 1 RNA-binding protein | Q9CY58 | Down | 0.74 |
| 251 | Rpl31 | 60S ribosomal protein L31 | P62900 | Down | 0.73 |
| 252 | Trim28 | Transcription intermediary factor 1-beta | Q62318 | Down | 0.72 |
| 253 | Rpl23 | 60S ribosomal protein L23 | P62830 | Down | 0.72 |
| 254 | Rps12 | 40S ribosomal protein S12 | P63323 | Down | 0.72 |
| 255 | Fmod | Fibromodulin | P50608 | Down | 0.70 |
| 256 | Hdgf | Hepatoma-derived growth factor | P51859 | Down | 0.69 |
| 257 | Rrbp1 | Ribosome-binding protein 1 | Q99PL5 | Down | 0.69 |
| 258 | Tubb6 | Tubulin beta-6 chain | Q922F4 | Down | 0.69 |
| 259 | Cnpy2 | Protein canopy homolog 2 | Q9QXT0 | Down | 0.68 |
| 260 | Cmpk1 | UMP-CMP kinase | Q9DBP5 | Down | 0.68 |
| 261 | Hdlbp | Vigilin | Q8VDJ3 | Down | 0.66 |
| 262 | Fam114a1 | Protein Noxp20 | Q9D281 | Down | 0.63 |
| 263 | Btf3 | Transcription factor BTF3 | Q64152 | Down | 0.62 |
| 264 | Ckap4 | Cytoskeleton-associated protein 4 | Q8BMK4 | Down | 0.62 |
| 265 | Copz2 | Coatomer subunit zeta-2 | Q9JHH9 | Down | 0.60 |
| 266 | Lmnb1 | Lamin-B1 | P14733 | Down | 0.59 |
| 267 | Rcn3 | Reticulocalbin-3 | Q8BH97 | Down | 0.58 |
| 268 | Phgdh | D-3-phosphoglycerate dehydrogenase | Q61753 | Down | 0.58 |
| 269 | Nucb2 | Nucleobindin-2;Nesfatin-1 | P81117 | Down | 0.56 |
| 270 | P4ha1 | Prolyl 4-hydroxylase subunit alpha-1 | Q60715 | Down | 0.55 |
| 271 | Col5a2 | Collagen alpha-2(V) chain | Q3U962 | Down | 0.54 |
| 272 | As3mt | Arsenite methyltransferase | Q91WU5 | Down | 0.53 |
| 273 | Fkbp9 | Peptidyl-prolyl cis-trans isomerase FKBP9 | Q9Z247 | Down | 0.53 |
| 274 | Akap12 | A-kinase anchor protein 12 | Q9WTQ5 | Down | 0.52 |
| 275 | Rps23 | 40S ribosomal protein S23 | P62267 | Down | 0.51 |
| 276 | Agfg1 | Arf-GAP domain and FG repeat-containing protein 1 | Q8K2K6 | Down | 0.50 |
| 277 | Serpinh1 | Serpin H1 | P19324 | Down | 0.49 |
| 278 | Eif4b | Eukaryotic translation initiation factor 4B | Q8BGD9 | Down | 0.47 |
| 279 | Ppp1r14a | Protein phosphatase 1 regulatory subunit 14A | Q91VC7 | Down | 0.45 |
| 280 | Sparc | SPARC | P07214 | Down | 0.40 |
| 281 | Gpx7 | Glutathione peroxidase 7 | Q99LJ6 | Down | 0.39 |
| 282 | Lox | Protein-lysine 6-oxidase | P28301 | Down | 0.38 |
| 283 | Col14a1 | Collagen alpha-1(XIV) chain | Q80X19 | Down | 0.32 |
| 284 | Col5a1 | Collagen alpha-1(V) chain | O88207 | Down | 0.32 |
| 285 | Col3a1 | Collagen alpha-1(III) chain | P08121 | Down | 0.31 |
| 286 | Hist1h2bp | Histone H2B type 1-P | Q8CGP2 | Down | 0.30 |
| 287 | Ppic | Peptidyl-prolyl cis-trans isomerase C | P30412 | Down | 0.21 |
| 288 | Hist1h1b | Histone H1.5 | P43276 | Down | 0.15 |
| 289 | Hist1h1a | Histone H1.1 | P43275 | Down | 0.12 |

**Table S5.** Characterisation of the early stage of the hyperlipidemia in plasma of 8-week-old mice, based on expression ratio of hyperlipidaemia (E3L.CETP vs C57BL/6J) markers (respective DEPs; upregulated: ratio ≥ 1.0, downregulated: ratio < 1.0) dependent on age and specific for sex in murine plasma of 8-week-old male mice**.** Presented table was prepared based on proteins that were significantly different between compared groups. Statistics: Student’s t-test. Supplementary Table S5 refers to Figure 8 of the main manuscript.

| **No.** | **Protein ID** | **Protein annotation** | **Accession** | **Regulation** | **Ratio** |
| --- | --- | --- | --- | --- | --- |
| 1 | Pgam2 | Phosphoglycerate mutase 2 | O70250 | Up | 17.77 |
| 2 | Asl | Argininosuccinate lyase | Q91YI0 | Up | 15.61 |
| 3 | Selenbp2 | Selenium-binding protein 2 | Q63836 | Up | 13.61 |
| 4 | Pnp | Purine nucleoside phosphorylase | P23492 | Up | 12.88 |
| 5 | Prdx6 | Peroxiredoxin-6 | O08709 | Up | 12.74 |
| 6 | Bpgm | Bisphosphoglycerate mutase | P15327 | Up | 10.90 |
| 7 | Aldob | Fructose-bisphosphate aldolase B | Q91Y97 | Up | 9.40 |
| 8 | Slc4a1 | Band 3 anion transport protein | P04919 | Up | 8.99 |
| 9 | Mdh1 | Malate dehydrogenase, cytoplasmic | P14152 | Up | 8.31 |
| 10 | Ccl8 | C-C motif chemokine 8 | Q9Z121 | Up | 7.48 |
| 11 | Cat | Catalase | P24270 | Up | 6.66 |
| 12 | Pgk1 | Phosphoglycerate kinase 1 | P09411 | Up | 6.10 |
| 13 | Snca | Alpha-synuclein | O55042 | Up | 6.05 |
| 14 | Aldh1a1 | Retinal dehydrogenase 1 | P24549 | Up | 5.95 |
| 15 | Eno1 | Alpha-enolase | P17182 | Up | 5.90 |
| 16 | Gclm | Glutamate--cysteine ligase regulatory subunit | O09172 | Up | 5.31 |
| 17 | Calr | Calreticulin | P14211 | Up | 4.04 |
| 18 | Alad | Delta-aminolevulinic acid dehydratase | P10518 | Up | 4.00 |
| 19 | Blvrb | Flavin reductase (NADPH) | Q923D2 | Up | 3.61 |
| 20 | Prdx2 | Peroxiredoxin-2 | Q61171 | Up | 2.85 |
| 21 | Aldoa | Fructose-bisphosphate aldolase A | P05064 | Up | 1.72 |
| 22 | Saa4 | Serum amyloid A-4 protein | Saa4 | Down | 0.16 |
| 23 | Saa1 | Serum amyloid A-1 protein | Saa1 | Down | 0.07 |
| 24 | Cd44 | CD44 antigen | Cd44 | Down | 0.05 |

**Table S6.** Characterisation of the ageing-induced changes in plasma of C57BL/6J male mice based on expression ratio of markers (respective DEPs; upregulated: ratio ≥ 1.0, downregulated: ratio < 1.0) of ageing (28-week-old vs 8-week-old) dependent on strain and specific for sex in murine plasma in C57BL/6J mice. Presented table was prepared based on proteins that were significantly different between compared groups. Statistics: Student’s t-test. Supplementary Table S6 refers to Figure 8 of the main manuscript.

| **No.** | **Protein ID** | **Protein annotation** | **Accession** | **Regulation** | **Ratio** |
| --- | --- | --- | --- | --- | --- |
| 1 | Saa2 | Serum amyloid A-2 protein | P05367 | Up | 3949.07 |
| 2 | Saa1 | Serum amyloid A-1 protein | P05366 | Up | 291.00 |
| 3 | Hp | Haptoglobin | Q61646 | Up | 41.11 |
| 4 | Serpina3n | Serine protease inhibitor A3N | G3X8T9 | Up | 11.28 |
| 5 | Grn | Granulins | P28798 | Up | 11.01 |
| 6 | Chil3 | Chitinase-like protein 3 | O35744 | Up | 10.18 |
| 7 | Aldoa | Fructose-bisphosphate aldolase A | P05064 | Up | 9.52 |
| 8 | Pkm | Pyruvate kinase PKM | P52480-2 | Up | 6.99 |
| 9 | H6pd | GDH/6PGL endoplasmic bifunctional protein | Q8CFX1 | Up | 6.67 |
| 10 | Saa4 | Serum amyloid A-4 protein | P31532 | Up | 2.20 |
| 11 | Blvrb | Flavin reductase (NADPH) | Q923D2 | Down | 0.35 |
| 12 | Cat | Catalase | P24270 | Down | 0.26 |
| 13 | Prdx1 | Peroxiredoxin-1 | P35700 | Down | 0.21 |
| 14 | Man2b1 | Lysosomal alpha-mannosidase | O09159 | Down | 0.19 |
| 15 | Cd44 | CD44 antigen | P15379 | Down | 0.03 |

**Table S7.** Characterisation of the ageing-induced changes in plasma of E3L.CETP male mice based on expression ratio of markers (respective DEPs; upregulated: ratio ≥ 1.0. downregulated: ratio < 1.0) of ageing (28-week-old vs 8-week-old) dependent on strain and specific for sex in murine plasma in E3L.CETP mice. Presented table was prepared based on proteins that were significantly different between compared groups. Statistics: Student’s t-test. Supplementary Table S7 refers to Figure 8 of the main manuscript.

| **No.** | **Protein ID** | **Protein annotation** | **Accession** | **Regulation** | **Ratio** |
| --- | --- | --- | --- | --- | --- |
| 1 | Saa1 | Serum amyloid A-1 protein | P05366 | Up | 400.83 |
| 2 | Orm2 | Alpha-1-acid glycoprotein 2 | P07361 | Up | 176.01 |
| 3 | Lcn2 | Neutrophil gelatinase-associated lipocalin | A0A0A6YW77 | Up | 147.74 |
| 4 | Hp | Haptoglobin | Q61646 | Up | 82.94 |
| 5 | Serpina3n | Serine protease inhibitor A3N | G3X8T9 | Up | 4.28 |
| 6 | Chil3 | Chitinase-like protein 3 | O35744 | Up | 4.11 |
| 7 | Man2b1 | Lysosomal alpha-mannosidase | O09159 | Down | 0.37 |
| 8 | Calr | Calreticulin | P14211 | Down | 0.26 |
| 9 | Got2 | Aspartate aminotransferase, mitochondrial | P05202 | Down | 0.20 |
| 10 | Prdx1 | Peroxiredoxin-1 | P35700 | Down | 0.20 |
| 11 | Acaa2 | 3-ketoacyl-CoA thiolase, mitochondrial | Q8BWT1 | Down | 0.20 |
| 12 | Eno1 | Alpha-enolase | P17182 | Down | 0.20 |
| 13 | Blvrb | Flavin reductase (NADPH) | Q923D2 | Down | 0.19 |
| 14 | Cat | Catalase | P24270 | Down | 0.19 |
| 15 | Prdx2 | Peroxiredoxin-2 | Q61171 | Down | 0.18 |
| 16 | Gapdh | Glyceraldehyde-3-phosphate dehydrogenase | P16858 | Down | 0.17 |
| 17 | Gda | Guanine deaminase | Q9R111 | Down | 0.17 |
| 18 | Slc4a1 | Band 3 anion transport protein | P04919 | Down | 0.17 |
| 19 | Pnp | Purine nucleoside phosphorylase | P23492 | Down | 0.14 |
| 20 | Pgk1 | Phosphoglycerate kinase 1 | P09411 | Down | 0.13 |
| 21 | Alad | Delta-aminolevulinic acid dehydratase | P10518 | Down | 0.13 |
| 22 | Atic | Bifunctional purine biosynthesis protein PURH | Q9CWJ9 | Down | 0.12 |
| 23 | Gclm | Glutamate--cysteine ligase regulatory subunit | O09172 | Down | 0.10 |
| 24 | Prdx6 | Peroxiredoxin-6 | O08709 | Down | 0.10 |
| 25 | Alox12 | Arachidonate 12-lipoxygenase, 12S-type | P39655 | Down | 0.06 |
| 26 | Bpgm | Bisphosphoglycerate mutase | P15327 | Down | 0.04 |

**Table S8.** Ageing markers (respective DEPs) in murine aorta independent of strain but specific for sex of mice. Ratio of ageing (40-week-old vs 8-week-old) markers expression in murine aorta 43 upregulated (ratio ≥1.0) as well as 33 downregulated (ratio < 1.0) differentially expressed proteins (DEPs) specific for male in C57BL/6J mice. Presented table was prepared based on proteins that were significantly different between compared groups. Statistics: Student’s t-test. Supplementary Table S8 refers to Figure S8 of the supplementary material.

| **No.** | **Protein ID** | **Protein annotation** | **Accession** | **Regulation** | **Ratio** |
| --- | --- | --- | --- | --- | --- |
| 1 | Mpz | Myelin protein P0 | P27573 | Up | 5.19 |
| 2 | Dnah2 | Dynein heavy chain 2, axonemal | P0C6F1 | Up | 4.98 |
| 3 | Phyhd1 | Phytanoyl-CoA dioxygenase domain-containing protein 1 | Q9DB26 | Up | 2.98 |
| 4 | Ptgs1 | Prostaglandin G/H synthase 1 | P22437 | Up | 2.70 |
| 5 | Gsta3 | Glutathione S-transferase A3 | P30115 | Up | 2.57 |
| 6 | Prph | Peripherin | P15331 | Up | 2.44 |
| 7 | Dpep1 | Dipeptidase 1 | P31428 | Up | 2.25 |
| 8 | Dcxr | L-xylulose reductase | Q91X52 | Up | 2.16 |
| 9 | P2rx1 | P2X purinoceptor 1 | P51576 | Up | 2.12 |
| 10 | Sost | Sclerostin | Q99P68 | Up | 1.94 |
| 11 | Nt5e | 5-nucleotidase | Q61503 | Up | 1.83 |
| 12 | Gbe1 | 1,4-alpha-glucan-branching enzyme | Q9D6Y9 | Up | 1.83 |
| 13 | Gpc4 | Glypican-4 | P51655 | Up | 1.79 |
| 14 | Steap4 | Metalloreductase STEAP4 | Q923B6 | Up | 1.73 |
| 15 | Uchl1 | Ubiquitin carboxyl-terminal hydrolase isozyme L1 | Q9R0P9 | Up | 1.65 |
| 16 | Lgalsl | Galectin-related protein | Q8VED9 | Up | 1.64 |
| 17 | Hspb1 | Heat shock protein beta-1 | P14602 | Up | 1.64 |
| 18 | Itga5 | Integrin alpha-5 | P11688 | Up | 1.63 |
| 19 | Inpp4a | Type I inositol 3,4-bisphosphate 4-phosphatase | Q9EPW0 | Up | 1.61 |
| 20 | Ppap2a | Lipid phosphate phosphohydrolase 1 | Q61469 | Up | 1.52 |
| 21 | Cryab | Alpha-crystallin B chain | P23927 | Up | 1.47 |
| 22 | Hmgcs2 | Hydroxymethylglutaryl-CoA synthase, mitochondrial | P54869 | Up | 1.41 |
| 23 | Thy1 | Thy-1 membrane glycoprotein | P01831 | Up | 1.38 |
| 24 | Enpep | Glutamyl aminopeptidase | P16406 | Up | 1.32 |
| 25 | Nid1 | Nidogen-1 | P10493 | Up | 1.32 |
| 26 | Sgca | Alpha-sarcoglycan | P82350 | Up | 1.31 |
| 27 | Efhd2 | EF-hand domain-containing protein D2 | Q9D8Y0 | Up | 1.30 |
| 28 | Sun1 | SUN domain-containing protein 1 | Q9D666 | Up | 1.29 |
| 29 | Gstk1 | Glutathione S-transferase kappa 1 | Q9DCM2 | Up | 1.28 |
| 30 | Ugdh | UDP-glucose 6-dehydrogenase | O70475 | Up | 1.26 |
| 31 | Pbxip1 | Pre-B-cell leukemia transcription factor-interacting protein 1 | Q3TVI8 | Up | 1.24 |
| 32 | Psma1 | Proteasome subunit alpha type-1 | Q9R1P4 | Up | 1.24 |
| 33 | Aldh1a1 | Retinal dehydrogenase 1 | P24549 | Up | 1.23 |
| 34 | Gsto1 | Glutathione S-transferase omega-1 | O09131 | Up | 1.22 |
| 35 | Arhgdia | Rho GDP-dissociation inhibitor 1 | Q99PT1 | Up | 1.18 |
| 36 | Psmb7 | Proteasome subunit beta type-7 | P70195 | Up | 1.18 |
| 37 | Twf2 | Twinfilin-2 | Q9Z0P5 | Up | 1.17 |
| 38 | Loxl1 | Lysyl oxidase homolog 1 | P97873 | Up | 1.14 |
| 39 | Cpt1a | Carnitine O-palmitoyltransferase 1, liver isoform | P97742 | Up | 1.14 |
| 40 | Pon3 | Serum paraoxonase/lactonase 3 | Q62087 | Up | 1.14 |
| 41 | Mfap5 | Microfibrillar-associated protein 5 | Q9QZJ6 | Up | 1.13 |
| 42 | Oat | Ornithine aminotransferase, mitochondrial | P29758 | Up | 1.09 |
| 43 | Rab14 | Ras-related protein Rab-14 | Q91V41 | Up | 1.07 |
| 44 | Hyou1 | Hypoxia up-regulated protein 1 | Q9JKR6 | Down | 0.92 |
| 45 | Calm3 | Calmodulin-3 | P0DP28 | Down | 0.89 |
| 46 | Pa2g4 | Proliferation-associated protein 2G4 | P50580 | Down | 0.89 |
| 47 | Rab1A | Ras-related protein Rab-1A | P62821 | Down | 0.89 |
| 48 | Psmc2 | 26S protease regulatory subunit 7 | P46471 | Down | 0.89 |
| 49 | Lta4h | Leukotriene A-4 hydrolase | P24527 | Down | 0.88 |
| 50 | Ctsh | Pro-cathepsin H | P49935 | Down | 0.88 |
| 51 | Slmap | Sarcolemmal membrane-associated protein | Q3URD3 | Down | 0.87 |
| 52 | Ipo7 | Importin-7 | Q9EPL8 | Down | 0.86 |
| 53 | Rtcb | tRNA-splicing ligase RtcB homolog | Q99LF4 | Down | 0.86 |
| 54 | Txndc12 | Thioredoxin domain-containing protein 12 | Q9CQU0 | Down | 0.83 |
| 55 | Puf60 | Poly(U)-binding-splicing factor PUF60 | Q3UEB3 | Down | 0.83 |
| 56 | Sar1a | GTP-binding protein SAR1a | P36536 | Down | 0.83 |
| 57 | Ruvbl1 | RuvB-like 1 | P60122 | Down | 0.83 |
| 58 | Sec31a | Protein transport protein Sec31A | Q3UPL0 | Down | 0.79 |
| 59 | Dbi | Acyl-CoA-binding protein | P31786 | Down | 0.78 |
| 60 | Set | Protein SET | Q9EQU5 | Down | 0.77 |
| 61 | Cnn3 | Calponin-3 | Q9DAW9 | Down | 0.76 |
| 62 | Smarcc2 | SWI/SNF complex subunit SMARCC2 | Q6PDG5 | Down | 0.76 |
| 63 | Mlec | Malectin | Q6ZQI3 | Down | 0.76 |
| 64 | Lsm4 | U6 snRNA-associated Sm-like protein LSm4 | Q9QXA5 | Down | 0.75 |
| 65 | Arhgef7 | Rho guanine nucleotide exchange factor 7 | Q9ES28 | Down | 0.73 |
| 66 | Cast | Calpastatin | P51125 | Down | 0.69 |
| 67 | Colgalt1 | Procollagen galactosyltransferase 1 | Q8K297 | Down | 0.67 |
| 68 | Atp2b1;Atp2b2 | Plasma membrane calcium-transporting ATPase 1 | G5E829 | Down | 0.60 |
| 69 | Adck3 | Atypical kinase ADCK3, mitochondrial | Q60936 | Down | 0.57 |
| 70 | Rad21 | Double-strand-break repair protein rad21 homolog | Q61550 | Down | 0.52 |
| 71 | Naca | Nascent polypeptide-associated complex subunit alpha; | Q60817;P70670 | Down | 0.51 |
| 72 | Fgl2 | Fibroleukin | P12804 | Down | 0.50 |
| 73 | Rps21 | 40S ribosomal protein S21 | Q9CQR2 | Down | 0.49 |
| 74 | Hsbp1 | Heat shock factor-binding protein 1 | Q9CQZ1 | Down | 0.38 |
| 75 | Map2k3 | Dual specificity mitogen-activated protein kinase kinase 3 | O09110 | Down | 0.37 |
| 76 | Clec11a | C-type lectin domain family 11 member A | O88200 | Down | 0.36 |

**Table S9.** Ageing markers (respective DEPs) in murine aorta independent of strain but specific for sex of mice. Ratio of ageing (40-week-old vs 8-week-old) markers expression in murine aorta 43 upregulated (ratio ≥1.0) as well as 33 downregulated (ratio < 1.0) differentially expressed proteins (DEPs) specific for male in E3L.CETP mice. Presented table was prepared based on proteins that were significantly different between compared groups. Statistics: Student’s t-test. Supplementary Table S9 refers to Figure S8 of the supplementary material.

| **No.** | **Protein ID** | **Protein annotation** | **Accession** | **Regulation** | **Ratio** |
| --- | --- | --- | --- | --- | --- |
| 1 | Mpz | Myelin protein P0 | P27573 | Up | 4.28 |
| 2 | Prph | Peripherin | P15331 | Up | 3.42 |
| 3 | Gsta3 | Glutathione S-transferase A3 | P30115 | Up | 3.03 |
| 4 | Ptgs1 | Prostaglandin G/H synthase 1 | P22437 | Up | 2.39 |
| 5 | Dnah2 | Dynein heavy chain 2, axonemal | P0C6F1 | Up | 2.33 |
| 6 | Dcxr | L-xylulose reductase | Q91X52 | Up | 2.16 |
| 7 | Sost | Sclerostin | Q99P68 | Up | 1.89 |
| 8 | Dpep1 | Dipeptidase 1 | P31428 | Up | 1.86 |
| 9 | P2rx1 | P2X purinoceptor 1 | P51576 | Up | 1.85 |
| 10 | Lgalsl | Galectin-related protein | Q8VED9 | Up | 1.79 |
| 11 | Steap4 | Metalloreductase STEAP4 | Q923B6 | Up | 1.76 |
| 12 | Phyhd1 | Phytanoyl-CoA dioxygenase domain-containing protein 1 | Q9DB26 | Up | 1.68 |
| 13 | Hmgcs2 | Hydroxymethylglutaryl-CoA synthase, mitochondrial | P54869 | Up | 1.64 |
| 14 | Gbe1 | 1,4-alpha-glucan-branching enzyme | Q9D6Y9 | Up | 1.63 |
| 15 | Nt5e | 5-nucleotidase | Q61503 | Up | 1.59 |
| 16 | Itga5 | Integrin alpha-5 | P11688 | Up | 1.55 |
| 17 | Uchl1 | Ubiquitin carboxyl-terminal hydrolase isozyme L1 | Q9R0P9 | Up | 1.53 |
| 18 | Ppap2a | Lipid phosphate phosphohydrolase 1 | Q61469 | Up | 1.51 |
| 19 | Nid1 | Nidogen-1 | P10493 | Up | 1.45 |
| 20 | Hspb1 | Heat shock protein beta-1 | P14602 | Up | 1.42 |
| 21 | Cryab | Alpha-crystallin B chain | P23927 | Up | 1.41 |
| 22 | Thy1 | Thy-1 membrane glycoprotein | P01831 | Up | 1.40 |
| 23 | Mfap5 | Microfibrillar-associated protein 5 | Q9QZJ6 | Up | 1.32 |
| 24 | Sun1 | SUN domain-containing protein 1 | Q9D666 | Up | 1.32 |
| 25 | Gstk1 | Glutathione S-transferase kappa 1 | Q9DCM2 | Up | 1.30 |
| 26 | Loxl1 | Lysyl oxidase homolog 1 | P97873 | Up | 1.27 |
| 27 | Gpc4 | Glypican-4 | P51655 | Up | 1.25 |
| 28 | Gsto1 | Glutathione S-transferase omega-1 | O09131 | Up | 1.24 |
| 29 | Ugdh | UDP-glucose 6-dehydrogenase | O70475 | Up | 1.23 |
| 30 | Inpp4a | Type I inositol 3,4-bisphosphate 4-phosphatase | Q9EPW0 | Up | 1.23 |
| 31 | Efhd2 | EF-hand domain-containing protein D2 | Q9D8Y0 | Up | 1.20 |
| 32 | Sgca | Alpha-sarcoglycan | P82350 | Up | 1.20 |
| 33 | Aldh1a1 | Retinal dehydrogenase 1 | P24549 | Up | 1.19 |
| 34 | Enpep | Glutamyl aminopeptidase | P16406 | Up | 1.19 |
| 35 | Psma1 | Proteasome subunit alpha type-1 | Q9R1P4 | Up | 1.19 |
| 36 | Arhgdia | Rho GDP-dissociation inhibitor 1 | Q99PT1 | Up | 1.16 |
| 37 | Oat | Ornithine aminotransferase, mitochondrial | P29758 | Up | 1.15 |
| 38 | Cpt1a | Carnitine O-palmitoyltransferase 1, liver isoform | P97742 | Up | 1.15 |
| 39 | Psmb7 | Proteasome subunit beta type-7 | P70195 | Up | 1.14 |
| 40 | Pon3 | Serum paraoxonase/lactonase 3 | Q62087 | Up | 1.14 |
| 41 | Twf2 | Twinfilin-2 | Q9Z0P5 | Up | 1.14 |
| 42 | Rab14 | Ras-related protein Rab-14 | Q91V41 | Up | 1.13 |
| 43 | Pbxip1 | Pre-B-cell leukemia transcription factor-interacting protein 1 | Q3TVI8 | Up | 1.10 |
| 44 | Lta4h | Leukotriene A-4 hydrolase | P24527 | Down | 0.91 |
| 45 | Calm3 | Calmodulin-3 | P0DP28 | Down | 0.91 |
| 46 | Rtcb | tRNA-splicing ligase RtcB homolog | Q99LF4 | Down | 0.90 |
| 47 | Rab1A | Ras-related protein Rab-1A | P62821 | Down | 0.89 |
| 48 | Slmap | Sarcolemmal membrane-associated protein | Q3URD3 | Down | 0.88 |
| 49 | Psmc2 | 26S protease regulatory subunit 7 | P46471 | Down | 0.88 |
| 50 | Arhgef7 | Rho guanine nucleotide exchange factor 7 | Q9ES28 | Down | 0.86 |
| 51 | Hyou1 | Hypoxia up-regulated protein 1 | Q9JKR6 | Down | 0.85 |
| 52 | Pa2g4 | Proliferation-associated protein 2G4 | P50580 | Down | 0.83 |
| 53 | Mlec | Malectin | Q6ZQI3 | Down | 0.83 |
| 54 | Ruvbl1 | RuvB-like 1 | P60122 | Down | 0.83 |
| 55 | Ctsh | Pro-cathepsin H | P49935 | Down | 0.82 |
| 56 | Txndc12 | Thioredoxin domain-containing protein 12 | Q9CQU0 | Down | 0.80 |
| 57 | Cast | Calpastatin | P51125 | Down | 0.80 |
| 58 | Ipo7 | Importin-7 | Q9EPL8 | Down | 0.80 |
| 59 | Smarcc2 | SWI/SNF complex subunit SMARCC2 | Q6PDG5 | Down | 0.80 |
| 60 | Sar1a | GTP-binding protein SAR1a | P36536 | Down | 0.79 |
| 61 | Set | Protein SET | Q9EQU5 | Down | 0.79 |
| 62 | Sec31a | Protein transport protein Sec31A | Q3UPL0 | Down | 0.78 |
| 63 | Cnn3 | Calponin-3 | Q9DAW9 | Down | 0.78 |
| 64 | Dbi | Acyl-CoA-binding protein | P31786 | Down | 0.71 |
| 65 | Lsm4 | U6 snRNA-associated Sm-like protein LSm4 | Q9QXA5 | Down | 0.70 |
| 66 | Atp2b1;Atp2b2 | Plasma membrane calcium-transporting ATPase 1 | G5E829 | Down | 0.69 |
| 67 | Colgalt1 | Procollagen galactosyltransferase 1 | Q8K297 | Down | 0.68 |
| 68 | Rad21 | Double-strand-break repair protein rad21 homolog | Q61550 | Down | 0.60 |
| 69 | Puf60 | Poly(U)-binding-splicing factor PUF60 | Q3UEB3 | Down | 0.57 |

**Table S10.** Ageing markers (respective DEPs) in murine aorta independent of strain but specific for sex of mice. Ratio of ageing (40-week-old vs 8-week-old) markers expression in murine aorta 49 upregulated (ratio ≥1.0) as well as 78 downregulated (ratio < 1.0) differentially expressed proteins (DEPs) specific for female in C57BL/6J mice. Presented table was prepared based on proteins that were significantly different between compared groups. Statistics: Student’s t-test. Supplementary Table S10 refers to Figure S8 of the supplementary material.

| **No.** | **Protein ID** | **Protein annotation** | **Accession** | **Regulation** | **Ratio** |
| --- | --- | --- | --- | --- | --- |
| 1 | Mbl1 | Mannose-binding protein A | P39039 | Up | 6.08 |
| 2 | Retn | Resistin | Q99P87 | Up | 2.35 |
| 3 | C1qtnf7 | Complement C1q tumor necrosis factor-related protein 7 | Q8BVD7 | Up | 2.34 |
| 4 | Spp2 | Secreted phosphoprotein 24 | Q8K1I3 | Up | 2.27 |
| 5 | Stx12 | Syntaxin-12 | Q9ER00 | Up | 1.90 |
| 6 | Ctgf | Connective tissue growth factor | P29268 | Up | 1.74 |
| 7 | Fbln7 | Fibulin-7 | Q501P1 | Up | 1.72 |
| 8 | Fnbp1 | Formin-binding protein 1 | Q80TY0 | Up | 1.51 |
| 9 | Wfs1 | Wolframin | P56695 | Up | 1.48 |
| 10 | Serpind1 | Heparin cofactor 2 | P49182 | Up | 1.43 |
| 11 | Cryz | Quinone oxidoreductase | P47199 | Up | 1.38 |
| 12 | Grhpr | Glyoxylate reductase/hydroxypyruvate reductase | Q91Z53 | Up | 1.34 |
| 13 | Tmx2 | Thioredoxin-related transmembrane protein 2 | Q9D710 | Up | 1.27 |
| 14 | Ist1 | IST1 homolog | Q9CX00 | Up | 1.23 |
| 15 | Dctn4 | Dynactin subunit 4 | Q8CBY8 | Up | 1.21 |
| 16 | Hint1 | Histidine triad nucleotide-binding protein 1 | P70349 | Up | 1.18 |
| 17 | Macrod1 | O-acetyl-ADP-ribose deacetylase MACROD1 | Q922B1 | Up | 1.18 |
| 18 | Gnb1 | Guanine nucleotide-binding protein G(I)/G(S)/G(T) subunit beta-1 | P62874 | Up | 1.17 |
| 19 | Rhob | Rho-related GTP-binding protein RhoB | P62746 | Up | 1.17 |
| 20 | Cd81 | CD81 antigen | P35762 | Up | 1.16 |
| 21 | Gpx3 | Glutathione peroxidase 3 | P46412 | Up | 1.15 |
| 22 | Gnb2 | Guanine nucleotide-binding protein G(I)/G(S)/G(T) subunit beta-2 | P62880 | Up | 1.14 |
| 23 | Pgrmc1 | Membrane-associated progesterone receptor component 1 | O55022 | Up | 1.14 |
| 24 | Prdx6 | Peroxiredoxin-6 | O08709 | Up | 1.12 |
| 25 | Rsu1 | Ras suppressor protein 1 | Q01730 | Up | 1.12 |
| 26 | Myl12b | Myosin regulatory light chain 12B | Q3THE2 | Up | 1.12 |
| 27 | Sorbs3 | Vinexin | Q9R1Z8 | Up | 1.11 |
| 28 | Itgb3 | Integrin beta-3 | O54890 | Up | 1.11 |
| 29 | Prosc | Proline synthase co-transcribed bacterial homolog protein | Q9Z2Y8 | Up | 1.11 |
| 30 | Emd | Emerin | O08579 | Up | 1.10 |
| 31 | Clic4 | Chloride intracellular channel protein 4 | Q9QYB1 | Up | 1.10 |
| 32 | Tgfb1i1 | Transforming growth factor beta-1-induced transcript 1 protein | Q62219 | Up | 1.10 |
| 33 | Apoh | Beta-2-glycoprotein 1 | Q01339 | Up | 1.10 |
| 34 | Ywhaz | 14-3-3 protein zeta/delta | P63101 | Up | 1.09 |
| 35 | Gart | Trifunctional purine biosynthetic protein adenosine-3 | Q64737 | Up | 1.09 |
| 36 | Wars | Tryptophan--tRNA ligase, cytoplasmic | P32921 | Up | 1.09 |
| 37 | Napa | Alpha-soluble NSF attachment protein | Q9DB05 | Up | 1.08 |
| 38 | Apoa1bp | NAD(P)H-hydrate epimerase | Q8K4Z3 | Up | 1.08 |
| 39 | Nutf2 | Nuclear transport factor 2 | P61971 | Up | 1.07 |
| 40 | Arl8b | ADP-ribosylation factor-like protein 8B | Q9CQW2 | Up | 1.06 |
| 41 | Cnn1 | Calponin-1 | Q08091 | Up | 1.06 |
| 42 | Glrx | Glutaredoxin-1 | Q9QUH0 | Up | 1.06 |
| 43 | Atp5o | ATP synthase subunit O, mitochondrial | Q9DB20 | Up | 1.05 |
| 44 | Ube2v2 | Ubiquitin-conjugating enzyme E2 variant 2 | Q9D2M8 | Up | 1.05 |
| 45 | Pfn1 | Profilin-1 | P62962 | Up | 1.05 |
| 46 | Pafah1b2 | Platelet-activating factor acetylhydrolase IB subunit beta | Q61206 | Up | 1.04 |
| 47 | Comt | Catechol O-methyltransferase | O88587 | Up | 1.04 |
| 48 | Itpa | Inosine triphosphate pyrophosphatase | Q9D892 | Up | 1.04 |
| 49 | Dnm1l | Dynamin-1-like protein | Q8K1M6 | Up | 1.03 |
| 50 | Taldo1 | Transaldolase | Q93092 | Down | 0.97 |
| 51 | Eif4a1 | Eukaryotic initiation factor 4A-I | P60843 | Down | 0.96 |
| 52 | Flnb | Filamin-B | Q80X90 | Down | 0.96 |
| 53 | UPF0568 protein | UPF0568 protein C14orf166 homolog | A0A8D8B1K4 | Down | 0.96 |
| 54 | St13 | Hsc70-interacting protein | Q99L47 | Down | 0.96 |
| 55 | Ddb1 | DNA damage-binding protein 1 | Q3U1J4 | Down | 0.96 |
| 56 | Iars | Isoleucine--tRNA ligase, cytoplasmic | Q8BU30 | Down | 0.95 |
| 57 | Srsf5 | Serine/arginine-rich splicing factor 5 | O35326 | Down | 0.95 |
| 58 | Dync1i2 | Cytoplasmic dynein 1 intermediate chain 2 | O88487 | Down | 0.94 |
| 59 | Epb41l2 | Band 4.1-like protein 2 | O70318 | Down | 0.94 |
| 60 | Arpc1b | Actin-related protein 2/3 complex subunit 1B | Q9WV32 | Down | 0.93 |
| 61 | Dync1li1 | Cytoplasmic dynein 1 light intermediate chain 1 | Q8R1Q8 | Down | 0.93 |
| 62 | Por | Non-selective voltage-gated ion channel VDAC1 | Q60932 | Down | 0.93 |
| 63 | Lin7c | Protein lin-7 homolog C | O88952 | Down | 0.92 |
| 64 | Cst3 | Cystatin-C | P21460 | Down | 0.92 |
| 65 | Phb | Prohibitin | P67778 | Down | 0.92 |
| 66 | Rab7a | Ras-related protein Rab-7a | P51150 | Down | 0.91 |
| 67 | Eif3d | Eukaryotic translation initiation factor 3 subunit D | O70194 | Down | 0.91 |
| 68 | Kif5b | Kinesin-1 heavy chain | Q61768 | Down | 0.91 |
| 69 | Prpf19 | Pre-mRNA-processing factor 19 | Q99KP6 | Down | 0.90 |
| 70 | Golim4 | Golgi integral membrane protein 4 | Q8BXA1 | Down | 0.90 |
| 71 | Sf3b3 | Splicing factor 3B subunit 3 | Q921M3 | Down | 0.90 |
| 72 | Eef2 | Elongation factor 2 | P58252 | Down | 0.90 |
| 73 | Clint1 | Clathrin interactor 1 | Q99KN9 | Down | 0.90 |
| 74 | Rps3 | 40S ribosomal protein S3 | P62908 | Down | 0.89 |
| 75 | Celf2 | CUGBP Elav-like family member 2 | Q9Z0H4 | Down | 0.88 |
| 76 | Ctnna1 | Catenin alpha-1 | P26231 | Down | 0.88 |
| 77 | Rps16 | 40S ribosomal protein S16 | P14131 | Down | 0.88 |
| 78 | Ddx21 | Nucleolar RNA helicase 2 | Q9JIK5 | Down | 0.87 |
| 79 | Khdrbs1 | KH domain-containing, RNA-binding, signal transduction-associated protein 1 | Q60749 | Down | 0.87 |
| 80 | Rps9 | 40S ribosomal protein S9 | Q6ZWN5 | Down | 0.87 |
| 81 | Tm9sf2 | Transmembrane 9 superfamily member 2 | P58021 | Down | 0.86 |
| 82 | Vapa | Vesicle-associated membrane protein-associated protein A | Q9WV55 | Down | 0.86 |
| 83 | Rpl18a | 60S ribosomal protein L18a | P62717 | Down | 0.86 |
| 84 | Hist1h2ah | Histone H2A type 1-H | Q8CGP6 | Down | 0.85 |
| 85 | Map1lc3a | Microtubule-associated proteins 1A/1B light chain 3A | Q91VR7 | Down | 0.85 |
| 86 | Aoc3 | Membrane primary amine oxidase | O70423 | Down | 0.85 |
| 87 | Msra | Mitochondrial peptide methionine sulfoxide reductase | Q9D6Y7 | Down | 0.85 |
| 88 | Ptrf | Polymerase I and transcript release factor | O54724 | Down | 0.84 |
| 89 | Rap1gds1 | Rap1 GTPase-GDP dissociation stimulator 1 | E9Q912 | Down | 0.83 |
| 90 | G3bp1 | Ras GTPase-activating protein-binding protein 1 | P97855 | Down | 0.83 |
| 91 | Mpdu1 | Mannose-P-dolichol utilization defect 1 protein | Q9R0Q9 | Down | 0.82 |
| 92 | Eef1g | Elongation factor 1-gamma | Q9D8N0 | Down | 0.82 |
| 93 | Sec61a1 | Protein transport protein Sec61 subunit alpha isoform 1 | P61620 | Down | 0.82 |
| 94 | Tcerg1 | Transcription elongation regulator 1 | Q8CGF7 | Down | 0.82 |
| 95 | H1f0 | Histone H1.0 | P10922 | Down | 0.81 |
| 96 | Lmf2 | Lipase maturation factor 2 | Q8C3X8 | Down | 0.81 |
| 97 | Ctnnb1 | Catenin beta-1 | Q02248 | Down | 0.81 |
| 98 | Sf3b1 | Splicing factor 3B subunit 1 | Q99NB9 | Down | 0.79 |
| 99 | Rps13 | 40S ribosomal protein S13 | P62301 | Down | 0.78 |
| 100 | Rps15a | 40S ribosomal protein S15a | P62245 | Down | 0.78 |
| 101 | Mesdc2 | LDLR chaperone MESD | Q9ERE7 | Down | 0.78 |
| 102 | Chd4 | Chromodomain-helicase-DNA-binding protein 4 | Q6PDQ2 | Down | 0.78 |
| 103 | Cbx3 | Chromobox protein homolog 3 | P23198 | Down | 0.76 |
| 104 | Dcun1d1 | DCN1-like protein 1 | Q9QZ73 | Down | 0.76 |
| 105 | Sirt2 | NAD-dependent protein deacetylase sirtuin-2 | Q8VDQ8 | Down | 0.76 |
| 106 | Clasp2 | CLIP-associating protein 2 | Q8BRT1 | Down | 0.76 |
| 107 | Srsf1 | Serine/arginine-rich splicing factor 1 | Q6PDM2 | Down | 0.75 |
| 108 | Caprin1 | Caprin-1 | Q60865 | Down | 0.74 |
| 109 | Rpl26 | 60S ribosomal protein L26 | P61255 | Down | 0.74 |
| 110 | Smc3 | Structural maintenance of chromosomes protein 3 | Q9CW03 | Down | 0.73 |
| 111 | Rps24 | 40S ribosomal protein S24 | P62849 | Down | 0.72 |
| 112 | Sh3bgr | SH3 domain-binding glutamic acid-rich protein | Q9WUZ7 | Down | 0.70 |
| 113 | Rpl10 | 60S ribosomal protein L10 | Q6ZWV3 | Down | 0.70 |
| 114 | Ctbp2 | C-terminal-binding protein 2 | P56546 | Down | 0.66 |
| 115 | Limch1 | LIM and calponin homology domains-containing protein 1 | Q3UH68 | Down | 0.64 |
| 116 | G3bp2 | Ras GTPase-activating protein-binding | P97379 | Down | 0.63 |
| 117 | Sf3a3 | Splicing factor 3A subunit 3 | Q9D554 | Down | 0.63 |
| 118 | Nisch | Nischarin | Q80TM9 | Down | 0.62 |
| 119 | Srrt | Serrate RNA effector molecule homolog | Q99MR6 | Down | 0.61 |
| 120 | Hist1h1c | Histone H1.2 | P15864 | Down | 0.60 |
| 121 | Tmed2 | Transmembrane emp24 domain-containing protein 2 | Q9R0Q3 | Down | 0.59 |
| 122 | Pdxdc1 | Pyridoxal-dependent decarboxylase domain-containing protein 1 | Q99K01 | Down | 0.51 |
| 123 | Eif1 | Eukaryotic translation initiation factor 1 | P48024 | Down | 0.48 |
| 124 | Rbm39 | RNA-binding protein 39 | Q8VH51 | Down | 0.44 |
| 125 | Map6 | Microtubule-associated protein 6 | Q7TSJ2 | Down | 0.41 |
| 126 | Myh7b | Myosin-7B | A2AQP0 | Down | 0.36 |
| 127 | H2afx | Histone H2AX | P27661 | Down | 0.31 |

**Table S11.** Ageing markers (respective DEPs) in murine aorta independent of strain but specific for sex of mice. Ratio of ageing (40-week-old vs 8-week-old) markers expression in murine aorta 49 upregulated (ratio ≥1.0) as well as 78 downregulated (ratio < 1.0) differentially expressed proteins (DEPs) specific for female in E3L.CETP mice. Presented table was prepared based on proteins that were significantly different between compared groups. Statistics: Student’s t-test. Supplementary Table S11 refers to Figure S8 of the supplementary material.

| **No.** | **Protein ID** | **Protein annotation** | **Accession** | **Regulation** | **Ratio** |
| --- | --- | --- | --- | --- | --- |
| 1 | Spp2 | Secreted phosphoprotein 24 | Q8K1I3 | Up | 6.35 |
| 2 | Mbl1 | Mannose-binding protein A | P39039 | Up | 4.24 |
| 3 | Ctgf | Connective tissue growth factor | P29268 | Up | 2.59 |
| 4 | Retn | Resistin | Q99P87 | Up | 2.53 |
| 5 | Fnbp1 | Formin-binding protein 1 | Q80TY0 | Up | 2.36 |
| 6 | C1qtnf7 | Complement C1q tumor necrosis factor-related protein 7 | Q8BVD7 | Up | 1.89 |
| 7 | Fbln7 | Fibulin-7 | Q501P1 | Up | 1.83 |
| 8 | Stx12 | Syntaxin-12 | Q9ER00 | Up | 1.67 |
| 9 | Dctn4 | Dynactin subunit 4 | Q8CBY8 | Up | 1.65 |
| 10 | Rhob | Rho-related GTP-binding protein RhoB | P62746 | Up | 1.57 |
| 11 | Cd81 | CD81 antigen | P35762 | Up | 1.56 |
| 12 | Serpind1 | Heparin cofactor 2 | P49182 | Up | 1.54 |
| 13 | Tmx2 | Thioredoxin-related transmembrane protein 2 | Q9D710 | Up | 1.43 |
| 14 | Ist1 | IST1 homolog | Q9CX00 | Up | 1.40 |
| 15 | Wfs1 | Wolframin | P56695 | Up | 1.39 |
| 16 | Apoh | Beta-2-glycoprotein 1 | Q01339 | Up | 1.36 |
| 17 | Hint1 | Histidine triad nucleotide-binding protein 1 | P70349 | Up | 1.35 |
| 18 | Cryz | Quinone oxidoreductase | P47199 | Up | 1.34 |
| 19 | Cnn1 | Calponin-1 | Q08091 | Up | 1.33 |
| 20 | Rsu1 | Ras suppressor protein 1 | Q01730 | Up | 1.31 |
| 21 | Prosc | Proline synthase co-transcribed bacterial homolog protein | Q9Z2Y8 | Up | 1.30 |
| 22 | Itpa | Inosine triphosphate pyrophosphatase | Q9D892 | Up | 1.30 |
| 23 | Grhpr | Glyoxylate reductase/hydroxypyruvate reductase | Q91Z53 | Up | 1.29 |
| 24 | Nutf2 | Nuclear transport factor 2 | P61971 | Up | 1.29 |
| 25 | Glrx | Glutaredoxin-1 | Q9QUH0 | Up | 1.27 |
| 26 | Pgrmc1 | Membrane-associated progesterone receptor component 1 | O55022 | Up | 1.26 |
| 27 | Sorbs3 | Vinexin | Q9R1Z8 | Up | 1.26 |
| 28 | Tgfb1i1 | Transforming growth factor beta-1-induced transcript 1 protein | Q62219 | Up | 1.25 |
| 29 | Myl12b | Myosin regulatory light chain 12B | Q3THE2 | Up | 1.24 |
| 30 | Pafah1b2 | Platelet-activating factor acetylhydrolase IB subunit beta | Q61206 | Up | 1.23 |
| 31 | Arl8b | ADP-ribosylation factor-like protein 8B | Q9CQW2 | Up | 1.22 |
| 32 | Wars | Tryptophan--tRNA ligase, cytoplasmic | P32921 | Up | 1.22 |
| 33 | Gpx3 | Glutathione peroxidase 3 | P46412 | Up | 1.22 |
| 34 | Gnb1 | Guanine nucleotide-binding protein G(I)/G(S)/G(T) subunit beta-1 | P62874 | Up | 1.21 |
| 35 | Clic4 | Chloride intracellular channel protein 4 | Q9QYB1 | Up | 1.20 |
| 36 | Pfn1 | Profilin-1 | P62962 | Up | 1.20 |
| 37 | Atp5o | ATP synthase subunit O, mitochondrial | Q9DB20 | Up | 1.19 |
| 38 | Prdx6 | Peroxiredoxin-6 | O08709 | Up | 1.19 |
| 39 | Gnb2 | Guanine nucleotide-binding protein G(I)/G(S)/G(T) subunit beta-2 | P62880 | Up | 1.17 |
| 40 | Emd | Emerin | O08579 | Up | 1.17 |
| 41 | Apoa1bp | NAD(P)H-hydrate epimerase | Q8K4Z3 | Up | 1.16 |
| 42 | Ube2v2 | Ubiquitin-conjugating enzyme E2 variant 2 | Q9D2M8 | Up | 1.15 |
| 43 | Itgb3 | Integrin beta-3 | O54890 | Up | 1.15 |
| 44 | Macrod1 | O-acetyl-ADP-ribose deacetylase MACROD1 | Q922B1 | Up | 1.15 |
| 45 | Gart | Trifunctional purine biosynthetic protein adenosine-3 | Q64737 | Up | 1.12 |
| 46 | Dnm1l | Dynamin-1-like protein | Q8K1M6 | Up | 1.11 |
| 47 | Napa | Alpha-soluble NSF attachment protein | Q9DB05 | Up | 1.10 |
| 48 | Comt | Catechol O-methyltransferase | O88587 | Up | 1.10 |
| 49 | Ywhaz | 14-3-3 protein zeta/delta | P63101 | Up | 1.09 |
| 50 | Dync1i2 | Cytoplasmic dynein 1 intermediate chain 2 | O88487 | Down | 0.93 |
| 51 | Flnb | Filamin-B | Q80X90 | Down | 0.92 |
| 52 | Eef2 | Elongation factor 2 | P58252 | Down | 0.92 |
| 53 | Eif4a1 | Eukaryotic initiation factor 4A-I | P60843 | Down | 0.91 |
| 54 | Ddb1 | DNA damage-binding protein 1 | Q3U1J4 | Down | 0.91 |
| 55 | Arpc1b | Actin-related protein 2/3 complex subunit 1B | Q9WV32 | Down | 0.90 |
| 56 | Smc3 | Structural maintenance of chromosomes protein 3 | Q9CW03 | Down | 0.90 |
| 57 | St13 | Hsc70-interacting protein | Q99L47 | Down | 0.90 |
| 58 | Kif5b | Kinesin-1 heavy chain | Q61768 | Down | 0.90 |
| 59 | Ptrf | Polymerase I and transcript release factor | O54724 | Down | 0.90 |
| 60 | Epb41l2 | Band 4.1-like protein 2 | O70318 | Down | 0.90 |
| 61 | Eif3d | Eukaryotic translation initiation factor 3 subunit D | O70194 | Down | 0.89 |
| 62 | Ctnnb1 | Catenin beta-1 | Q02248 | Down | 0.88 |
| 63 | Rab7a | Ras-related protein Rab-7a | P51150 | Down | 0.88 |
| 64 | Cst3 | Cystatin-C | P21460 | Down | 0.88 |
| 65 | Rps15a | 40S ribosomal protein S15a | P62245 | Down | 0.87 |
| 66 | Caprin1 | Caprin-1 | Q60865 | Down | 0.87 |
| 67 | Rps9 | 40S ribosomal protein S9 | Q6ZWN5 | Down | 0.87 |
| 68 | Vapa | Vesicle-associated membrane protein-associated protein A | Q9WV55 | Down | 0.87 |
| 69 | Hist1h1c | Histone H1.2 | P15864 | Down | 0.87 |
| 70 | Rap1gds1 | Rap1 GTPase-GDP dissociation stimulator 1 | E9Q912 | Down | 0.86 |
| 71 | Nisch | Nischarin | Q80TM9 | Down | 0.86 |
| 72 | UPF0568 protein | UPF0568 protein C14orf166 homolog | A0A8D8B1K4 | Down | 0.86 |
| 73 | Iars | Isoleucine--tRNA ligase, cytoplasmic | Q8BU30 | Down | 0.85 |
| 74 | Aoc3 | Membrane primary amine oxidase | O70423 | Down | 0.85 |
| 75 | Lin7c | Protein lin-7 homolog C | O88952 | Down | 0.85 |
| 76 | Mesdc2 | LDLR chaperone MESD | Q9ERE7 | Down | 0.85 |
| 77 | G3bp1 | Ras GTPase-activating protein-binding protein 1 | P97855 | Down | 0.85 |
| 78 | Tm9sf2 | Transmembrane 9 superfamily member 2 | P58021 | Down | 0.84 |
| 79 | Taldo1 | Transaldolase | Q93092 | Down | 0.84 |
| 80 | Sf3b1 | Splicing factor 3B subunit 1 | Q99NB9 | Down | 0.83 |
| 81 | Rpl18a | 60S ribosomal protein L18a | P62717 | Down | 0.83 |
| 82 | Srsf5 | Serine/arginine-rich splicing factor 5 | O35326 | Down | 0.83 |
| 83 | Clint1 | Clathrin interactor 1 | Q99KN9 | Down | 0.82 |
| 84 | Sec61a1 | Protein transport protein Sec61 subunit alpha isoform 1 | P61620 | Down | 0.82 |
| 85 | Rpl10;Rpl10l | 60S ribosomal protein L10 | Q6ZWV3 | Down | 0.82 |
| 86 | Srsf1 | Serine/arginine-rich splicing factor 1 | Q6PDM2 | Down | 0.81 |
| 87 | Ctnna1 | Catenin alpha-1 | P26231 | Down | 0.81 |
| 88 | Sf3b3 | Splicing factor 3B subunit 3 | Q921M3 | Down | 0.81 |
| 89 | Rps16 | 40S ribosomal protein S16 | P14131 | Down | 0.80 |
| 90 | Rps13 | 40S ribosomal protein S13 | P62301 | Down | 0.80 |
| 91 | Golim4 | Golgi integral membrane protein 4 | Q8BXA1 | Down | 0.80 |
| 92 | Prpf19 | Pre-mRNA-processing factor 19 | Q99KP6 | Down | 0.80 |
| 93 | H1f0 | Histone H1.0 | P10922 | Down | 0.80 |
| 94 | Clasp2 | CLIP-associating protein 2 | Q8BRT1 | Down | 0.80 |
| 95 | Rps3 | 40S ribosomal protein S3 | P62908 | Down | 0.80 |
| 96 | Eef1g | Elongation factor 1-gamma | Q9D8N0 | Down | 0.79 |
| 97 | Map1lc3a | Microtubule-associated proteins 1A/1B light chain 3A | Q91VR7 | Down | 0.79 |
| 98 | Rps24 | 40S ribosomal protein S24 | P62849 | Down | 0.79 |
| 99 | Hist1h2ah | Histone H2A type 1-H | Q8CGP6 | Down | 0.79 |
| 100 | Msra | Mitochondrial peptide methionine sulfoxide reductase | Q9D6Y7 | Down | 0.78 |
| 101 | Phb | Prohibitin | P67778 | Down | 0.78 |
| 102 | Dcun1d1 | DCN1-like protein 1 | Q9QZ73 | Down | 0.76 |
| 103 | Dync1li1 | Cytoplasmic dynein 1 light intermediate chain 1 | Q8R1Q8 | Down | 0.76 |
| 104 | Tcerg1 | Transcription elongation regulator 1 | Q8CGF7 | Down | 0.75 |
| 105 | Ddx21 | Nucleolar RNA helicase 2 | Q9JIK5 | Down | 0.75 |
| 106 | Sh3bgr | SH3 domain-binding glutamic acid-rich protein | Q9WUZ7 | Down | 0.74 |
| 107 | Celf2 | CUGBP Elav-like family member 2 | Q9Z0H4 | Down | 0.72 |
| 108 | Por | Non-selective voltage-gated ion channel VDAC1 | Q60932 | Down | 0.72 |
| 109 | Khdrbs1 | KH domain-containing, RNA-binding, signal transduction-associated protein 1 | Q60749 | Down | 0.71 |
| 110 | Limch1 | LIM and calponin homology domains-containing protein 1 | Q3UH68 | Down | 0.71 |
| 111 | Rpl26 | 60S ribosomal protein L26 | P61255 | Down | 0.71 |
| 112 | Tmed2 | Transmembrane emp24 domain-containing protein 2 | Q9R0Q3 | Down | 0.70 |
| 113 | Mpdu1 | Mannose-P-dolichol utilization defect 1 protein | Q9R0Q9 | Down | 0.65 |
| 114 | Chd4 | Chromodomain-helicase-DNA-binding protein 4 | Q6PDQ2 | Down | 0.64 |
| 115 | Sf3a3 | Splicing factor 3A subunit 3 | Q9D554 | Down | 0.61 |
| 116 | Sirt2 | NAD-dependent protein deacetylase sirtuin-2 | Q8VDQ8 | Down | 0.61 |
| 117 | Lmf2 | Lipase maturation factor 2 | Q8C3X8 | Down | 0.59 |
| 118 | Ctbp2 | C-terminal-binding protein 2 | P56546 | Down | 0.57 |
| 119 | Rbm39 | RNA-binding protein 39 | Q8VH51 | Down | 0.57 |
| 120 | Srrt | Serrate RNA effector molecule homolog | Q99MR6 | Down | 0.55 |
| 121 | Pdxdc1 | Pyridoxal-dependent decarboxylase domain-containing protein 1 | Q99K01 | Down | 0.47 |
| 122 | Cbx3 | Chromobox protein homolog 3 | P23198 | Down | 0.46 |
| 123 | Myh7b | Myosin-7B | A2AQP0 | Down | 0.46 |
| 124 | H2afx | Histone H2AX | P27661 | Down | 0.39 |
| 125 | G3bp2 | Ras GTPase-activating protein-binding | P97379 | Down | 0.38 |
| 126 | Map6 | Microtubule-associated protein 6 | Q7TSJ2 | Down | 0.35 |
| 127 | Eif1 | Eukaryotic translation initiation factor 1 | P48024 | Down | 0.19 |

**Table S12.** Hyperlipidaemia markers (respective DEPs) in murine aorta independent of age but specific for sex of mice. Ratio of hyperlipidaemia (E3L.CETP vs C57BL/6J) markers expression in murine aorta of 3 upregulated (ratio ≥1.0) as well as 1 downregulated (ratio < 1.0) differentially expressed proteins (DEPs) specific for male in 8-week-old mice. Presented table was prepared based on proteins that were significantly different between compared groups. Statistics: Student’s t-test. Supplementary Table S12 refers to Figure S11 of the supplementary material.

| **No.** | **Protein ID** | **Protein annotation** | **Accession** | **Regulation** | **Ratio** |
| --- | --- | --- | --- | --- | --- |
| 1 | Tgfbi | Transforming growth factor-beta-induced protein ig-h3 | P82198 | Up | 1.15 |
| 2 | Ndrg1 | Protein NDRG1 | Q62433 | Up | 1.14 |
| 3 | Aspn | Asporin | Q99MQ4 | Up | 1.12 |
| 4 | Cnn3 | Calponin-3 | Q9DAW9 | Down | 0.79 |

**Table S13.** Hyperlipidaemia markers (respective DEPs) in murine aorta independent of age but specific for sex of mice. Ratio of hyperlipidaemia (E3L.CETP vs C57BL/6J) markers expression in murine aorta of 3 upregulated (ratio ≥1.0) as well as 1 downregulated (ratio < 1.0) differentially expressed proteins (DEPs) specific for male in 40-week-old mice. Presented table was prepared based on proteins that were significantly different between compared groups. Statistics: Student’s t-test. Supplementary Table S13 refers to Figure S11 of the supplementary material.

| **No.** | **Protein ID** | **Protein annotation** | **Accession** | **Regulation** | **Ratio** |
| --- | --- | --- | --- | --- | --- |
| 1 | Tgfbi | Transforming growth factor-beta-induced protein ig-h3 | P82198 | Up | 1.23 |
| 2 | Aspn | Asporin | Q99MQ4 | Up | 1.21 |
| 3 | Ndrg1 | Protein NDRG1 | Q62433 | Up | 1.13 |
| 4 | Cnn3 | Calponin-3 | Q9DAW9 | Down | 0.81 |

**Table S14.** Hyperlipidaemia markers (respective DEPs) in murine aorta independent of age but specific for sex of mice. Ratio of hyperlipidaemia (E3L.CETP vs C57BL/6J) markers expression in murine aorta of 1 upregulated (ratio ≥1.0) as well as 13 downregulated (ratio < 1.0) differentially expressed proteins (DEPs) specific for female in 8-week-old mice. Presented table was prepared based on proteins that were significantly different between compared groups. Statistics: Student’s t-test. Supplementary Table S14 refers to Figure S11 of the supplementary material.

| **No.** | **Protein ID** | **Protein annotation** | **Accession** | **Regulation** | **Ratio** |
| --- | --- | --- | --- | --- | --- |
| 1 | Itih4 | Inter alpha-trypsin inhibitor, heavy chain 4 | A6X935 | Up | 1.30 |
| 2 | Vars | Valine--tRNA ligase | Q9Z1Q9 | Down | 0.92 |
| 3 | Lrp1 | Prolow-density lipoprotein receptor-related protein 1 | Q91ZX7 | Down | 0.91 |
| 4 | Cpt1a | Carnitine O-palmitoyltransferase 1, liver isoform | P97742 | Down | 0.91 |
| 5 | Clint1 | Clathrin interactor 1 | Q99KN9 | Down | 0.90 |
| 6 | Snrnp70 | U1 small nuclear ribonucleoprotein 70 kDa | Q62376 | Down | 0.90 |
| 7 | Khsrp | Far upstream element-binding protein 2 | Q3U0V1 | Down | 0.89 |
| 8 | Sec23a | Protein transport protein Sec23A | Q01405 | Down | 0.89 |
| 9 | Eea1 | Early endosome antigen 1 | Q8BL66 | Down | 0.89 |
| 10 | Fblim1 | Filamin-binding LIM protein 1 | Q71FD7 | Down | 0.87 |
| 11 | Jph2 | Junctophilin-2 | Q9ET78 | Down | 0.87 |
| 12 | Cst3 | Cystatin-C | P21460 | Down | 0.86 |
| 13 | Trim28 | Transcription intermediary factor 1-beta | Q62318 | Down | 0.62 |
| 14 | Ddb1 | DNA damage-binding protein 1 | Q3U1J4 | Down | 0.60 |

**Table S15.** Hyperlipidaemia markers (respective DEPs) in murine aorta independent of age but specific for sex of mice. Ratio of hyperlipidaemia (E3L.CETP vs C57BL/6J) markers expression in murine aorta of 1 upregulated (ratio ≥1.0) as well as 13 downregulated (ratio < 1.0) differentially expressed proteins (DEPs) specific for female in 40-week-old mice. Presented table was prepared based on proteins that were significantly different between compared groups. Statistics: Student’s t-test. Supplementary Table S15 refers to Figure S11 of the supplementary material.

| **No.** | **Protein ID** | **Protein annotation** | **Accession** | **Regulation** | **Ratio** |
| --- | --- | --- | --- | --- | --- |
| 1 | Itih4 | Inter alpha-trypsin inhibitor, heavy chain 4 | A6X935 | Up | 1.16 |
| 2 | Lrp1 | Prolow-density lipoprotein receptor-related protein 1 | Q91ZX7 | Down | 0.92 |
| 3 | Eea1 | Early endosome antigen 1 | Q8BL66 | Down | 0.92 |
| 4 | Vars | Valine--tRNA ligase | Q9Z1Q9 | Down | 0.92 |
| 5 | Khsrp | Far upstream element-binding protein 2 | Q3U0V1 | Down | 0.89 |
| 6 | Fblim1 | Filamin-binding LIM protein 1 | Q71FD7 | Down | 0.89 |
| 7 | Cpt1a | Carnitine O-palmitoyltransferase 1, liver isoform | P97742 | Down | 0.87 |
| 8 | Ddb1 | DNA damage-binding protein 1 | Q3U1J4 | Down | 0.87 |
| 9 | Trim28 | Transcription intermediary factor 1-beta | Q62318 | Down | 0.86 |
| 10 | Snrnp70 | U1 small nuclear ribonucleoprotein 70 kDa | Q62376 | Down | 0.85 |
| 11 | Sec23a | Protein transport protein Sec23A | Q01405 | Down | 0.83 |
| 12 | Clint1 | Clathrin interactor 1 | Q99KN9 | Down | 0.82 |
| 13 | Cst3 | Cystatin-C | P21460 | Down | 0.82 |
| 14 | Jph2 | Junctophilin-2 | Q9ET78 | Down | 0.78 |

**Table S16.** Ageing markers (respective DEPs) in murine plasma independent of strain but specific for sex of mice. Ratio of ageing (28-week-old vs 8-week-old) markers expression in murine aorta 19 upregulated (ratio ≥1.0) as well as 21 downregulated (ratio < 1.0) differentially expressed proteins (DEPs) specific for male in C57BL/6J mice. Presented table was prepared based on proteins that were significantly different between compared groups. Statistics: Student’s t-test. Supplementary Table S16 refers to Figure S14 of the supplementary material.

| **No.** | **Protein ID** | **Protein annotation** | **Accession** | **Regulation** | **Ratio** |
| --- | --- | --- | --- | --- | --- |
| 1 | Saa1 | Serum amyloid A-1 protein | P05366 | Up | 291.00 |
| 2 | Orm2 | Alpha-1-acid glycoprotein 2 | P07361 | Up | 143.29 |
| 3 | Fgl1 | Fibrinogen-like protein 1 | Q71KU9 | Up | 139.62 |
| 4 | Prg4 | Proteoglycan 4 | E9QQ18 | Up | 43.20 |
| 5 | Hp | Haptoglobin | Q61646 | Up | 41.11 |
| 6 | Ngp | Neutrophilic granule protein | O08692 | Up | 17.83 |
| 7 | Serpina3n | Serine protease inhibitor A3N | G3X8T9 | Up | 11.28 |
| 8 | Chil3 | Chitinase-like protein 3 | O35744 | Up | 10.18 |
| 9 | Ltf | Lactotransferrin | P08071 | Up | 8.25 |
| 10 | Lrg1 | Lrg1 | Q91XL1 | Up | 5.94 |
| 11 | Itih3 | Inter-alpha-trypsin inhibitor heavy chain H3 | Q61704 | Up | 5.22 |
| 12 | Orm1 | Alpha-1-acid glycoprotein 1 | Q60590 | Up | 4.98 |
| 13 | Itih4 | Itih4 | E9PVD2 | Up | 4.25 |
| 14 | Hpx | Hemopexin | Q91X72 | Up | 2.69 |
| 15 | Saa1 | Serum amyloid A-1 protein | P05366 | Up | 291.00 |
| 16 | Orm2 | Alpha-1-acid glycoprotein 2 | P07361 | Up | 143.29 |
| 17 | Fgl1 | Fibrinogen-like protein 1 | Q71KU9 | Up | 139.62 |
| 18 | Prg4 | Proteoglycan 4 | E9QQ18 | Up | 43.20 |
| 19 | Hp | Haptoglobin | Q61646 | Up | 41.11 |
| 20 | Plxdc2 | Plexin domain-containing protein 2 | Q9DC11 | Down | 0.65 |
| 21 | Tfrc | Transferrin receptor protein 1 | Q62351 | Down | 0.53 |
| 22 | Adipoq | Adiponectin | Q60994 | Down | 0.52 |
| 23 | Ces1b | Carboxylic ester hydrolase | D3Z5G7 | Down | 0.51 |
| 24 | Cfp | Properdin | P11680 | Down | 0.49 |
| 25 | HBZ2 | Hemoglobin subunit zeta | Q78PA4 | Down | 0.38 |
| 26 | Hbbt1 | Hbbt1 | A8DUK4 | Down | 0.36 |
| 27 | Serpina6 | Corticosteroid-binding globulin | Q06770 | Down | 0.36 |
| 28 | Blvrb | Flavin reductase (NADPH) | Q923D2 | Down | 0.35 |
| 29 | Mgam | Mgam | B5THE2 | Down | 0.33 |
| 30 | Cat | Catalase | P24270 | Down | 0.26 |
| 31 | Gpx1 | Glutathione peroxidase 1 | P11352 | Down | 0.24 |
| 32 | Pf4 | Platelet factor 4 | Q9Z126 | Down | 0.24 |
| 33 | Prdx2 | Peroxiredoxin-2 | Q61171 | Down | 0.21 |
| 34 | Man2b1 | Lysosomal alpha-mannosidase | O09159 | Down | 0.19 |
| 35 | Ca1 | Carbonic anhydrase 1 | P13634 | Down | 0.18 |
| 36 | Fermt3 | Fermitin family homolog 3 | Q8K1B8 | Down | 0.15 |
| 37 | Ncam1 | Neural cell adhesion molecule 1 | P13595 | Down | 0.12 |
| 38 | Cap1 | Adenylyl cyclase-associated protein 1 | P40124 | Down | 0.11 |
| 39 | Ilk | Integrin-linked protein kinase | O55222 | Down | 0.10 |
| 40 | Rab6b | Ras-related protein Rab-6B | P61294 | Down | 0.05 |

**Table S17.** Ageing markers (respective DEPs) in murine plasma independent of strain but specific for sex of mice. Ratio of ageing (28-week-old vs 8-week-old) markers expression in murine aorta 19 upregulated (ratio ≥1.0) as well as 21 downregulated (ratio < 1.0) differentially expressed proteins (DEPs) specific for male in E3L.CETP mice. Presented table was prepared based on proteins that were significantly different between compared groups. Statistics: Student’s t-test. Supplementary Table S17 refers to Figure S14 of the supplementary material.

| **No.** | **Protein ID** | **Protein annotation** | **Accession** | **Regulation** | **Ratio** |
| --- | --- | --- | --- | --- | --- |
| 1 | Saa1 | Serum amyloid A-1 protein | P05366 | Up | 400.83 |
| 2 | Orm2 | Alpha-1-acid glycoprotein 2 | P07361 | Up | 176.01 |
| 3 | Hp | Haptoglobin | Q61646 | Up | 82.94 |
| 4 | Ngp | Neutrophilic granule protein | O08692 | Up | 17.27 |
| 5 | Prg4 | Proteoglycan 4 | E9QQ18 | Up | 17.21 |
| 6 | Fgl1 | Fibrinogen-like protein 1 | Q71KU9 | Up | 9.03 |
| 7 | Ltf | Lactotransferrin | P08071 | Up | 8.94 |
| 8 | Serpina3n | Serine protease inhibitor A3N | G3X8T9 | Up | 4.28 |
| 9 | Chil3 | Chitinase-like protein 3 | O35744 | Up | 4.11 |
| 10 | Lrg1 | Lrg1 | Q91XL1 | Up | 4.06 |
| 11 | Orm1 | Alpha-1-acid glycoprotein 1 | Q60590 | Up | 3.30 |
| 12 | Itih4 | Itih4 | E9PVD2 | Up | 2.66 |
| 13 | Itih3 | Inter-alpha-trypsin inhibitor heavy chain H3 | Q61704 | Up | 2.65 |
| 14 | Hpx | Hemopexin | Q91X72 | Up | 1.99 |
| 15 | Clu | Clusterin | Q06890 | Up | 1.98 |
| 16 | Psma4 | Proteasome subunit alpha type-4 | Q9R1P0 | Up | 1.79 |
| 17 | C4bpa | C4b-binding protein | P08607 | Up | 1.69 |
| 18 | Cpb2 | Carboxypeptidase B2 | Q9JHH6 | Up | 1.60 |
| 19 | Il18bp | Interleukin-18-binding protein | Q9Z0M9 | Up | 1.56 |
| 20 | Tfrc | Transferrin receptor protein 1 | Q62351 | Down | 0.67 |
| 21 | Plxdc2 | Plexin domain-containing protein 2 | Q9DC11 | Down | 0.65 |
| 22 | Adipoq | Adiponectin | Q60994 | Down | 0.57 |
| 23 | Ces1b | Carboxylic ester hydrolase | D3Z5G7 | Down | 0.56 |
| 24 | Cfp | Properdin | P11680 | Down | 0.50 |
| 25 | Mgam | Mgam | B5THE2 | Down | 0.48 |
| 26 | Serpina6 | Corticosteroid-binding globulin | Q06770 | Down | 0.48 |
| 27 | Ncam1 | Neural cell adhesion molecule 1 | P13595 | Down | 0.46 |
| 28 | Man2b1 | Lysosomal alpha-mannosidase | O09159 | Down | 0.37 |
| 29 | Pf4 | Platelet factor 4 | Q9Z126 | Down | 0.33 |
| 30 | Hbbt1 | Hbbt1 | A8DUK4 | Down | 0.32 |
| 31 | HBZ2 | Hemoglobin subunit zeta | Q78PA4 | Down | 0.30 |
| 32 | Prdx2 | Peroxiredoxin-2 | Q61171 | Down | 0.20 |
| 33 | Blvrb | Flavin reductase (NADPH) | Q923D2 | Down | 0.19 |
| 34 | Cat | Catalase | P24270 | Down | 0.19 |
| 35 | Fermt3 | Fermitin family homolog 3 | Q8K1B8 | Down | 0.18 |
| 36 | Ca1 | Carbonic anhydrase 1 | P13634 | Down | 0.17 |
| 37 | Gpx1 | Glutathione peroxidase 1 | P11352 | Down | 0.12 |
| 38 | Ilk | Integrin-linked protein kinase | O55222 | Down | 0.10 |
| 39 | Cap1 | Adenylyl cyclase-associated protein 1 | P40124 | Down | 0.08 |
| 40 | Rab6b | Ras-related protein Rab-6B | P61294 | Down | 0.07 |

**Table S18.** Ageing markers (respective DEPs) in murine plasma independent of strain but specific for sex of mice. Ratio of ageing (28-week-old vs 8-week-old) markers expression in murine aorta 2 upregulated (ratio ≥1.0) as well as 3 downregulated (ratio < 1.0) differentially expressed proteins (DEPs) specific for female in C57BL/6J mice. Presented table was prepared based on proteins that were significantly different between compared groups. Statistics: Student’s t-test. Supplementary Table S18 refers to Figure S14 of the supplementary material.

| **No.** | **Protein ID** | **Protein annotation** | **Accession** | **Regulation** | **Ratio** |
| --- | --- | --- | --- | --- | --- |
| 1 | Ighv1-11 | Ighv1-11 | A0A0A6YWI9 | Up | 37.44 |
| 2 | Igkv8-16 | Immunoglobulin kappa variable 8-16 | A0A0G2JGT0 | Up | 17.80 |
| 3 | Thbs4 | Thrombospondin-4 | Q9Z1T2 | Down | 0.40 |
| 4 | Glycam1 | Glycosylation-dependent cell adhesion molecule 1 | Q02596 | Down | 0.38 |
| 5 | Il2rg | Cytokine receptor common subunit gamma | P34902 | Down | 0.31 |

**Table S19.** Ageing markers (respective DEPs) in murine plasma independent of strain but specific for sex of mice. Ratio of ageing (28-week-old vs 8-week-old) markers expression in murine aorta 2 upregulated (ratio ≥1.0) as well as 3 downregulated (ratio < 1.0) differentially expressed proteins (DEPs) specific for female in E3L.CETP mice. Presented table was prepared based on proteins that were significantly different between compared groups. Statistics: Student’s t-test. Supplementary Table S19 refers to Figure S14 of the supplementary material.

| **No.** | **Protein ID** | **Protein annotation** | **Accession** | **Regulation** | **Ratio** |
| --- | --- | --- | --- | --- | --- |
| 1 | Ighv1-11 | Ighv1-11 | A0A0A6YWI9 | Up | 20.52 |
| 2 | Igkv8-16 | Immunoglobulin kappa variable 8-16 | A0A0G2JGT0 | Up | 11.33 |
| 3 | Glycam1 | Glycosylation-dependent cell adhesion molecule 1 | Q02596 | Down | 0.49 |
| 4 | Il2rg | Cytokine receptor common subunit gamma | P34902 | Down | 0.45 |
| 5 | Thbs4 | Thrombospondin-4 | Q9Z1T2 | Down | 0.26 |

**Table S20.** Hyperlipidaemia markers (respective DEPs) in murine plasma independent of age but specific for sex of mice. Ratio of hyperlipidaemia (E3L.CETP vs C57BL/6J) markers expression in murine plasma 1 upregulated (ratio ≥1.0) as well as 6 downregulated (ratio < 1.0) differentially expressed proteins (DEPs) specific for male in 8-week-old mice. Presented table was prepared based on proteins that were significantly different between compared groups. Statistics: Student’s t-test. Supplementary Table S20 refers to Figure S16 of the supplementary material.

| **No.** | **Protein ID** | **Protein annotation** | **Accession** | **Regulation** | **Ratio** |
| --- | --- | --- | --- | --- | --- |
| 1 | Ighv1-4 | Immunoglobulin heavy variable 1-4 | A0A075B5T4 | Up | 1.90 |
| 2 | Serpinc1 | Antithrombin-III | P32261 | Down | 0.56 |
| 3 | Tfrc | Transferrin receptor protein 1 | Q62351 | Down | 0.49 |
| 4 | Apom | Apolipoprotein M | Q9Z1R3 | Down | 0.41 |
| 5 | Pltp | Phospholipid transfer protein | P55065 | Down | 0.40 |
| 6 | Apon | Apon | G3X9D6 | Down | 0.39 |
| 7 | Apoc3 | Apolipoprotein C-III | E9QP56 | Down | 0.33 |

**Table S21.** Hyperlipidaemia markers (respective DEPs) in murine plasma independent of age but specific for sex of mice. Ratio of hyperlipidaemia (E3L.CETP vs C57BL/6J) markers expression in murine plasma 1 upregulated (ratio ≥1.0) as well as 6 downregulated (ratio < 1.0) differentially expressed proteins (DEPs) specific for male in 28-week-old mice. Presented table was prepared based on proteins that were significantly different between compared groups. Statistics: Student’s t-test. Supplementary Table S21 refers to Figure S16 of the supplementary material.

| **No.** | **Protein ID** | **Protein annotation** | **Accession** | **Regulation** | **Ratio** |
| --- | --- | --- | --- | --- | --- |
| 1 | Ighv1-4 | Immunoglobulin heavy variable 1-4 | A0A075B5T4 | Up | 3.77 |
| 2 | Tfrc | Transferrin receptor protein 1 | Q62351 | Down | 0.58 |
| 3 | Serpinc1 | Antithrombin-III | P32261 | Down | 0.48 |
| 4 | Pltp | Phospholipid transfer protein | P55065 | Down | 0.35 |
| 5 | Apon | Apon | G3X9D6 | Down | 0.31 |
| 6 | Apom | Apolipoprotein M | Q9Z1R3 | Down | 0.27 |
| 7 | Apoc3 | Apolipoprotein C-III | E9QP56 | Down | 0.18 |

**Table S22.** Hyperlipidaemia markers (respective DEPs) in murine plasma independent of age but specific for sex of mice. Ratio of hyperlipidaemia (E3L.CETP vs C57BL/6J) markers expression in murine plasma 2 upregulated (ratio ≥1.0) as well as 1 downregulated (ratio < 1.0) differentially expressed proteins (DEPs) specific for female in 8-week-old mice. Presented table was prepared based on proteins that were significantly different between compared groups. Statistics: Student’s t-test. Supplementary Table S22 refers to Figure S16 of the supplementary material.

| **No.** | **Protein ID** | **Protein annotation** | **Accession** | **Regulation** | **Ratio** |
| --- | --- | --- | --- | --- | --- |
| 1 | Fah | Fumarylacetoacetase | P35505 | Up | 13.46 |
| 2 | Clec4f | C-type lectin domain family 4 member F | P70194 | Up | 9.52 |
| 3 | Antxr2 | Anthrax toxin receptor 2 | Q6DFX2 | Down | 0.10 |

**Table S23.** Hyperlipidaemia markers (respective DEPs) in murine plasma independent of age but specific for sex of mice. Ratio of hyperlipidaemia (E3L.CETP vs C57BL/6J) markers expression in murine plasma 2 upregulated (ratio ≥1.0) as well as 1 downregulated (ratio < 1.0) differentially expressed proteins (DEPs) specific for female in 28-week-old mice. Presented table was prepared based on proteins that were significantly different between compared groups. Statistics: Student’s t-test. Supplementary Table S23 refers to Figure S16 of the supplementary material.

| **No.** | **Protein ID** | **Protein annotation** | **Accession** | **Regulation** | **Ratio** |
| --- | --- | --- | --- | --- | --- |
| 1 | Clec4f | C-type lectin domain family 4 member F | P70194 | Up | 6.86 |
| 2 | Fah | Fumarylacetoacetase | P35505 | Up | 6.42 |
| 3 | Antxr2 | Anthrax toxin receptor 2 | Q6DFX2 | Down | 0.14 |
